# Bayesian regression and model selection for isothermal titration calorimetry with enantiomeric mixtures

**DOI:** 10.1371/journal.pone.0273656

**Published:** 2022-09-29

**Authors:** Trung Hai Nguyen, Van N. T. La, Kyle Burke, David D. L. Minh

**Affiliations:** 1 Laboratory of Theoretical and Computational Biophysics, Advanced Institute of Materials Science, Ton Duc Thang University, Ho Chi Minh City, Vietnam; 2 Faculty of Pharmacy, Ton Duc Thang University, Ho Chi Minh City, Vietnam; 3 Department of Biology, Illinois Institute of Technology, Chicago, IL, United States of America; 4 Department of Chemistry, Illinois Institute of Technology, Chicago, IL, United States of America; GLA University, INDIA

## Abstract

Bayesian regression is performed to infer parameters of thermodynamic binding models from isothermal titration calorimetry measurements in which the titrant is an enantiomeric mixture. For some measurements the posterior density is multimodal, indicating that additional data with a different protocol are required to uniquely determine the parameters. Models of increasing complexity—two-component binding, racemic mixture, and enantiomeric mixture—are compared using model selection criteria. To precisely estimate one of these criteria, the Bayes factor, a variation of bridge sampling is developed.

## Introduction

Isothermal titration calorimetry (ITC) is a solution-phase analytical technique that measures the heat absorbed or released due to a chemical reaction as a titrant is injected into a sample cell. As the reaction proceeds, the heat discharged or consumed in the sample cell modifies the power required to maintain it at the same temperature as a reference cell [1]. Kinetic models, e.g. for noncovalent binding [2], enzyme catalysis [3, 4], or covalent inhibition of enzymes [5], may be used to interpret the differential power. More often, the differential power is numerically integrated to yield an integrated heat of each injection. Models for the integrated heat based on equilibrium concentrations of chemical species are fit to the data to determine thermodynamic parameters of chemical reactions: the enthalpy Δ*H*, entropy Δ*S*, and Gibbs free energy Δ*G*. ITC is frequently applied to noncovalent binding between proteins and organic ligands [6], DNA/RNA [7, 8], lipids [9], and proteins [10]. It is also used to study the protonation and tautomerization of binding partners [11, 12].

As recently reviewed by Werberg and Mastai [13], ITC has been used to study chiral interactions. Although enantiomers—molecules with mirror-image chirality—have the same chemical composition and similar structures, they may have significantly different bioactivities, metabolic rate, metabolites, excretion, potency, receptor preference, interactions with transporters and enzymes, and toxicity [14]. ITC has been used in many types of chiral studies [13], including: comparing enthalpies of injecting two enantiomers, phenyl-*α*-L- and phenyl-*α*-D- mannopyranoside, into a sample cell with molecularly imprinted polymers [15]; measuring the enthalpy of dilution and pairwise interaction coefficients of enantiomers [16, 17]; and determining thermodynamic parameters of binding galactonoamidine derivatives to chiral organometallic complexes [18]. In contrast to other common chiroptical methods that strongly restrict experimental conditions, ITC is a simple label-free technique that can yield a complete set of thermodynamic parameters relevant to chiral interactions, characterize chiral selectivity, and investigate the formation of chiral complexes [13].

Although ITC measurements for binding thermodynamics (including studies of chiral systems) are typically performed with a single binding species in the titrant and titrand, other experimental designs have been investigated. In 2006, Fokkens *et. al*. described a protocol in which an enantiomeric mixture is injected into the sample cell [19]. While it is generally beneficial to separate the compounds prior to analysis, a preliminary determination of thermodynamic parameters without separation can save time and money. For example, racemic mixtures (which have a 1:1 ratio of enantiomers) of aminoadamantane derivatives were titrated with the M2 proton channel of influenza A [20, 21]. Fokkens *et. al*. demonstrated that if binding affinities of two enantiomers are sufficiently distinct, two distinct affinities can be determined by fitting a simple binding model to different regions of the isotherm. In 2012, Krainer *et. al*. described an experimental protocol that is essentially opposite: a macromolecular receptor was the titrant and the titrand contains a dilute mixture of two competing ligands (which were not enantiomers) [22]. Fitting data to an analytical expression for the concentration of each complex, they were able to obtain accurate and precise dissociation constants and binding enthalpies for both ligands.

One common shortcoming of methods to analyze data from ITC (and many other analytical instruments) is underestimation of statistical uncertainty. Determination of thermodynamic parameters from ITC is a nonlinear regression problem that is typically implemented by maximizing the likelihood of observing the data. If the fitting procedure does not allow variation in a parameter (e.g. concentrations of titrand or titrand), then the uncertainty of these quantities is not accounted for in the asymptotic standard error. The problem is quite general; Petr Kuzmic̆, developer of the enzymology software package DynaFit, wrote “formal standard errors can (and usually do) grossly underestimate the statistical uncertainty” [23]. Indeed, multiple studies have indicated that the Origin software package included with the MicroCal VP-ITC instrument and commonly used to analyze ITC data by nonlinear least squares regression does not account for all relevant sources of error [24–26]. For this reason, it is common practice in ITC data analysis to perform replicates of the measurement (usually at least in triplicate) and report the standard deviation of multiple maximum likelihood estimates rather than the formal standard error. In the ABRF-MIRG‘02 study, in which the same sample was analyzed by 14 biomolecular resource facilities, the standard deviation of replicate analyses yielded much larger and more accurate uncertainty intervals than the standard error from nonlinear regression [27].

On the other hand, if fixing parameters that contribute to error can lead to the underestimation of uncertainty, including additional parameters that increase the complexity of the statistical model runs the risk of overfitting the data. In ITC experiments, additional parameters that could be reasonable are those that describe the concentrations of species in the titrant or titrand and thermodynamic parameters for more complex reactions. If the titrant includes a mixture of enantiomers, it is plausible for the enantiomers to be racemic or to be optically active, present in different concentrations. Moreover, several chemical reactions may occur in the solution. If only a single enantiomer binds to the receptor or if the enantiomers bind with equal affinity, a two-component binding model is the most appropriate. If they bind with different affinity, a competitive binding model is most appropriate. It is nearly always the case that a more complex statistical model will reduce the residual. However, an excessively complex statistical model may not improve or may even deteriorate the quality of fit to additional data from the same system, especially if it is measured with a different experimental protocol.

Bayesian statistics provides a theoretical framework to address these interrelated issues of uncertainty quantification and model selection. Uncertainty in any quantity that can contribute to estimation error may be incorporated as an additional parameter in the model. For example, we recently developed a Bayesian regression method for the analysis of ITC data with a two-component binding model [28]. In MicroCal’s nonlinear regression for such data, the titrant concentration is fixed and the titrand concentration (via the site parameter *N*) is allowed to freely vary. Hence, the standard error is severely underestimated. By treating both the titrant and titrand concentrations as variables, we were able to improve the estimation of uncertainty; we obtained Bayesian credible intervals that were larger and much more consistent with observed confidence intervals. While our analysis included a larger number of parameters than the standard approach, overfitting was not an issue when we used an informative prior for the concentrations of both species: a lognormal distribution centered at the stated concentration. Even with an uninformative prior, the model selection problem may be addressed by the Bayes factor [29, 30], which compares the odds of the data being produced by two models irrespective of specific values of model parameters. In addition to the Bayes factor, other commonly-used model selection criterion include the Bayesian information criterion (BIC) [31], an approximation to the Bayes factor that assumes that the posterior is a multivariate Gaussian, and the Akaike information criterion (AIC) [32]. Both the AIC and BIC include the log likelihood of the maximum likelihood estimate and penalties for a larger number of parameters.

Besides Bayesian regression, another strategy to address the underestimation of uncertainty in nonlinear regression based on maximum likelihood estimation (but not model selection) is error propagation. In error propagation, some parameters that could contribute to the uncertainty of estimated values are not explicitly fitted. Rather, the error in these parameters is propagated to the uncertainty of estimated values based on a first-order Taylor series expansion. Boyce *et. al*. [33] suggested that the error in titrant concentration could be propagated to estimates of thermodynamic quantities. While this error propagation does expand confidence intervals, the authors did not demonstrate, in either simulations or experiments, that the expanded intervals accurately reflect the uncertainty of thermodynamic parameters.

In the present contribution, we perform Bayesian regression and model selection on ITC data in which a mixture of enantiomers is titrated into a solution with a single receptor. In addition to the two-component binding model (2C), we consider models in which the titrant contains a racemic mixture (RM) with equal amount of each enantiomer or an enantiomeric mixture (EM) with optical activity. Moreover, we introduce a new way (to our knowledge) to use bridge sampling [34] to precisely compute Bayes factors in nested statistical models. Finally, we use Bayes factors as well as the BIC and AIC to determine which models are best supported by the data.

## Materials and methods

### Models for ITC data

Data from an ITC experiment consists of a series of measured injection heats, $D=\{q_{1},q_{2},\ldots ,q_{N}\}$, where *N* is the number of injections. Measured injection heats may be treated as the sum of the measurement error and model integrated heat. As in our previous work [28], we make the common assumption that measurement error is independently and identically distributed with a Gaussian distribution. Model integrated heats depend on a set of parameters, denoted as ***θ***. The parameters comprising ***θ*** depend on the specific binding model.

In all three binding models that we use here—2C, RM, and EM—the parameters include the initial concentration of receptor in the sample cell, [*R*]_0_, and the total concentration of ligand in the syringe, [*L*]_*s*_. They also include Δ*H*_0_, the heat of dilution and stirring per injection, and *σ*, the standard deviation in the measurement error of each integrated heat. Additional thermodynamic parameters and mixture composition parameters for the 2C, RM, and EM models are described below:

1. The 2C binding model assumes that only one ligand binds with the receptor. In this case, the model parameters are [28],
  $$\begin{array}{c} \theta^{2C}\equiv \left(\Delta G,\Delta H,\Delta H_{0},{\left[R\right]}_{0},{\left[L\right]}_{s},\sigma \right), \end{array}$$ (1)
  where Δ*G* and Δ*H* are the standard free energy and enthalpy of binding, respectively. While it is often customary to denote standard thermodynamic quantities with a superscript ° or ^*θ*^, for the sake of notational simplicity we omit these labels in this manuscript.
2. The RM model assumes that the titrant contains a mixture of two different ligands with possibly different Δ*G* and Δ*H*. The relative composition of the two ligands is assumed to be fixed at 0.5. In this case, the model parameters are,
  $$\begin{array}{c} \theta^{RM}\equiv \left(\Delta G_{1},\Delta \Delta G,\Delta H_{1},\Delta H_{2},\Delta H_{0},{\left[R\right]}_{0},{\left[L\right]}_{s},\sigma \right), \end{array}$$ (2)
  where Δ*G*_1_ is the binding free energy of the higher-affinity ligand, ΔΔ*G* ≡ Δ*G*_2_ − Δ*G*_1_ is the difference in binding free energy between the second and the first ligands. Without loss of generality, we assume that ΔΔ*G* is non-negative, ΔΔ*G* ≥ 0. Δ*H*_1_ and Δ*H*_2_ are enthalpies of binding the first and second ligands, respectively.
3. The EM model is the same as the RM model except that the mixture composition *ρ* is a free variable varying between 0 and 1. The parameters in this case are
  $$\begin{array}{c} \theta^{EM}\equiv \left(\Delta G_{1},\Delta \Delta G,\Delta H_{1},\Delta H_{2},\Delta H_{0},{\left[R\right]}_{0},{\left[L\right]}_{s},\rho ,\sigma \right). \end{array}$$ (3)

Obtaining the theoretical heat of injection from model parameters also requires the experimental protocol of injection volumes and models for concentrations prior to reaction and at equilibrium. Concentrations prior to reaction were based on the perfusion model [35]. For the 2C model, equilibrium concentrations were based on a quadratic expression, as previously described [28]. For the RM and EM binding models, we used an analytical expression for equilibrium concentrations of the competitive binding model [22, 36]. Mathematical details of the binding models are included in S1 Appendix.

### Simulation

To assess whether Bayesian credible intervals from our analysis accurately reflect the uncertainty of parameters, we simulated 50 ITC experiments in which an enantiomeric mixture is titrated into the cell. In each simulated experiment, [*L*]_*s*_ and [*R*]_0_ were drawn from a lognormal distribution with stated values of 1.0 and 0.05 mM, respectively, and with an uncertainty of 10%. Model integrated heats were calculated using the EM model with Δ*G*_1_ = −11.5 kcal/mol, ΔΔ*G* = 4 kcal/mol, Δ*H*_1_ = −7 kcal/mol, Δ*H*_2_ = −2 kcal/mol, Δ*H*_0_ = 0.5 *μ*cal/mol, and *ρ* = 0.5.

### Data curation

**Fig 1:**
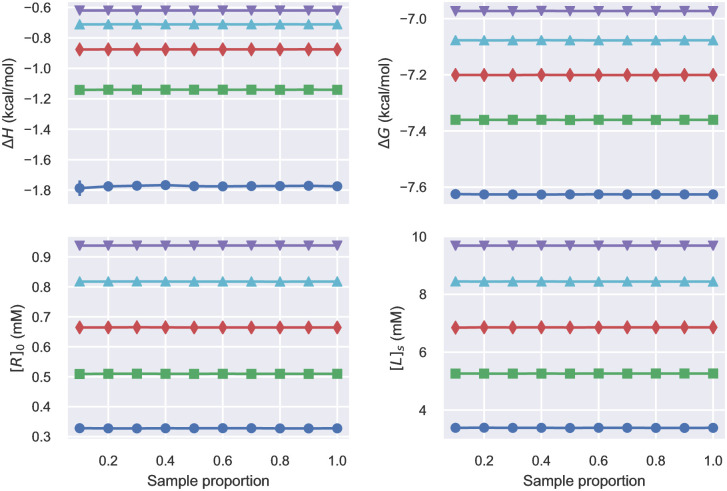
Convergence of percentiles of the Bayesian posterior of the 2C model based on the Fokkens_1a dataset. 20,000 samples were drawn from the Bayesian posterior using the NUTS sampler. Four key parameters are shown. Lines correspond to the 5-th (blue circle), 25-th (green square), 50-th (red diamond), 75-th (cyan upward triangle) and 95-th (magenta downward triangle) percentile. The error bars, which are too small to be visible, are standard deviations estimated by 100 bootstrapping samples. Similar plots for RM and EM models are shown in S1 and S2 Figs, respectively. S2 Fig also shows the convergence of percentiles for fits to a representative simulation of the EM model.

We also analyzed 11 experimental ITC curves. Five of them were reported in Figure 1 of Fokkens *et. al*. [19] (we denote them as Fokkens_1a, Fokkens_1b, .., Fokkens_1e). The remaining six were extracted from figures 57, 59, and 60 of Bernhard Baum’s PhD dissertation [37]. These are denoted as Baum_57, Baum_59, Baum_60_1, .., Baum_60_4. Because the original data were no longer available from the authors, we digitized the integrated heats from the figures using the WebPlotDigitizer web site (https://automeris.io/WebPlotDigitizer/). We also collected information about the systems and experimental conditions (see S1 Table). Concentrations of macromolecule in the sample cell and of small molecule ligand(s) in the syringe were available for 7 datasets. For the other 4 datasets, we were unable to locate concentrations of either titrand, titrant, or both. Fokkens *et. al* [19] and Baum *et. al*. [37] used MCS-ITC and VP-ITC instruments made by Microcal Inc., Northhampton, MA, USA to carry out the ITC experiments. Fokkens *et. al* [19] did not specify the experiment temperature but Baum *et. al*. [37] explained that measurements in the lab are routinely performed at 298 K. All datasets were analyzed assuming a temperature of 300 K. The cell volume of the calorimeters is 1.3513 mL [38].

### Bayesian regression

Bayesian regression using ITC data to determine parameters for binding models was performed similarly to our previous work [28]. The posterior probability of the parameters given the data is expressed with Bayes’ rule,

$$\begin{array}{c} p(\theta |D)\propto p(D|\theta )\;p(\theta ), \end{array}$$ (4)

where p(D|θ) is the likelihood of observing the data given the parameters and *p*(***θ***) is the prior probability of the parameters. Based on the assumption that measurement error is independently and identically distributed with a Gaussian distribution, the likelihood is,

$$\begin{array}{c} p\left(D|\theta \right)=\frac{1}{{\left(2\pi \right)}^{N/2}\sigma^{N}}\text{exp}[-\frac{1}{2\sigma^{2}}\sum_{n=1}^{N}{\left(q_{n}-q_{n}^{*}\left(\theta \right)\right)}^{2}], \end{array}$$ (5)

where $q_{n}^{*}\left(\theta \right)$ is the theoretical heat of injection *n*.

We assume that the parameters ***θ*** are independent from one another and, therefore, the prior *p*(***θ***) is a product of priors of individual parameters, *p*(***θ***) = ∏_*i*_ *p*(*θ*_*i*_). The priors for Δ*G*, Δ*G*_1_, ΔΔ*G*, Δ*H*, Δ*H*_1_, Δ*H*_2_ (in kcal/mol) and Δ*H*_0_ (in calories) were chosen to be uniform,

$$\begin{array}{c} \Delta G,\Delta G_{1}∼\text{Uniform}\left(-40,40\right),\;\;\;\Delta \Delta G∼\text{Uniform}\left(0,40\right), \end{array}$$ (6)

$$\begin{array}{c} \Delta H,\Delta H_{1},\Delta H_{2}∼\text{Uniform}\left(-100,100\right), \end{array}$$ (7)

$$\begin{array}{c} \Delta H_{0}∼\text{Uniform}\left(q_{min}-\Delta q,q_{max}+\Delta q\right), \end{array}$$ (8)

where *q*_*min*_ = min{*q*_1_, *q*_2_, …, *q*_*N*_}, *q*_*max*_ = max{*q*_1_, *q*_2_, …, *q*_*N*_}, and Δ*q* = *q*_*max*_ − *q*_*min*_.

Priors for cell [*R*]_0_ and syringe [*L*]_*s*_ concentrations (in nM) are either log-normal or uniform. If the stated value is available (see S1 Table) then the log-normal prior was used,

$$\begin{array}{c} \text{ln}{\left[X\right]}_{0}∼N\left(\mu ={\left[X\right]}_{0}^{\text{stated}},\sigma =0.1*{\left[X\right]}_{0}^{\text{stated}}\right), \end{array}$$ (9)

where ${\left[X\right]}_{0}^{\text{stated}}\in \left\{{\left[R\right]}_{0}^{\text{stated}},{\left[L\right]}_{s}^{\text{stated}}\right\}$. Otherwise, the uniform prior was used,

$$\begin{array}{c} {\left[R\right]}_{0}∼\text{Uniform}\left(0.001,1\right),\;\;\;{\left[L\right]}_{s}∼\text{Uniform}\left(0.01,10\right). \end{array}$$ (10)

These concentration priors are appropriate for the analysis of single experiments. If multiple experiments are performed using the same stock solutions, then concentration parameters could be shared across all the pseudo-independent replicates. If new solutions are prepared for each replicate, then it is appropriate to use independent concentration parameters for each measurement.

The parameter *ρ* (dimensionless) in the EM model also has uniform prior,

$$\begin{array}{c} \rho ∼\text{Uniform}(0,1). \end{array}$$ (11)

Finally, the prior for the standard deviation of the measurement error *σ*, a nuisance parameter, was chosen to be an uninformative Jeffreys prior [39],

$$\begin{array}{c} p\left(\sigma \right)\propto \frac{\sigma_{0}}{\sigma }, \end{array}$$ (12)

where *σ*_0_ = 1 cal, an arbitrary constant to make $\frac{\sigma_{0}}{\sigma }$ a dimensionless quantity.

The No-U-Turn sampler (NUTS) [40] was used to sample from posterior distributions. NUTS is an extension of Hamiltonian Monte Carlo [41], which uses trajectories akin to molecular dynamics simulations to generate candidates for Markov chain Monte Carlo. Hamiltonian Monte Carlo has a tuning problem in which it may suffer from random walk behavior if the number of integration steps *L* is set too small or waste computing time if *L* is set too large. NUTS automatically selects an optimal value for *L* for each move by stopping the integrator when the trajectory starts to trace back its steps. We used the implementation of NUTS in PyMC3 [42].

After a warmup of 10,000 NUTS moves, we collected 200,000 samples for the 2C model and 600,000 samples for the RM and EM models. Samples from the posterior were thinned by a factor of 10, retaining only every 10 samples, resulting in 20,000 samples for the 2C model and 60,000 samples for the RM and EM models. Neglecting warmup samples reduces bias due to the initial state and thinning reduces the correlation between Markov chain Monte Carlo samples. When analyzing the simulations, we initiated the Markov chain using the true values.

### Model selection

The model best supported by each dataset was assessed via Bayes factors, BIC, and AIC. A Bayes factor quantifies the odds of observing the data D given two models $M_{1}$ and $M_{2}$. It is defined as a ratio of the likelihood of the data D given model $M_{2}$ over the likelihood of the data D given model $M_{1}$,

$$\begin{array}{ccc} R & = & \frac{p(D|M_{2})}{p(D|M_{1})}. \end{array}$$ (13)

The BIC and AIC are commonly used criteria for model selection, defined as,

$$\begin{array}{c} AIC=-2\;\text{ln}[\underset{\theta }{\text{max}}\;p\left(D|\theta \right)]+2k, \end{array}$$ (14)

$$\begin{array}{c} BIC=-2\;\text{ln}[\underset{\theta }{\text{max}}\;p\left(D|\theta \right)]+k\;\text{ln}\left(N\right), \end{array}$$ (15)

where *k* is the number of free parameters and *N* is the number of data samples. While the BIC and AIC are straightforward to compute from the maximum likelihood estimate, precise estimation of Bayes factors is more challenging.

We computed Bayes factors by using bridge sampling [34] in a new way. Bridge sampling is the statistical generalization of the Bennett Acceptance Ratio (BAR) [43], which was derived to compute the free energy difference between a pair of thermodynamic states based on samples from molecular simulations in each state (see S1 Appendix). A free energy difference is the negative logarithm of the ratio of normalizing constants for the Boltzmann distribution of molecular configurations. Bayes factors may also be expressed as ratios of normalizing constants, but for posterior probability distributions,

$$\begin{array}{ccc} R & = & \frac{\int p\left(\theta_{2}|M_{2}\right)p\left(D|\theta_{2},M_{2}\right)d\theta_{2}}{\int p\left(\theta_{1}|M_{1}\right)p\left(D|\theta_{1},M_{1}\right)d\theta_{1}} \end{array}$$ (16)

$$\begin{array}{ccc} & = & \frac{\int p_{2}\left(\theta_{2}\right)d\theta_{2}}{\int p_{1}\left(\theta_{1}\right)d\theta_{1}}. \end{array}$$ (17)

Here, ***θ***_1_ and ***θ***_2_ are parameters of models $M_{1}$ and $M_{2}$, respectively. $p(\theta |M_{i})$ is the prior distribution of ***θ*** and p(D|θ) is the likelihood of D given ***θ***. To simplify the notation, the unnormalized posteriors of ***θ***_1_ and ***θ***_2_ are defined as $p_{1}\left(\theta_{1}\right)\equiv p\left(\theta_{1}|M_{1}\right)p\left(D|\theta_{1}\right)$ and $p_{2}\left(\theta_{2}\right)\equiv p\left(\theta_{2}|M_{2}\right)p\left(D|\theta_{2}\right)$.

In order to use bridge sampling, the integrals in the numerator and denominator of Eq 17 must be taken over the same variables. Thus, we reformulate Eq 17 such that it satisfies this requirement. Assume that the models $M_{1}$ and $M_{2}$ are nested and $M_{2}$ is more complex than $M_{1}$, i.e. ***θ***_2_ contains more parameters than ***θ***_1_. In this case, the parameters in ***θ***_1_ are a subset of parameters in ***θ***_2_, ***θ***_2_ = (***θ***_1_, *γ*). In terms of ***γ***, the Bayes factor is,

$$\begin{array}{ccc} R & = & \frac{\int p_{2}\left(\theta_{1},\gamma \right)d\theta_{1}d\gamma }{\int p_{1}\left(\theta_{1}\right)d\theta_{1}} \end{array}$$ (18)

$$\begin{array}{ccc} & = & \frac{\int p_{2}\left(\theta_{1},\gamma \right)d\theta_{1}d\gamma }{\int p_{1}\left(\theta_{1}\right)f\left(\gamma \right)d\theta_{1}d\gamma } \end{array}$$ (19)

Eq 19 is obtained from Eq 18 by multiplying the denominator by ∫ *f*(***γ***)*d**γ*** = 1, where *f*(***γ***) is a proposal distribution, a normalized probability density from which random variates can be easily generated.

Computing the Bayes factor with bridge sampling requires drawing samples from two distributions and computing the ratio of probabilities that the sample would be drawn from each distribution. The two distributions are the posterior probability *p*_2_(***θ***_1_, ***γ***) and the joint probability *p*_1_(***θ***_1_)*f*(***γ***). For the latter, samples from *p*_1_(***θ***_1_) generated by Markov chain Monte Carlo may be supplemented by independent and identically distributed random variates from *f*(***γ***). The ratio of probabilities that the sample would be drawn from each distribution may be expressed as an exponentiated potential energy difference. The potential energy, or negative logarithm of the unnormalized probability, is, *u*_1_(***θ***_1_, ***γ***) ≡ − ln *p*_1_(***θ***_1_) − ln *f*(***γ***) and, *u*_2_(***θ***_1_, ***γ***) ≡ − ln *p*_2_(***θ***_1_, ***γ***). Differences between *u*_1_ and *u*_2_ are computed for each sample from the two distributions and used in Eq. A.34 of S1 Appendix to estimate the Bayes factor.

In principle, *f*(***γ***) can be any probability distribution from which random variates can be easily generated, e.g. a uniform distribution. However, if random samples from *p*_1_(***θ***_1_)*f*(***γ***) have a low probability in *p*_2_(***θ***_1_, ***γ***), then the estimator of the Bayes factor may require a prohibitive number of samples to converge. To increase overlap between the distributions in the numerator and denominator of Eq 19, we chose *f*(***γ***) to be a multivariate Gaussian with a mean vector from the sample mean, $\bar{\gamma }$, and covariance matrix from the sample covariance, $\bar{\sigma_{\gamma }^{2}}$, of NUTS samples drawn from *p*_2_(***θ***_1_, ***γ***).

## Results and discussion

### Estimated posteriors and Bayes factors are converged

Robust Bayesian analysis requires adequate sampling from the posterior such that summary statistics are converged, unaffected by additional sampling. Convergence of sampling the Bayesian posteriors was evaluated based on the 5-th, 25-th, 50-th, 75-th and 95-th percentiles of the marginal probability of key parameters as the number of Monte Carlo samples is increased (Fig 1, S1 and S2 Figs). As the number of samples increases, the percentiles change very little, with negligible estimated standard errors. This convergence indicates that the posterior distributions have been thoroughly sampled after a small number of samples from the posterior.

**Fig 2:**
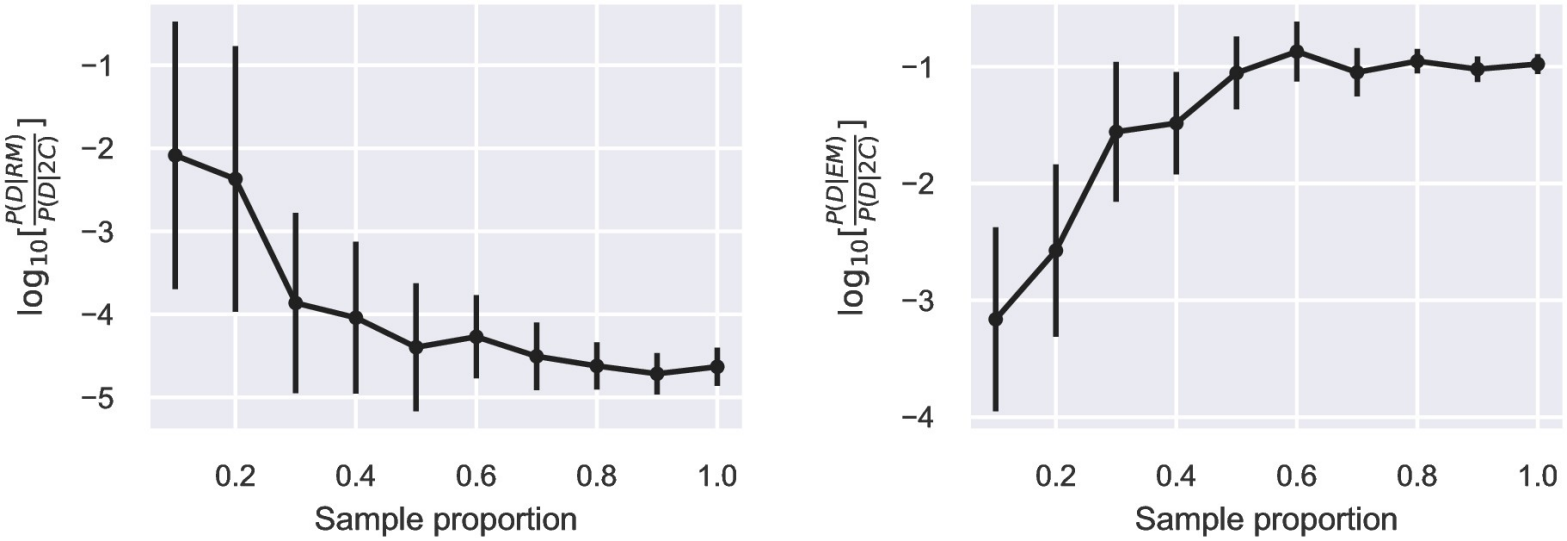
Convergence of Bayes factors for the Fokkens_1a dataset. Bayes factors were estimated based on 20,000 NUTS samples for the 2C model and 60,000 NUTS samples for the RM and EM models. The error bars are standard deviations estimated by 1000 bootstrapping samples. Similar plots for the 10 other datasets are shown in the Supporting Information (S3 to S12 Figs).

In comparison, estimates of the Bayes factors based on bridge sampling converge more slowly. For all datasets, the estimated Bayes factors start to level off after about 60% of the total Monte Carlo samples (Fig 2 and S3 to S12 Figs). Convergence provides confidence in using Bayes factors for model selection.

A possible reason that the Bayes factor is not used more widely is that it is difficult to estimate precisely. At least with the present data and statistical models, the novel approach to using bridge sampling for nested models appears to be resolve this issue.

The variation of bridge sampling described here is related to but not equivalent to an approach that has been previously described [44, 45]. In these related works, bridge sampling was used to evaluate the marginal likelihood of a single model, opposed to Bayes factors, which are the ratio of marginal likelihoods for two models. Hence, proposal distributions included all degrees of freedom, opposed to a subset that are present in one model but not the other. As we have done here, the authors suggested that a normal distribution with the first two moments selected to match the posterior is usually a suitable proposal distribution.

### Concentration priors have distinct impacts on posteriors

While it is possible to sample from the Bayesian posterior and obtain converged summary statistics without knowing the concentration of [*L*]_*s*_ or [*R*]_0_ or even both, uninformative priors generally lead to broader posteriors.

**Fig 3:**
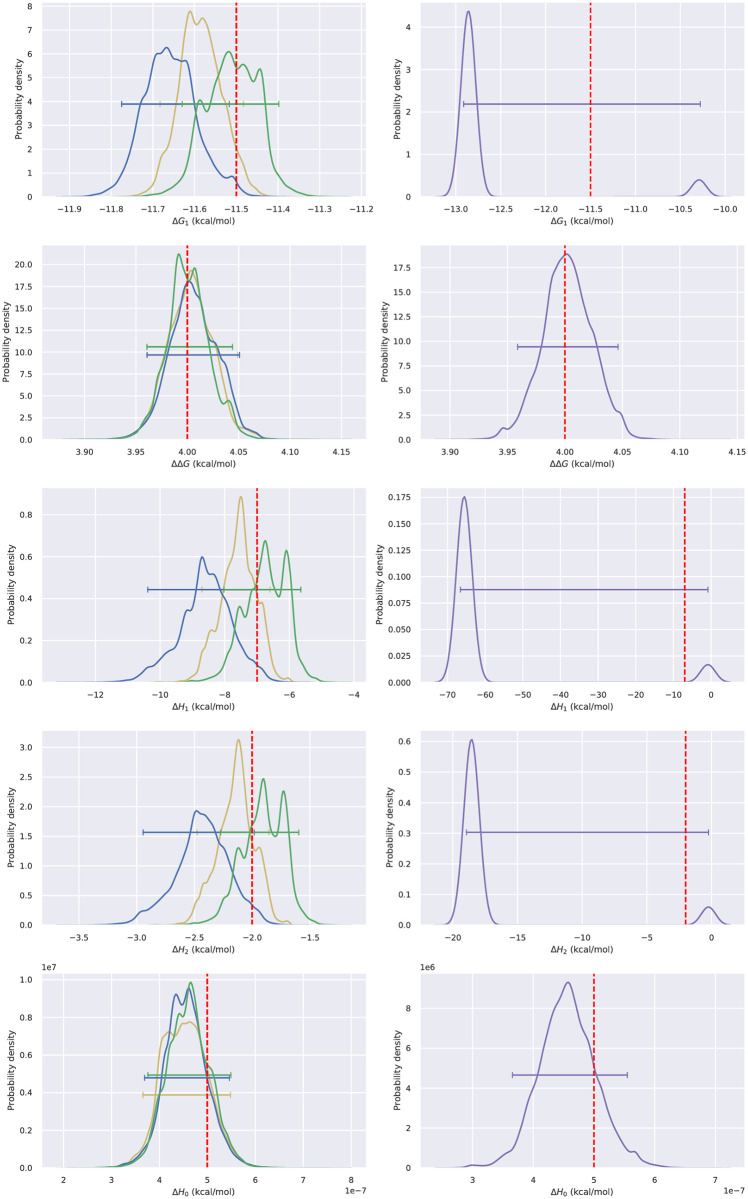
Representative 1D histograms of thermodynamic parameters sampled from the Bayesian posterior with four concentration priors. Priors for the concentration were lognormal for both [*L*]_*s*_ and [*R*]_0_ (yellow line), lognormal for [*L*]_*s*_ and uniform for [*R*]_0_ (green line), uniform for [*L*]_*s*_ and lognormal for [*R*]_0_ (blue line), or uniform for both [*L*]_*s*_ and uniform for [*R*]_0_ (purple line). Integrated heats are from a representative simulation from the EM model. Horizontal bars show 95% Bayesian credible intervals. True values for parameters were shown as red dashed line.

In our simulations, as shown by histograms (Fig 3), standard deviations (Table 1), and root mean square errors (Table 2) of the 1D marginals (Table 1), using an informative lognormal prior for both [*L*]_*s*_ or [*R*]_0_ leads to the most accurate and precise posteriors. If the receptor concentration [*R*]_0_ is unknown and a uniform prior is used, the standard deviations and root mean square errors for all thermodynamic parameters are roughly doubled. Similar behavior is observed if the ligand concentration is missing, with the exception of ΔΔ*G*, which retains a comparable accuracy and uncertainty. If both the ligand and receptor concentration are missing, then posteriors of most parameters are broader than if a single concentration is missing. It is still possible to determine the binding free energies Δ*G*_1_ and ΔΔ*G*, the latter which is surprisingly precise, but without concentrations the posterior of the enthalpies Δ*H*_1_ and Δ*H*_2_ are so broad that the values are essentially unknown.

**Table 1:** Mean and standard deviation (in parentheses) of the Bayesian posterior with four concentration priors, based on the 50 simulations from the EM model.

[*R*]_0_ prior	lognormal	uniform	lognormal	uniform
[*L*]_*s*_ prior	lognormal	lognormal	uniform	uniform
[*R*]_0_	4.84E-2 (3.80E-3)	4.79E-2 (7.44E-3)	4.94E-2 (4.74E-4)	1.43E-1 (7.23E-2)
[*L*]_*s*_	1.01E+0 (8.08E-2)	9.90E-1 (1.19E-2)	1.05E+0 (1.67E-1)	2.97E+0 (1.40E+0)
Δ*G*_1_	-1.15E+1 (4.63E-2)	-1.15E+1 (6.62E-2)	-1.15E+1 (7.11E-2)	-1.20E+1 (4.04E-1)
ΔΔ*G*	4.00E+0 (2.71E-2)	4.00E+0 (3.89E-2)	4.00E+0 (2.91E-2)	4.01E+0 (7.53E-2)
Δ*H*_1_	-7.04E+0 (4.27E-1)	-7.18E+0 (7.53E-1)	-6.89E+0 (8.72E-1)	-4.45E+1 (9.99E+0)
Δ*H*_2_	-2.01E+0 (1.26E-1)	-2.07E+0 (2.17E-1)	-1.99E+0 (2.20E-1)	-1.28E+1 (2.87E+0)
Δ*H*_0_	4.97E-7 (3.49E-8)	4.88E-7 (6.61E-8)	4.87E-7 (7.60E-8)	4.96E-7 (3.64E-8)
*ρ*	5.00E-1 (1.97E-3)	4.99E-1 (5.27E-3)	4.99E-1 (4.95E-3)	5.00E-1 (4.74E-3)

**Table 2:** Root mean square error of the mean. The root mean square error, or root mean square difference between the estimate and the true value, of the mean parameter value from the Bayesian posterior.

[*R*]_0_ prior	lognormal	uniform	lognormal	uniform
[*L*]_*s*_ prior	lognormal	lognormal	uniform	uniform
[*R*]_0_	4.08E-03	7.65E-03	7.30E-04	1.18E-01
[*L*]_*s*_	8.10E-02	1.56E-02	1.72E-01	2.41E+00
Δ*G*_1_	4.66E-02	6.81E-02	7.06E-02	6.50E-01
ΔΔ*G*	2.70E-02	3.85E-02	2.88E-02	7.54E-02
Δ*H*_1_	4.25E-01	7.67E-01	8.70E-01	3.88E+01
Δ*H*_2_	1.25E-01	2.27E-01	2.18E-01	1.11E+01
Δ*H*_0_	3.47E-08	6.65E-08	7.64E-08	3.62E-08
*ρ*	1.99E-03	5.34E-03	5.02E-03	4.70E-03

The impact of missing concentration information is also evident in the analysis of experimental data. As shown by 1D and 2D histograms of NUTS samples (S2 Appendix for 2C, S3 Appendix for RM, and S4 Appendix for EM), it is possible to estimate thermodynamic parameters even if the concentration is unknown and a uniform prior is used. However, this lack of information has consequences. In these cases (Fokkens_1a, Fokkens_1b, Baum_57, and Baum_59), the posterior for the unknown concentrations is broad. Additionally, posteriors of the thermodynamic parameters may change shape. In our previous work fitting with the 2C model and using known concentrations [28], posteriors for thermodynamic parameters were all close to symmetric, with only subtle skew, and Gaussian. Here, when fitting the 2C model with unknown concentrations, many posteriors for Δ*G* and Δ*H* are highly skewed (S2 Appendix). Finally, the lack of a nominal concentration may also shift the peak of posteriors for thermodynamic parameters. In a previous fit to the Fokkens_1a dataset in which the solution concentrations presumably were specified, the dissociation constant was found to be 43.9 × 10^5^ M^−1^ [19], which corresponds to Δ*G* = -9.1 kcal/mol. In contrast, the Bayesian posterior for Δ*G* has samples in the range of -7.5 and -7 kcal/mol. Other datasets in which the concentration is presently unknown clearly have a step in the integrated heat and were fit with two independent simple binding events. Thus, the model was distinct and parameters are not directly comparable to the present results.

### Many Bayesian posteriors have complex structure

The histograms of NUTS samples also show that many of the Bayesian posterior distributions have complex structure. The simplest posteriors are from the 2C model. For the Fokkens_1a, Fokkens_1e, and Baum_60_2 datasets, in which the integrated heat is a sigmoidal function, the posteriors show simple unimodal peaks and essentially linear correlation between parameters. Similar behavior was observed in our previous Bayesian analysis for two-component binding processes [28]. For some other datasets such as Baum_57 and Fokkens_1b, the posteriors given by the 2C model have nonlinear correlations between parameters. Posteriors based on the RM and EM model are mostly complex, with multimodal and skewed peaks, and nonlinear relationships between parameters (S3 and S4 Appendices). Exceptions include analyses of the simulation data sets with informative concentration priors and of dataset Fokkens_1d, which show a rather simple posterior for both RM and EM models. In the representative simulation of the EM model, when a uniform prior is used for both the ligand and receptor concentration, the posterior becomes multimodal.

Broad or multimodal posteriors indicate that additional data is required to uniquely determine model parameters. The ability to reveal ambiguity in parameter fitting is the key advantage of the Bayesian approach over maximum likelihood estimation.

### Bayesian credible intervals can be accurate confidence intervals for the EM model

Bayesian credible intervals were assessed by plotting the fraction of intervals that contain the true value against the stated confidence level. Similar plots are Figure 8 of Nguyen et. al. [28] and Figure 1 of Minh and Makowski [46]. If the credible intervals are good confidence intervals, then the data points should lie on the diagonal. Points below the diagonal indicate that the credible intervals underestimate the error. Conversely, points above the diagonal suggest that they overestimate the error.

**Fig 4:**
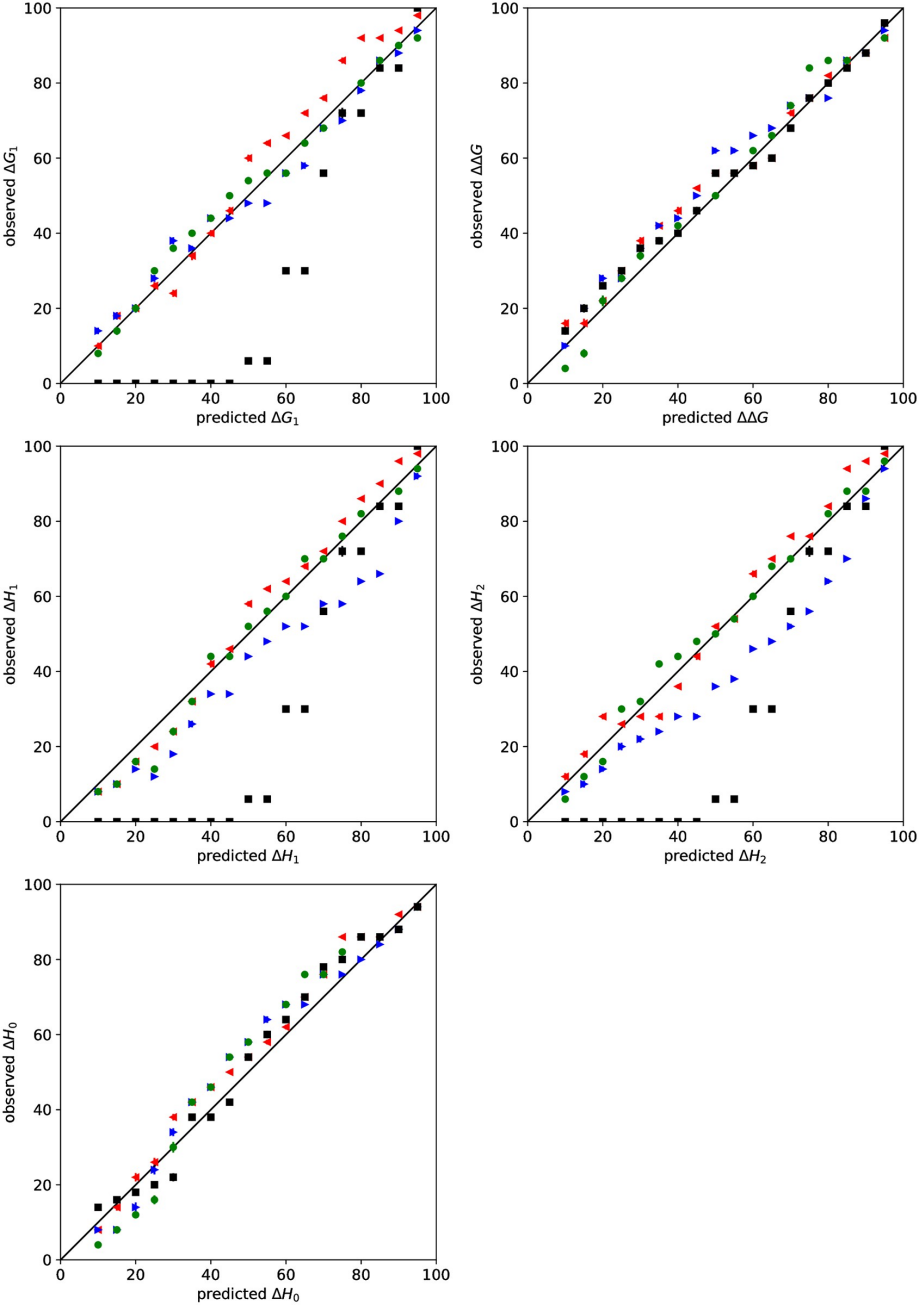
Uncertainty validation for Bayesian analysis of simulated data. The predicted versus observed rate (%) in which BCIs contain the true value for binding parameters are shown. Priors for the concentration were lognormal for both [*L*]_*s*_ and [*R*]_0_ (red left triangle), lognormal for [*L*]_*s*_ and uniform for [*R*]_0_ (green circle), uniform for [*L*]_*s*_ and lognormal for [*R*]_0_ (blue right triangle), or uniform for both [*L*]_*s*_ and uniform for [*R*]_0_ (black square). Error bars are standard deviations based on bootstrapping of 1000 samples.

If concentrations are specified, then Bayesian credible intervals are accurate confidence intervals (Fig 4). If the ligand concentration [*L*]_*s*_ is missing, then the credible intervals of the concentrations and enthalpies somewhat underestimate the confidence intervals. If the receptor concentration [*R*]_0_ is missing, then most confidence intervals are accurately estimated but the smaller confidence intervals of concentrations are underestimated. Missing both the ligand and receptor concentration leads to significant underestimation of uncertainties for concentrations and thermodynamic parameters.

### Bayes factors are the most accurate model selection criterion

For the 11 datasets, calculated model selection criterion did not always yield consistent results (see S2 Table). While Bayes factors and BIC were consistent for most datasets, the AIC favored the more complex EM and RM models. In the following discussion, we categorize the datasets into four groups according to Bayes factors (Table 3).

**Table 3:** Estimated log_10_ of Bayes factors. Numbers in parentheses are standard errors estimated by the standard deviations of 1000 bootstrap samples.

**2C model is best**	${\text{log}}_{10}\frac{p(D|RM)}{p(D|2C)}$	${\text{log}}_{10}\frac{p(D|EM)}{p(D|2C)}$
Fokkens_1a	-4.63(0.46)	-0.98(0.17)
Fokkens_1e	-1.65(0.54)	-1.29(0.35)
Baum_60_2	-1.17(0.59)	-1.46(0.47)
**EM model is best**		
Fokkens_1c	-0.82(0.21)	1.94(0.20)
Baum_57	-0.16(0.52)	58.81(9.82)
Baum_59	53.73(5.94)	77.54(12.58)
**RM and EM models are comparable and best**		
Fokkens_1b	0.73(0.27)	1.02(0.14)
Fokkens_1d	157.71(7.23)	165.48(8.72)
Baum_60_1	2.83(1.02)	2.57(1.32)
**Inconclusive**		
Baum_60_3	3.51(2.73)	1.57(2.19)
Baum_60_4	0.51(3.01)	-0.79(0.64)

**Fig 5:**
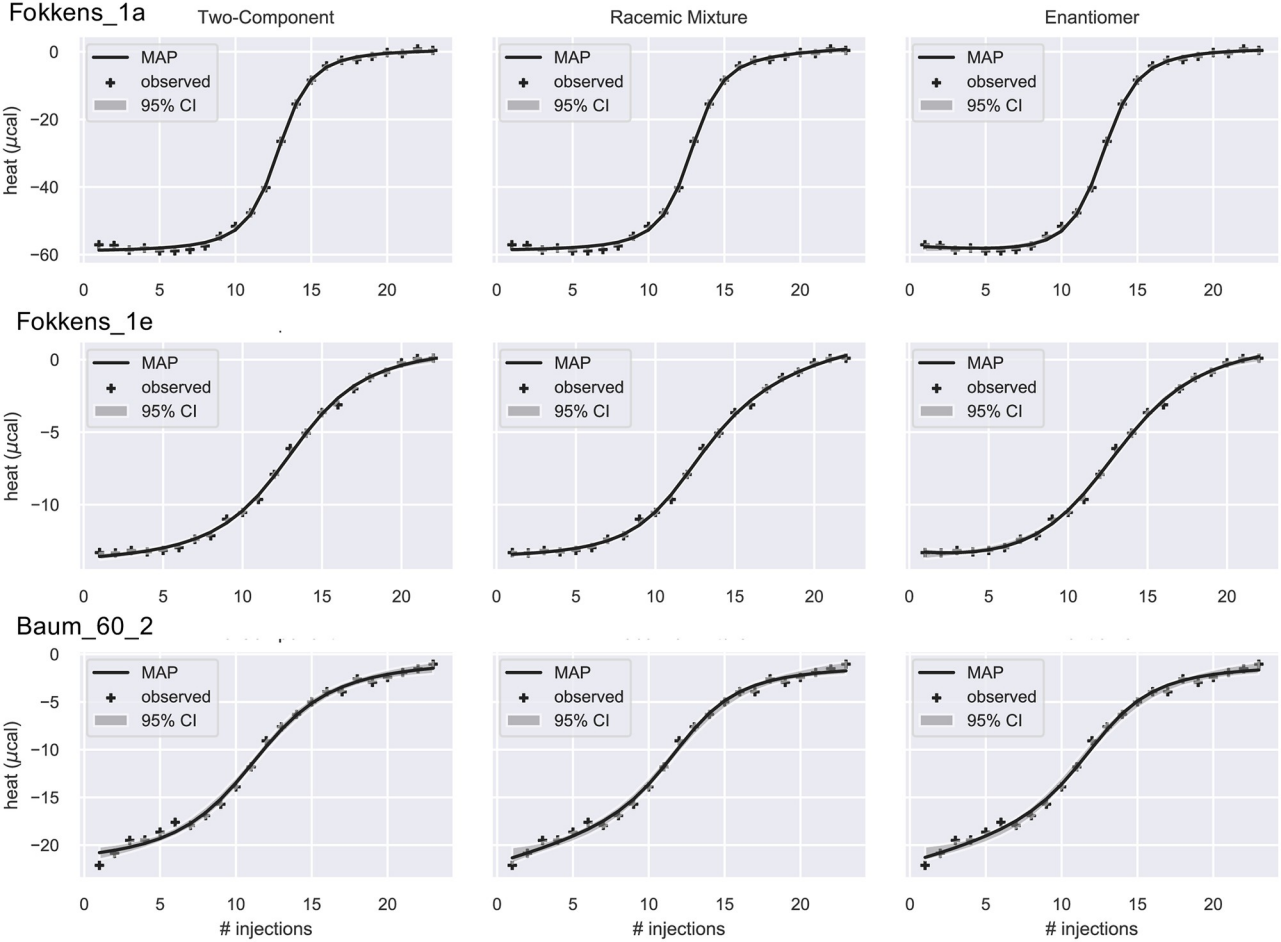
Model fits to integrated heat data. The data were fitted by the 2C (left), RM (middle), and EM (right) models. The solid line is the theoretical heat $q_{n}^{*}\left(\theta_{\text{MAP}}\right)$, where ***θ***_MAP_ is the Maximum a Posteriori estimate of the parameters. The grey band is the 95% confidence interval. Crosses are the observed heat. The three datasets are best fitted by the 2C model according to the estimated Bayes factors (Table 3).

In three titrations, the integrated heats appear sigmoidal and Bayes factors favor the 2C model (Fig 5). Indeed, Fokkens_1a corresponds to titration of trypsin with a single enantiomer, D-Napap (8) [19], and is therefore a two-component binding process. For this dataset, the AIC does not sufficiently penalize model complexity and favors the EM model (S2 Table). The other two datasets, Fokkens_1e and Baum_60_2, correspond to titrations with racemates that have relatively small differences in affinity: 22- [19] and 66-fold [37], respectively. With small differences in affinity, integrated heat curves from the three models are not clearly distinguishable (Fig 5) and thus more complex models are not supported by the data. In addition to small differences in fitting quality, further evidence that the data do not support the RM or EM models is that some parameters (Δ*H*_1_, Δ*H*_2_, Δ*G*_1_, Δ*G*_2_ = Δ*G*_1_ + ΔΔ*G*, or *ρ*) have very broad posteriors (S3 Table) and that the posterior of ΔΔ*G* is inconsistent with reported affinity differences. For Fokkens_1e, the posterior is bimodal the larger peak centered around 0.15 kcal/mol (S3 Table and S4 Appendix), or a multiplicative factor of about $\text{exp}[\frac{0.15}{RT}]=1.3$ times (using the gas constant R and *T* = 298*K* as the temperature), much smaller than the factor determined by independent measurements. In contrast, the posterior of ΔΔ*G* is broad with a peak centered around 4 kcal/mol (S3 Table and S4 Appendix), or 858 fold, much larger than the reported factor.

**Fig 6:**
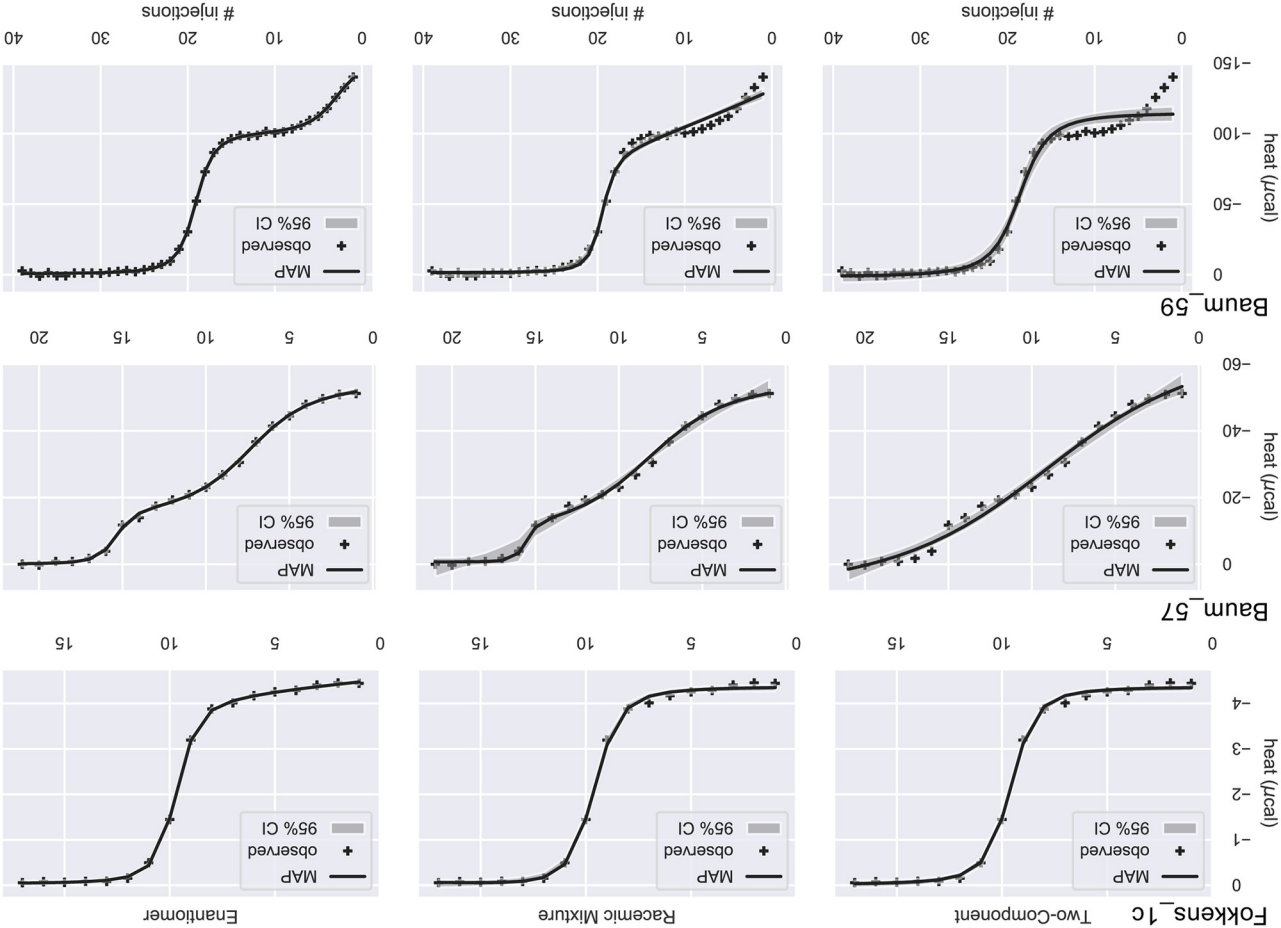
Integrated ITC heat data and their fit. The data were fitted by the 2C (left), RM (middle), and EM (right) models. The solid line is the theoretical heat $q_{n}^{*}\left(\theta_{\text{MAP}}\right)$, where ***θ***_MAP_ is the Maximum a Posteriori estimate of the parameters. The grey band is the 95% confidence interval. Crosses are the observed heat. The three datasets are best fitted by the EM model according to the estimated Bayes factors (Table 3).

In a second group of titrations, the EM model is unambiguously superior according to all model selection criteria (S2 Table). In Baum_57 and Baum_59, the integrated heat data clearly show a two-step binding that cannot be produced by the 2C model (Fig 6). Moreover, the EM model is clearly a better fit than the RC model. Baum_57 corresponds to titration of Thrombin with rac-2. In the EM model, the posterior of *ρ* is peaked near 0.45 (S3 Table and S4 Appendix). Baum_59 corresponds to titration of Trypsin with a mixture of two compounds of different binding affinity, UB_THR_32 and n-pentyl-Benzamindin [37], which is clearly not racemic. The posterior of *ρ* is peaked near 0.15 (S3 Table and S4 Appendix). In Fokkens_1c, the titration of Thrombin with rac-1, the benefit of the EM model is much more subtle, primarily evident at the beginning of the curve (Fig 6). The posterior of *ρ* is broad and peaked near 0.9 (S3 Table and S4 Appendix).

**Fig 7:**
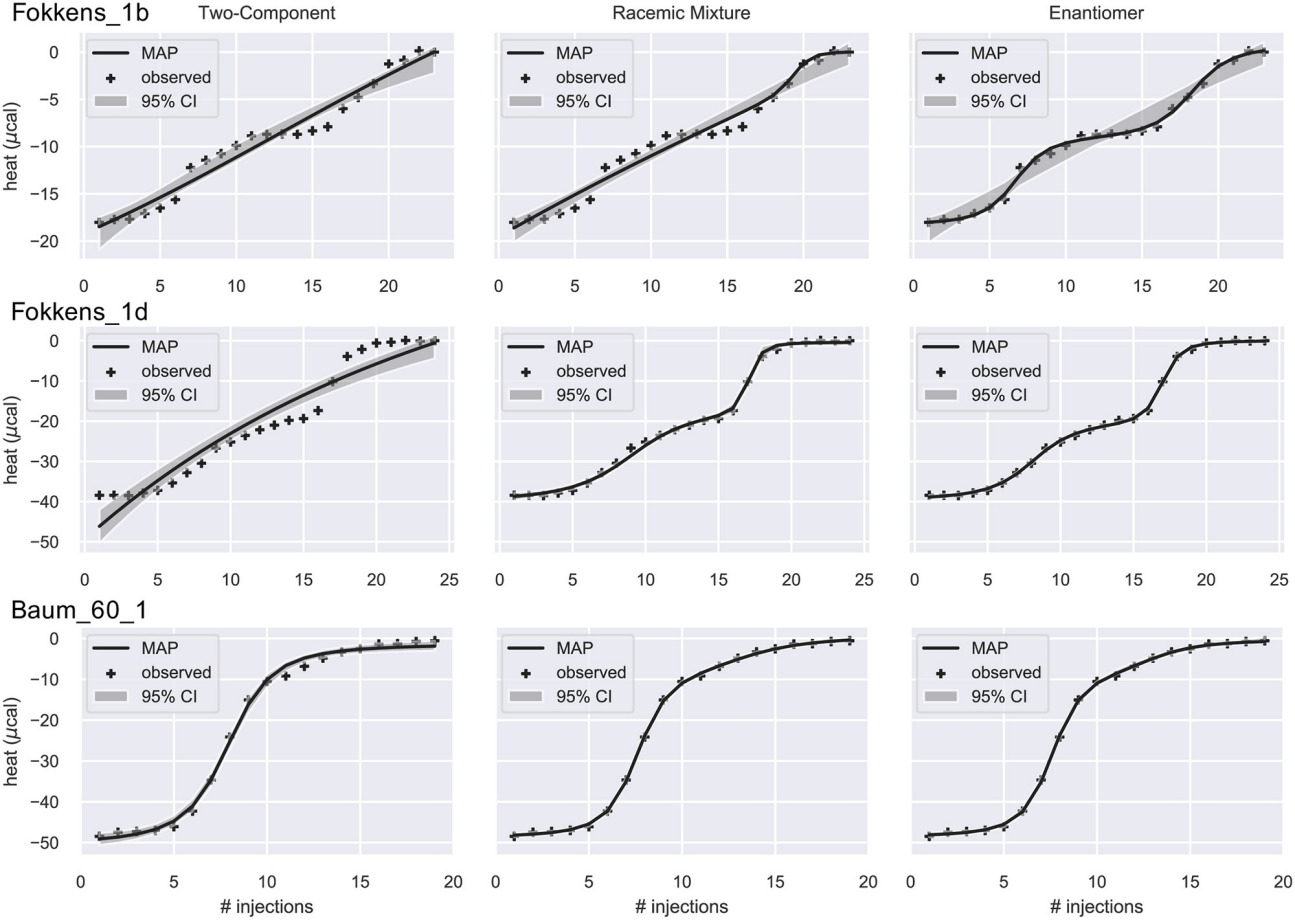
Integrated ITC heat data and their fit. The data were fitted by the 2C (left), RM (middle), and EM (right) models. The solid line is the theoretical heat $q_{n}^{*}\left(\theta_{\text{MAP}}\right)$, where ***θ***_MAP_ is the Maximum a Posteriori estimate of the parameters. The grey band is the 95% confidence interval. Crosses are the observed heat. The three datasets are best fitted by both RM and EM models according to the estimated Bayes factors (Table 3).

In a third group of titrations, both RM and EM models are comparably good and clearly superior to the 2C model (Fig 7). All three datasets involve titration with racemic mixtures. Fokkens_1b corresponds to the titration of Trypsin with rac-Napap (8) [19]. Fokkens_1d is the titration of Thrombin with rac-2 (which is the same system as Baum_57 but with possibly different experimental conditions [19]). Baum_60_1 also corresponds to a racemic mixture titration but a two-step binding is not evident from the heat curve. For Fokkens_1d, the evidence given by Bayes factor (Table 3) for favoring RM and EM models is very strong and all parameters are well-determined. In this case, the posterior of *ρ* is sharply peaked at 0.45, near the RM value of 0.5. Hence any improvements in fit due to the small shift in *ρ* are cancelled by the increased complexity of the model such that differences in Bayes factors are not statistically significant, with ${\text{log}}_{10}\frac{p(D|EM)}{p(D|2C)}=165.48\pm 8.72$ for the RM model and ${\text{log}}_{10}\frac{p(D|RM)}{p(D|2C)}=157.71\pm 7.23$ for the EM model. For the other two datasets, the evidence is not so strong. In particular, both RM and EM models do not show a good fit to Fokkens_1b dataset with rather large 95% CI bands as shown in Fig 7. Some key parameters are also underdetermined for Fokkens_1b and Baum_60_1 (S3 Table).

**Fig 8:**
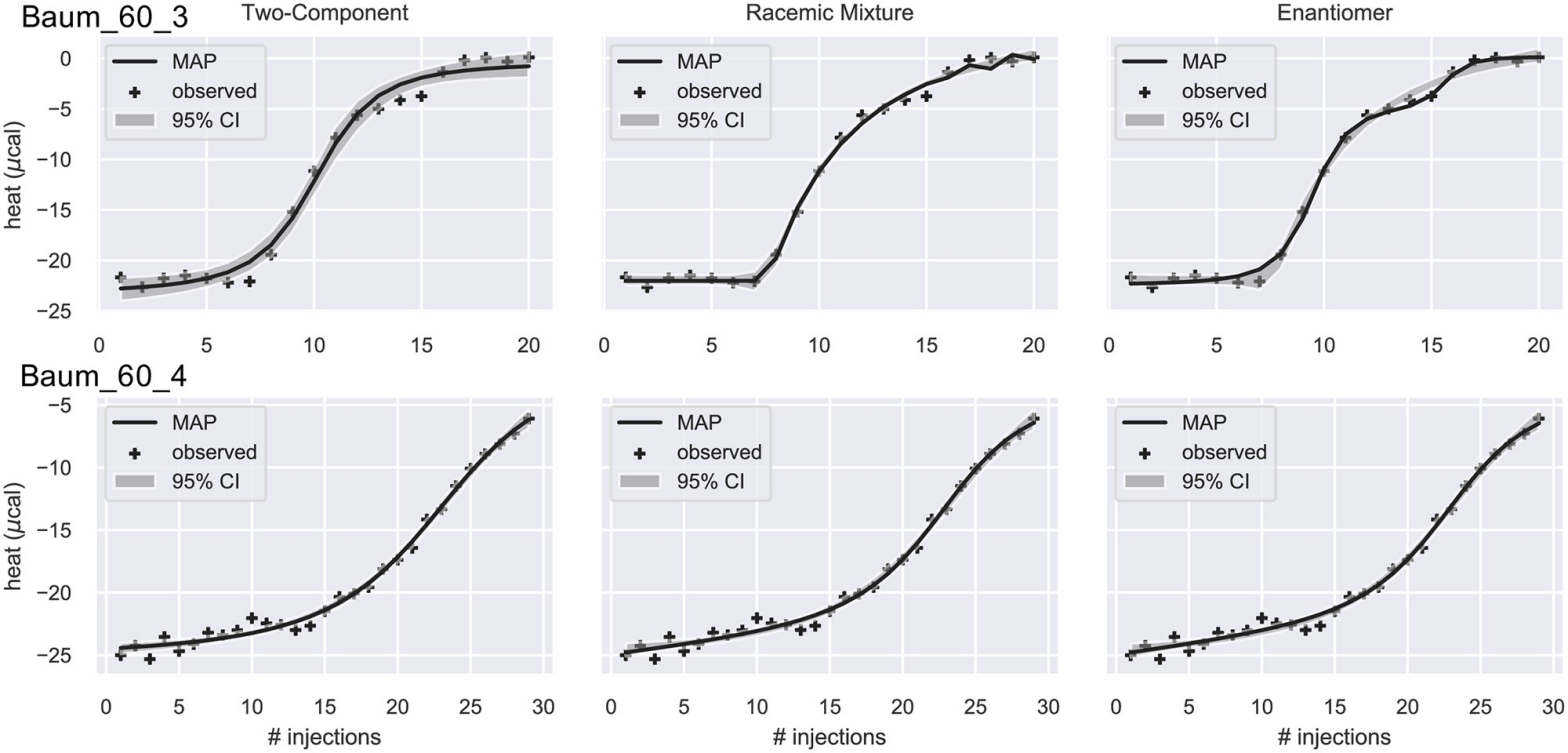
Integrated ITC heat data and their fit. The data were fitted by the 2C (left), RM (middle), and EM (right) models. The solid line is the theoretical heat $q_{n}^{*}\left(\theta_{\text{MAP}}\right)$, where ***θ***_MAP_ is the Maximum a Posteriori estimate of the parameters. The grey band is the 95% confidence interval. Crosses are the observed heat. For these two datasets, the estimated Bayes factors (Table 3) is inconclusive in selecting which model is the best.

In the fourth group (Fig 8), due to the large estimated errors, the estimated Bayes factors are inconclusive about which model is best (Table 3). Probably due to high concentration of the receptor [37], Baum_60_4 does not show saturation.

## Conclusions

We have performed Bayesian regression to infer parameters for thermodynamic binding models from isothermal titration calorimetry measurements in which the titrant is an enantiomeric mixture. When a lognormal prior is used for the concentrations, analyses of simulated experiments provide Bayesian credible intervals that are accurate confidence intervals. Based on our analysis, we are able to determine when the measurements do not provide enough information to precisely determine parameters, leading to broad or multimodal posteriors. We have also introduced a variation of bridge sampling to perform precise estimates of Bayes factors. We find that Bayes factors are superior to other model selection criterion in selecting models that are consistent with prior knowledge about the experiments.

## Supporting information

S1 Appendix Mathematical description of binding models and the Bennett acceptance ratio (BAR) estimator. (PDF)

S2 Appendix Estimated 1D and 2D histograms of key parameters for the 2C model. (PDF)

S3 Appendix Estimated 1D and 2D histograms of key parameters for the RM model. (PDF)

S4 Appendix Estimated 1D and 2D histograms of key parameters for the EM model. (PDF)

S1 Table Summary of systems and experimental protocol. (PDF)

S2 Table Best models according to Bayes factor, Akaike information criterion (AIC) and Bayesian information criterion. (PDF)

S3 Table Estimated Bayesian credible intervals. Binding enthalpy (Δ*H*, Δ*H*_1_, Δ*H*_2_) and free energy (Δ*G*, Δ*G*_1_, ΔΔ*G*) in kcal/mol.
(PDF)

S1 Fig Convergence of percentiles of the Bayesian posterior of the RM model based on the Fokkens_1a dataset. 60,000 samples were drawn from the Bayesian posterior using the NUTS sampler. Six key parameters are shown. Lines correspond to the 5-th (blue circle), 25-th (green square), 50-th (red diamond), 75-th (cyan upward triangle) and 95-th (magenta downward triangle) percentile. The error bars, which are too small to be visible, are standard deviations estimated by 100 bootstrapping samples.
(PDF)

S2 Fig Convergence of percentiles of the Bayesian posterior of the EM model based on the Fokkens_1a dataset and one representative simulation of the EM model. 60,000 samples were drawn from the Bayesian posterior using the NUTS sampler. Six key parameters are shown. Lines correspond to the 5-th (blue circle), 25-th (green square), 50-th (red diamond), 75-th (cyan upward triangle) and 95-th (magenta downward triangle) percentile. The error bars, which are too small to be visible, are standard deviations estimated by 100 bootstrapping samples.
(PDF)

S3 Fig Convergence of Bayes factors for the Fokkens_1b dataset. The Bayes factors were estimated based on 20,000 NUTS samples for the 2C model and 60,000 NUTS samples for the RM and EM models. The error bars are standard deviations estimated by 1000 bootstrapping samples.
(PDF)

S4 Fig Convergence of Bayes factors for the Fokkens_1c dataset. The Bayes factors were estimated based on 20,000 NUTS samples for the 2C model and 60,000 NUTS samples for the RM and EM models. The error bars are standard deviations estimated by 1000 bootstrapping samples.
(PDF)

S5 Fig Convergence of Bayes factors for the Fokkens_1d dataset. The Bayes factors were estimated based on 20,000 NUTS samples for the 2C model and 60,000 NUTS samples for the RM and EM models. The error bars are standard deviations estimated by 1000 bootstrapping samples.
(PDF)

S6 Fig Convergence of Bayes factors for the Fokkens_1e dataset. The Bayes factors were estimated based on 20,000 NUTS samples for the 2C model and 60,000 NUTS samples for the RM and EM models. The error bars are standard deviations estimated by 1000 bootstrapping samples.
(PDF)

S7 Fig Convergence of Bayes factors for the Baum_57 dataset. The Bayes factors were estimated based on 20,000 NUTS samples for the 2C model and 60,000 NUTS samples for the RM and EM models. The error bars are standard deviations estimated by 1000 bootstrapping samples.
(PDF)

S8 Fig Convergence of Bayes factors for the Baum_59 dataset. The Bayes factors were estimated based on 20,000 NUTS samples for the 2C model and 60,000 NUTS samples for the RM and EM models. The error bars are standard deviations estimated by 1000 bootstrapping samples.
(PDF)

S9 Fig Convergence of Bayes factors for the Baum_60_1 dataset. The Bayes factors were estimated based on 20,000 NUTS samples for the 2C model and 60,000 NUTS samples for the RM and EM models. The error bars are standard deviations estimated by 1000 bootstrapping samples.
(PDF)

S10 Fig Convergence of Bayes factors for the Baum_60_2 dataset. The Bayes factors were estimated based on 20,000 NUTS samples for the 2C model and 60,000 NUTS samples for the RM and EM models. The error bars are standard deviations estimated by 1000 bootstrapping samples.
(PDF)

S11 Fig Convergence of Bayes factors for the Baum_60_3 dataset. The Bayes factors were estimated based on 20,000 NUTS samples for the 2C model and 60,000 NUTS samples for the RM and EM models. The error bars are standard deviations estimated by 1000 bootstrapping samples.
(PDF)

S12 Fig Convergence of Bayes factors for the Baum_60_4 dataset. The Bayes factors were estimated based on 20,000 NUTS samples for the 2C model and 60,000 NUTS samples for the RM and EM models. The error bars are standard deviations estimated by 1000 bootstrapping samples.
(PDF)
