# Supplementary material for: Bayesian regression and model selection for isothermal titration calorimetry with enantiomeric mixtures
Source: PLoS One. 2022 Sep 29;17(9):e0273656. doi: 10.1371/journal.pone.0273656 (PMC9521810; doi:10.1371/journal.pone.0273656)
Supplement: S1 Appendix — (PDF) [file pone.0273656.s001.pdf]

Appendix

Mathematical expressions for binding models

Two-component (2C) binding model

The two-component model is based on a reversible association between a ligand L and a receptor R to form a complex RL,

$$R + L \overset{K_a}{\rightleftharpoons} RL, \tag{A.1}$$

where the *association constant* K_a or the binding free energy ΔG is related to concentrations $[X]$ of the microscopic species at equilibrium by,

$$K_a \equiv \exp(-\beta \Delta G) = \frac{[RL]c^\theta}{[R][L]}. \tag{A.2}$$

where $c^\theta = 1$ M is the standard concentration. While the ΔG defined here is the standard binding free energy, for notational simplicity we omit the superscript $^\theta$ in our labels for thermodynamic parameters.

With each injection, three effects will contribute to the true quantity of heat q_i^* liberated due to injection i: (1) the association of R with L, (2) the dilution of ligand and buffer into the protein solution (as most solutions are nonideal), and (3) the mechanical heat produced by the injection and stirring. We subsume the latter two components into a single term ΔH_0, and write,

$$q_i^* = \Delta H * V_0 * ([RL]_i - d_i * [RL]_{i-1}) + \Delta H_0, \tag{A.3}$$

where ΔH is the enthalpy change associated with binding, $[RL]_i$ is the complex equilibrium concentration after injection i, V_0 is the cell volume, and d_i is the dilution factor after an injection with volume v_i, defined as,

$$d_i = 1 - (v_i/V_0). \tag{A.4}$$

In what follows, we will express the complex equilibrium concentration after injection i, $[RL]_i$, in terms of K_a, the cell volume V_0, the initial concentration of the receptor $[R]_0$, and syringe concentration of the ligand $[L]_s$.

The total quantity (number of moles) of receptor R_i and ligand L_i in the cell after injection i is given by,

$$R_i = V_0 * [R]_0 * d_{\mathrm{cum},i}, \tag{A.5}$$

$$L_i = V_0 * [L]_s * (1 - d_{\mathrm{cum},i}), \tag{A.6}$$

where $d_{\mathrm{cum},i}$ is the cumulative dilution factor given by,

$$d_{\mathrm{cum},i} = \prod_i 1 - (v_i/V_0). \quad \text{(A.7)}$$

This model accounts for perfusion of receptor and ligand from the cell at a constant cell volume while assuming the mixing after injection is instantaneous, as in [1].

Conservation of mass gives us the constraints,

$$R_i = V_0 * ([R]_i + [RL]_i), \quad \text{(A.8)}$$

$$L_i = V_0 * ([L]_i + [RL]_i). \quad \text{(A.9)}$$

Combining Eqs. A.2 and A.9 gives,

$$[R]_i = \frac{[RL]_i}{K_a \frac{L_i}{V_0} - K_a [RL]_i}. \quad \text{(A.10)}$$

Substituting Eq. A.10 into Eq. A.8 yields a quadratic equation in the complex equilibrium concentration $[RL]_i$:

$$[RL]_i^2 - \left(\frac{R_i}{V_0} + \frac{1}{K_a} + \frac{L_i}{V_0} \right) [RL]_i + \frac{R_i \cdot L_i}{V_0^2} = 0, \quad \text{(A.11)}$$

where the only solution that satisfies $0 \leq [RL]_i \leq \min\{[R], [L]_i\}$ is,

$$[RL]_i = \frac{1}{2V_0} \left\{ \left(R_i + L_i + \frac{V_0}{K_a} \right) - \left[\left(R_i + L_i + \frac{V_0}{K_a} \right)^2 - 4R_i L_i \right]^{\frac{1}{2}} \right\}. \quad \text{(A.12)}$$

Model for competitive binding of two ligands to a receptor

When titrating a receptor R with a mixture of ligands $L^{(1)}$ and $L^{(2)}$, two equilibrium association processes occur:

$$R + L^{(1)} \overset{K_{a1}}{\rightleftharpoons} RL^{(1)}, \quad R + L^{(2)} \overset{K_{a2}}{\rightleftharpoons} RL^{(2)}, \quad \text{(A.13)}$$

where the association constants are given by,

$$K_{a1} \equiv \frac{1}{c^\theta} \exp(-\beta \Delta G_1) = \frac{[RL^{(1)}]}{[R][L^{(1)}]}, \quad \text{(A.14)}$$

$$K_{a2} \equiv \frac{1}{c^\theta} \exp(-\beta \Delta G_2) = \frac{[RL^{(2)}]}{[R][L^{(2)}]}. \quad \text{(A.15)}$$

Similarly to Eq. A.3, the heat produced after injection i is given by,

$$\begin{aligned} q_i^* &= \Delta H_1 * V_0 * \left([RL^{(1)}]_i - d_i * [RL^{(1)}]_{i-1} \right) \\ &+ \Delta H_2 * V_0 * \left([RL^{(2)}]_i - d_i * [RL^{(2)}]_{i-1} \right) + \Delta H_0, \end{aligned} \quad \text{(A.16)}$$

where ΔH_1 and ΔH_2 are enthalpy changes associated with binding of ligands $L^{(1)}$ and $L^{(2)}$, respectively.

The total quantities of receptor and ligands in the cell after injection i are,

$$R_i = V_0 * [R]_0 * d_{\text{cum},i}, \tag{A.17}$$

$$L_i^{(1)} = V_0 * \rho * [L]_s * (1 - d_{\text{cum},i}), \tag{A.18}$$

$$L_i^{(2)} = V_0 * (1 - \rho) * [L]_s * (1 - d_{\text{cum},i}), \tag{A.19}$$

where $[R]_0$ is the initial concentration of the cell and $[L]_s$ is the concentration of the syringe. ρ is the proportion of ligand $L^{(1)}$. ρ is set to 0.5 in the RM model and considered a free parameter varying from 0 to 1 in the Enantiomer model.

Now we will express the complex concentrations $[RL^{(1)}]_i$ and $[RL^{(2)}]_i$ in terms of K_{a1}, K_{a2}, V_0, $[R]_0$ and $[L]_s$ by following a previous derivation [2].

By the conservation of mass, we have,

$$R_i = V_0 * ([R]_i + [RL^{(1)}]_i + [RL^{(2)}]_i), \tag{A.20}$$

$$L_i^{(1)} = V_0 * ([L^{(1)}]_i + [RL^{(1)}]_i), \tag{A.21}$$

$$L_i^{(2)} = V_0 * ([L^{(2)}]_i + [RL^{(2)}]_i). \tag{A.22}$$

Combining Eqs. A.15 into A.21 and A.22, we obtain,

$$[RL^{(1)}]_i = \frac{[R]_i L_i^{(1)}}{\frac{V_0}{K_{a1}} + V_0[R]_i}, \quad [RL^{(2)}]_i = \frac{[R]_i L_i^{(2)}}{\frac{V_0}{K_{a2}} + V_0[R]_i}. \tag{A.23}$$

Substituting Eq. A.23 into A.20 and rearranging leads to,

$$[R]_i^3 + a * [R]_i^2 + b * [R]_i + c = 0, \tag{A.24}$$

where

$$a = \frac{1}{K_{a1}} + \frac{1}{K_{a2}} + \frac{1}{V_0}\left(L_i^{(1)} + L_i^{(2)} - R_i\right), \tag{A.25}$$

$$b = \frac{1}{V_0 K_{a2}}\left(L_i^{(1)} - R_i\right) + \frac{1}{V_0 K_{a1}}\left(L_i^{(2)} - R_i\right) + \frac{1}{K_{a1}K_{a2}} \tag{A.26}$$

$$c = -\frac{R_i}{V_0 K_{a1} K_{a2}} \tag{A.27}$$

Eq. A.24 has three real roots but only one of them is physically meaningful [2]. The meaningful root is,

$$[R]_i = -\frac{a}{3} + \frac{2}{3}\sqrt{a^2 - 3b}\cos\frac{\theta}{3}, \tag{A.28}$$

where

$$\theta = \arccos \frac{-2a^3 + 9ab - 27c}{2\sqrt{(a^2 - 3b)^3}}. \tag{A.29}$$

Substituting Eq. A.28 into Eq. A.23, we obtain expressions for $[RL^{(1)}]_i$ and $[RL^{(2)}]_i$ as,

$$[RL^{(1)}]_i = \frac{L_i^{(1)} \left[2\sqrt{(a^2 - 3b)} \cos \frac{\theta}{3} - a\right]}{3\frac{V_0}{K_{a1}} + V_0 \left[2\sqrt{(a^2 - 3b)} \cos \frac{\theta}{3} - a\right]}, \tag{A.30}$$

$$[RL^{(2)}]_i = \frac{L_i^{(2)} \left[2\sqrt{(a^2 - 3b)} \cos \frac{\theta}{3} - a\right]}{3\frac{V_0}{K_{a2}} + V_0 \left[2\sqrt{(a^2 - 3b)} \cos \frac{\theta}{3} - a\right]}. \tag{A.31}$$

Bennett acceptance ratio (BAR) estimator

The BAR estimator [3] is commonly used in molecular simulation to estimate the free energy difference between a pair of thermodynamic states. The free energy difference between an initial state i and a final state f is defined as,

$$\Delta F \equiv F_f - F_i = -\ln \frac{\int e^{-u_f(x)} dx}{\int e^{-u_i(x)} dx}, \tag{A.32}$$

where u_i and u_f are dimensionless potential energy functions of states i and f, respectively. x is the coordinate of the system in a high-dimensional configuration space. Integrals are taken over the entire configuration space. Unnormalized probability densities are related to potential energies as,

$$p_i(x) \equiv e^{-u_i(x)}, \quad p_f(x) \equiv e^{-u_f(x)}. \tag{A.33}$$

In this formulation, the free energy difference (Eq. A.32) is actually equal to the logarithm of the ratio of the two normalization constants.

The derivation of the BAR estimator [3] starts by rewriting Eq. A.32 as ratio of expectation values,

$$\Delta F = \frac{\left\langle \alpha(x) e^{-u_i(x)} \right\rangle_f}{\left\langle \alpha(x) e^{-u_f(x)} \right\rangle_i}, \quad \forall \alpha(x) > 0, \forall x, \tag{A.34}$$

where $\alpha(x)$ is an arbitrary positive function of x. $< \ldots >_i$ and $< \ldots >_f$ denote expectations with respect to the densities $p_i(x)$ and $p_f(x)$, respectively. $\alpha(x)$ is chosen by minimizing the asymptotic variance of the free energy difference. This results in an implicit function for ΔF which is found by solving,

$$\sum_{\alpha=1}^{N_i} \frac{1}{1 + \exp\left[\ln \frac{N_i}{N_f} + \Delta u(x_\alpha^i) - \Delta F\right]} + \sum_{\beta=1}^{N_f} \frac{1}{1 + \exp\left[\ln \frac{N_f}{N_i} - \Delta u(x_\beta^f) + \Delta F\right]} = 0, \tag{A.35}$$

where $\Delta u(x) = u_f(x) - u_i(x)$. x^i is a sample drawn from p_i and x^f drawn from p_f.

When applied to nested statistical models as described in the main text, the potential energy functions for the initial and final states, u_1 and u_2 are given by,

$$u_1(\boldsymbol{\theta}_1, \boldsymbol{\gamma}) \equiv -\ln p_1(\boldsymbol{\theta}_1) - \ln f(\boldsymbol{\gamma}), \tag{A.36}$$

and,

$$u_2(\boldsymbol{\theta}_1, \boldsymbol{\gamma}) \equiv -\ln p_2(\boldsymbol{\theta}_1, \boldsymbol{\gamma}). \tag{A.37}$$

References

[1] Tellinghuisen J. (2007), Calibration in isothermal titration calorimetry: Heat and cell volume from heat of dilution of NaCl(aq), Analytical Biochemistry 360(1):47–55.

[2] Wang, Z.-X. (1995), An exact mathematical expression for describing competitive binding of two different ligands to a protein molecule, FEBS Letters 360, 111-114.

[3] Bennett, C. H (1976). Efficient Estimation of Free Energy Differences from Monte Carlo Data, Journal of Computational Physics 22, 245–268.
