# Supplementary material for: Bayesian regression and model selection for isothermal titration calorimetry with enantiomeric mixtures
Source: PLoS One. 2022 Sep 29;17(9):e0273656. doi: 10.1371/journal.pone.0273656 (PMC9521810; doi:10.1371/journal.pone.0273656)
Supplement: S2 Appendix — (PDF) [file pone.0273656.s002.pdf]

Two-Component model

Baum_57

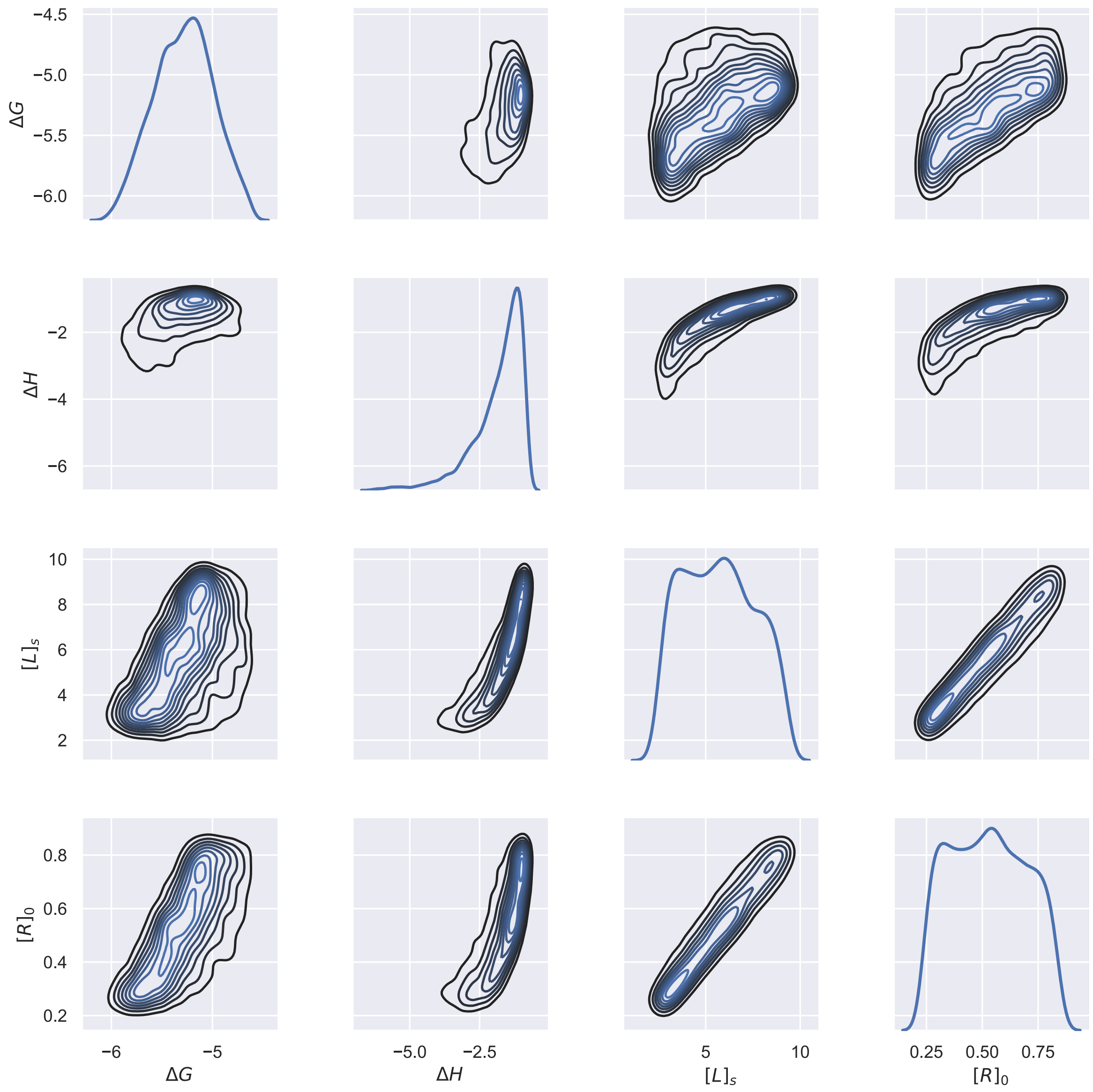

Baum_59

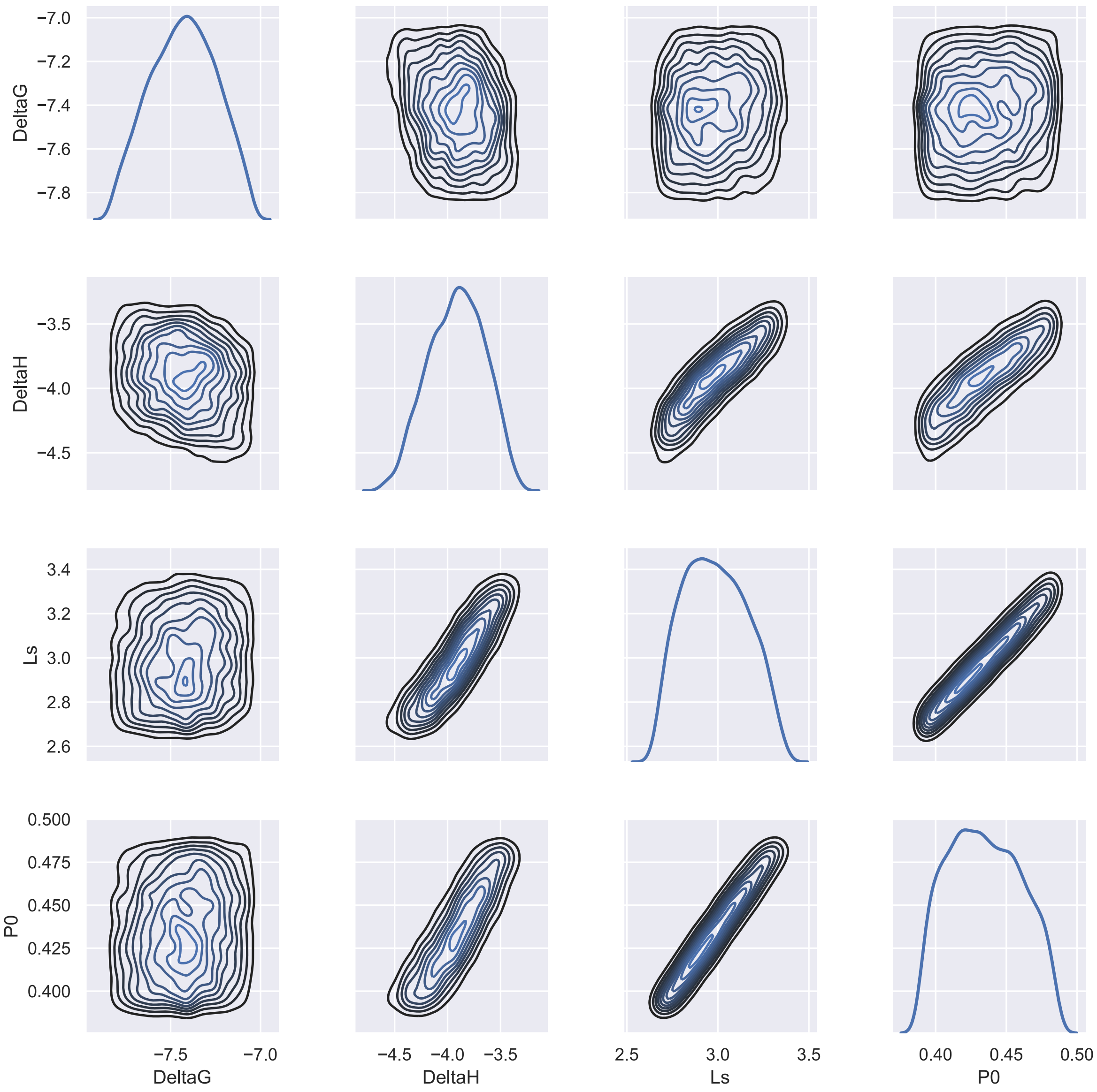

DeltaG
DeltaH
Ls
P0
−7.0
−7.2
−7.4
−7.6
−7.8
−3.5
−4.0
−4.5
3.4
3.2
3.0
2.8
2.6
0.500
0.475
0.450
0.425
0.400
−7.5
−7.0
−4.5
−4.0
−3.5
2.5
3.0
3.5
0.40
0.45
0.50

Baum_60_1

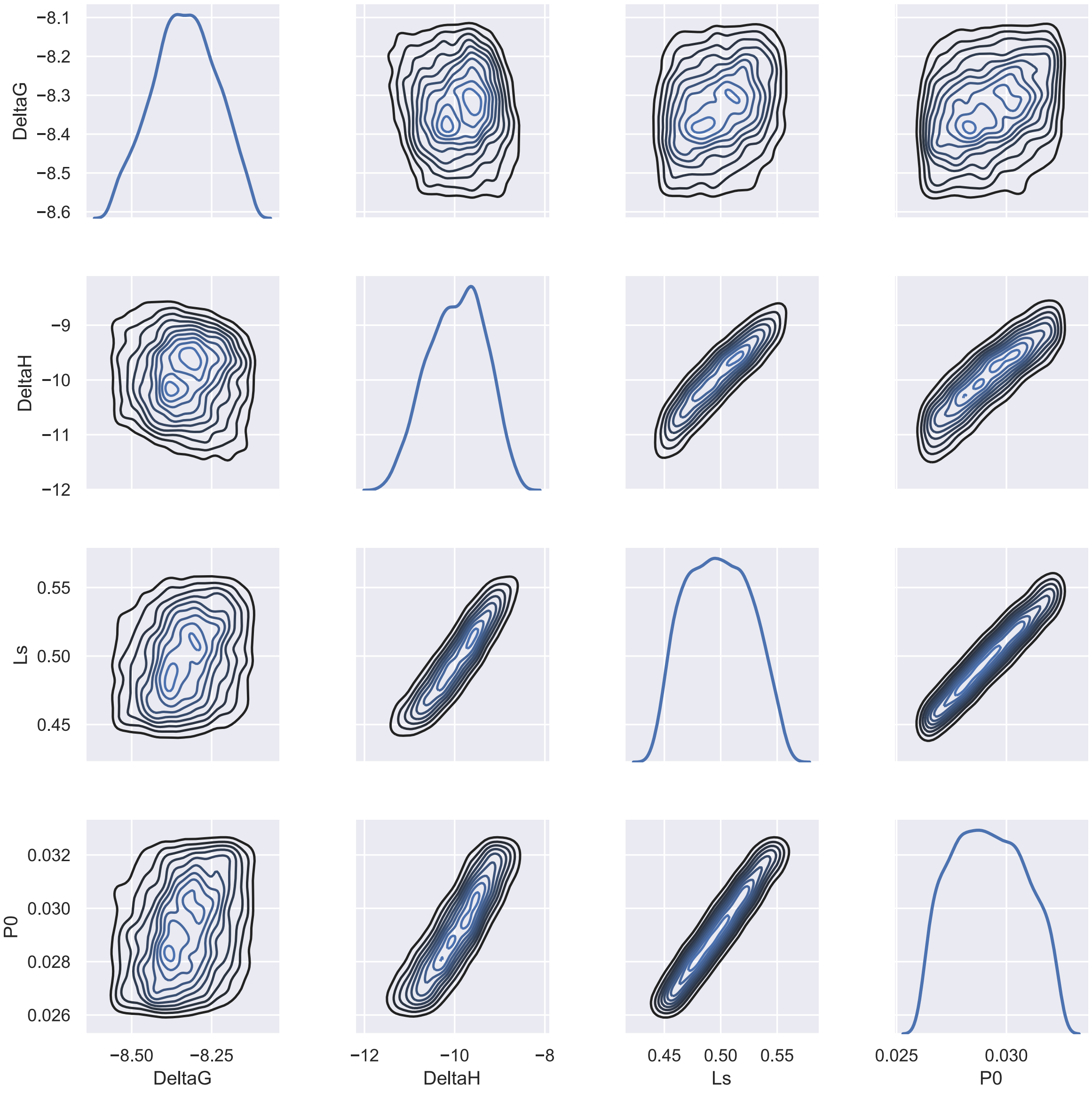

DeltaG
DeltaH
Ls
P0
−8.1
−8.2
−8.3
−8.4
−8.5
−8.6
−9
−10
−11
−12
0.55
0.50
0.45
0.032
0.030
0.028
0.026
−8.50
−8.25
−12
−10
−8
0.45
0.50
0.55
0.025
0.030
DeltaG
DeltaH
Ls
P0

Baum_60_2

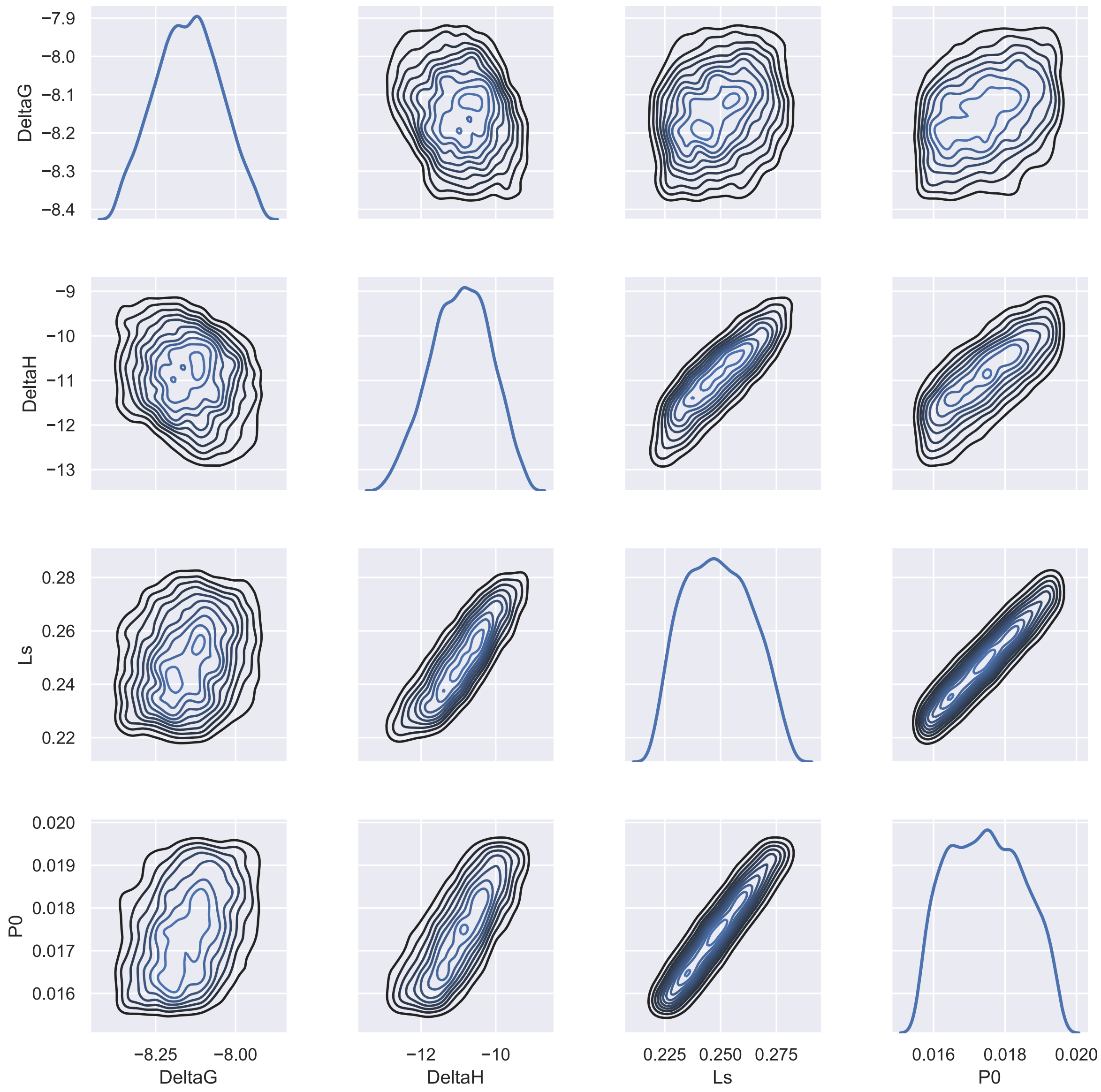

DeltaG
DeltaH
Ls
P0
−7.9
−8.0
−8.1
−8.2
−8.3
−8.4
−9
−10
−11
−12
−13
0.28
0.26
0.24
0.22
0.020
0.019
0.018
0.017
0.016
−8.25
−8.00
−12
−10
0.225
0.250
0.275
0.016
0.018
0.020
DeltaG
DeltaH
Ls
P0

Baum_60_3

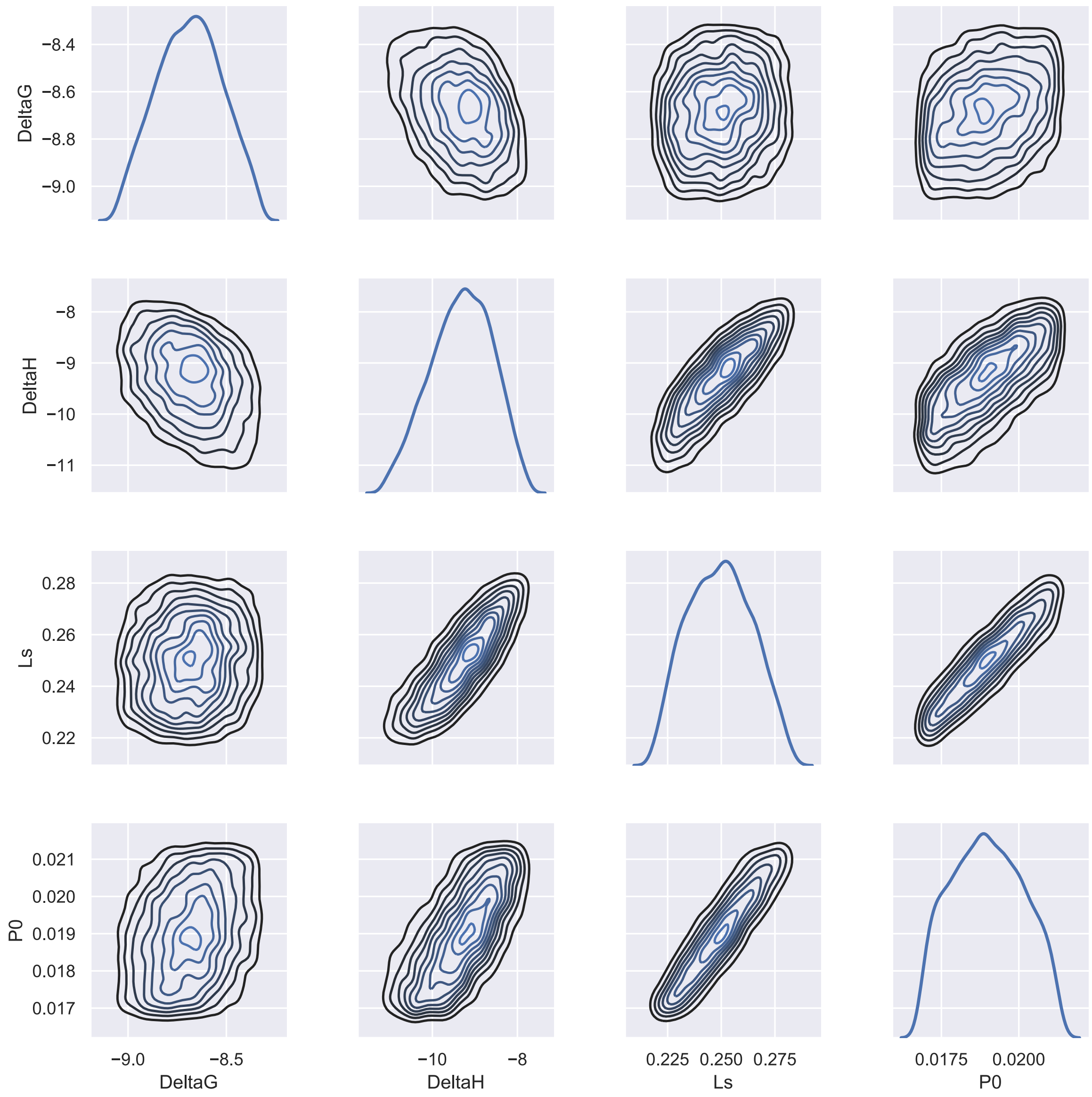
DeltaG
-8.4
-8.6
-8.8
-9.0
DeltaH
-8
-9
-10
-11
Ls
0.28
0.26
0.24
0.22
P0
0.021
0.020
0.019
0.018
0.017
-9.0
-8.5
DeltaG
-10
-8
DeltaH
0.225
0.250
0.275
Ls
0.0175
0.0200
P0

Baum_60_4

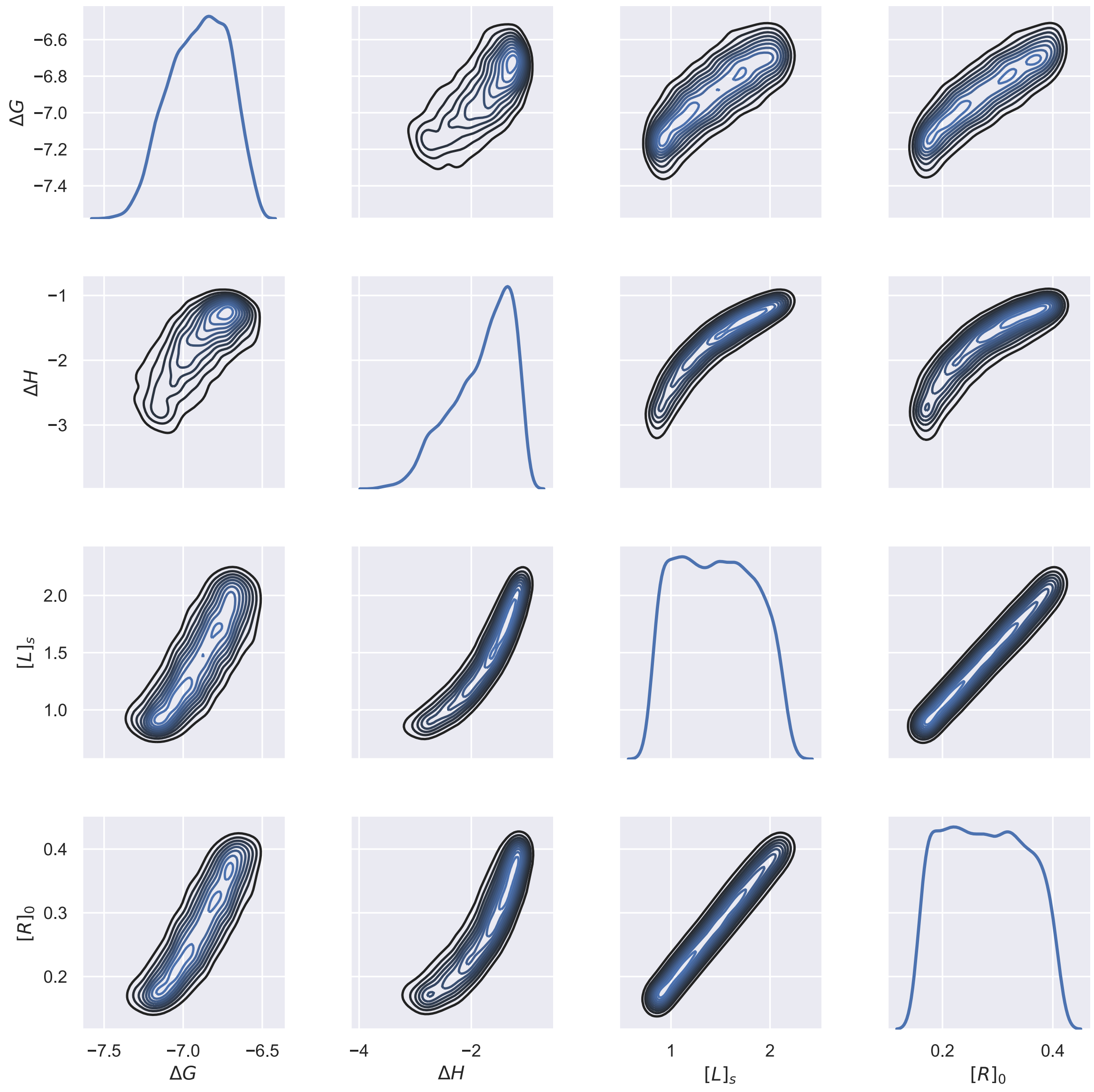

−6.6
−6.8
−7.0
−7.2
−7.4
ΔG
−1
−2
−3
ΔH
2.0
1.5
1.0
$[L]_s$
0.4
0.3
0.2
$[R]_0$
−7.5
−7.0
−6.5
ΔG
−4
−2
ΔH
1
2
$[L]_s$
0.2
0.4
$[R]_0$

Fokkens_1_a

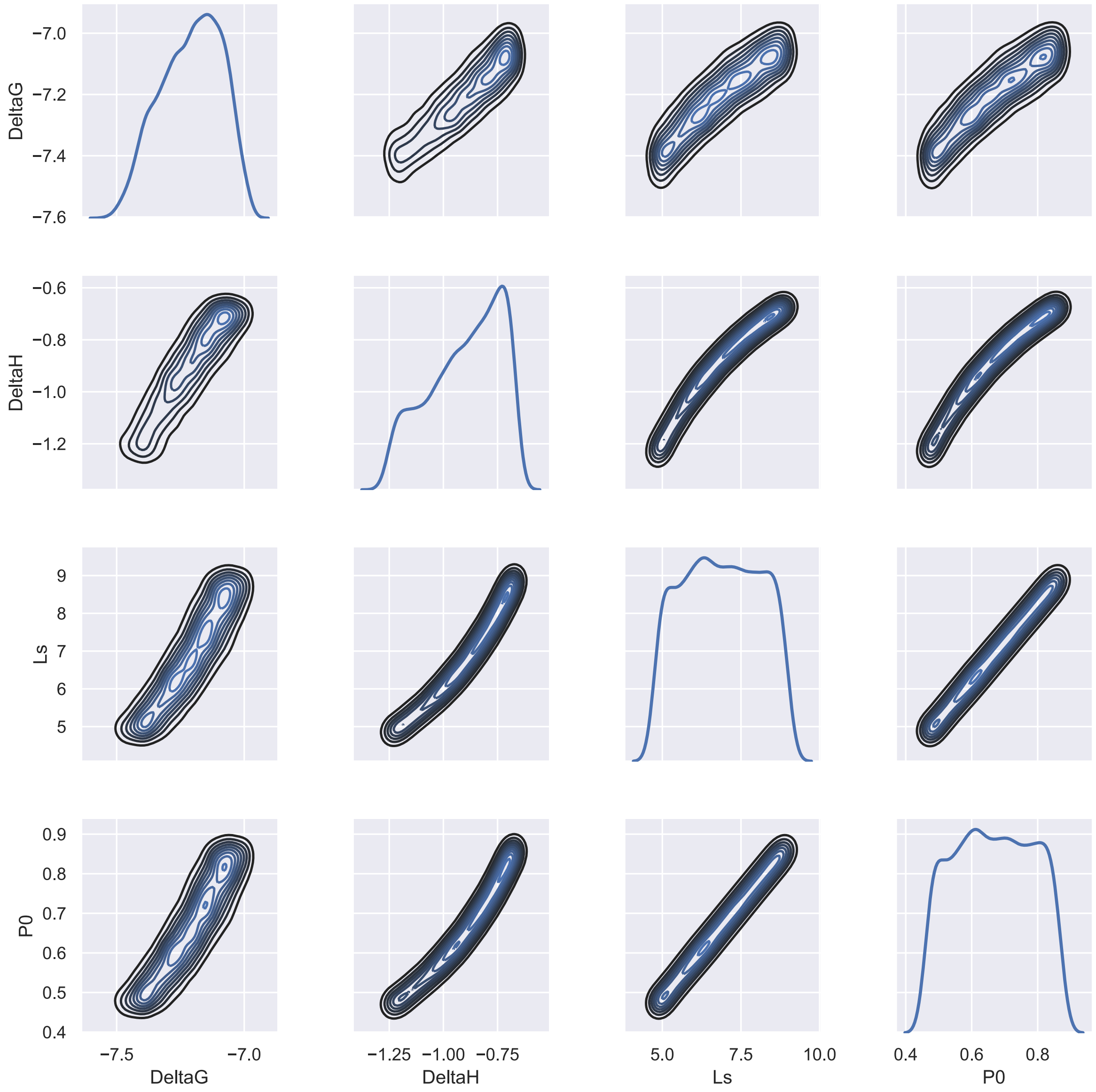
DeltaG
DeltaH
Ls
P0
DeltaG
DeltaH
Ls
P0
−7.0
−7.2
−7.4
−7.6
−0.6
−0.8
−1.0
−1.2
9
8
7
6
5
0.9
0.8
0.7
0.6
0.5
0.4
−7.5
−7.0
−1.25
−1.00
−0.75
5.0
7.5
10.0
0.4
0.6
0.8

Fokkens_1_b

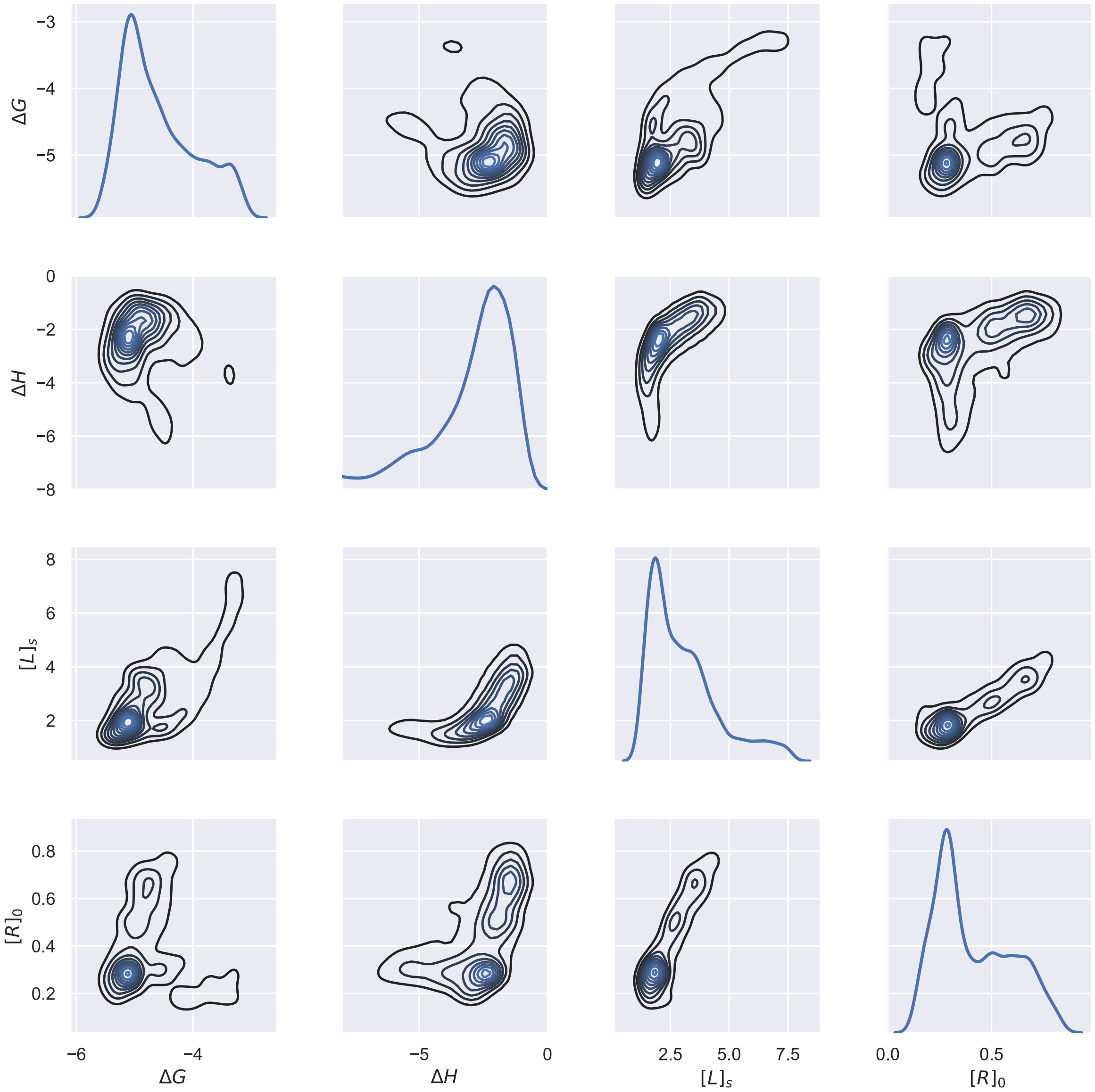
ΔG
ΔH
[L]s
[R]0
−3
−4
−5
0
−2
−4
−6
−8
8
6
4
2
0.8
0.6
0.4
0.2
−6
−4
−5
0
2.5
5.0
7.5
0.0
0.5
ΔG
ΔH
[L]s
[R]0

Fokkens_1_c

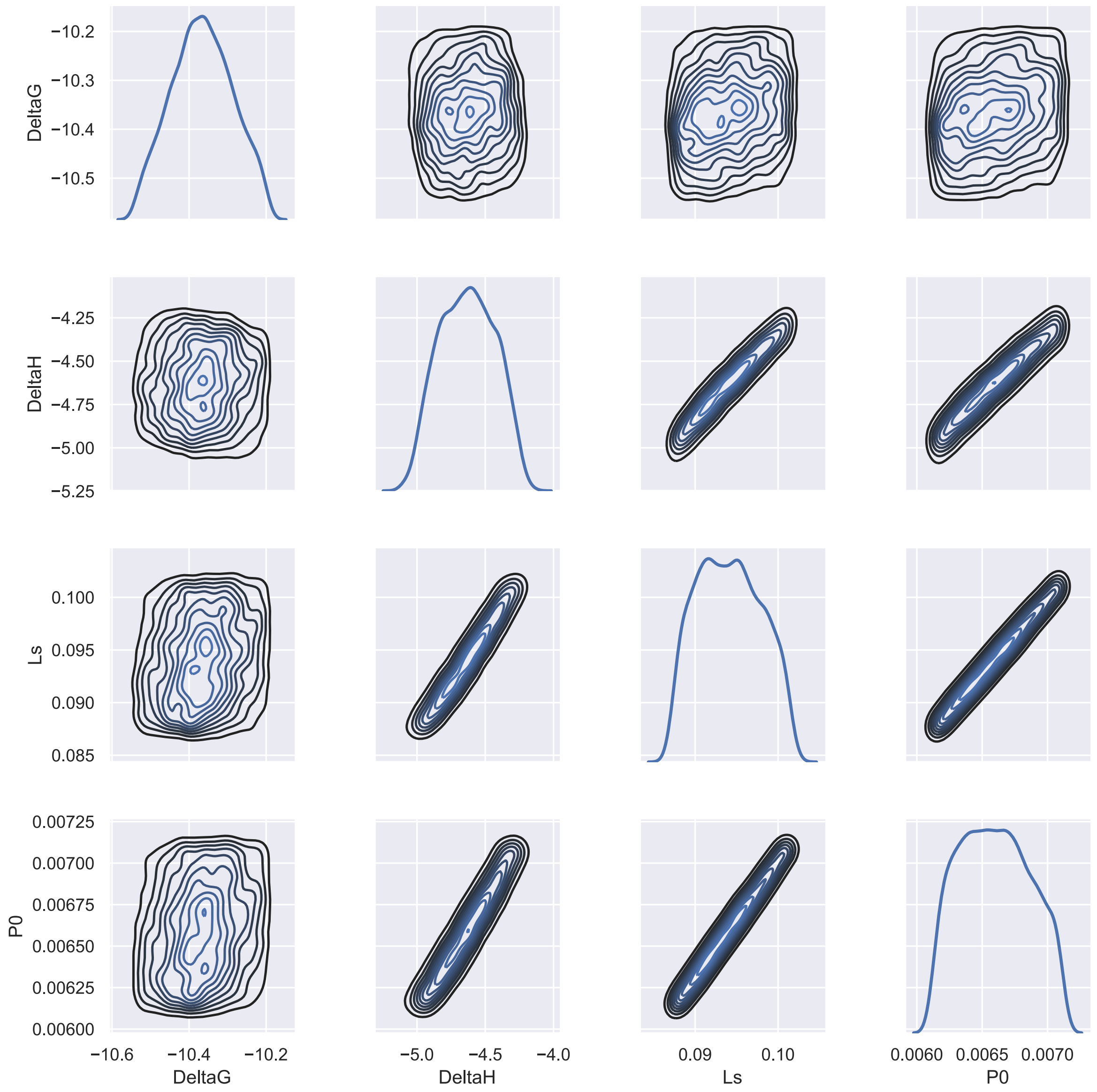
DeltaG
DeltaH
Ls
P0
−10.2
−10.3
−10.4
−10.5
−4.25
−4.50
−4.75
−5.00
−5.25
0.100
0.095
0.090
0.085
0.00725
0.00700
0.00675
0.00650
0.00625
0.00600
−10.6
−10.4
−10.2
−5.0
−4.5
−4.0
0.09
0.10
0.0060
0.0065
0.0070

Fokkens_1_d

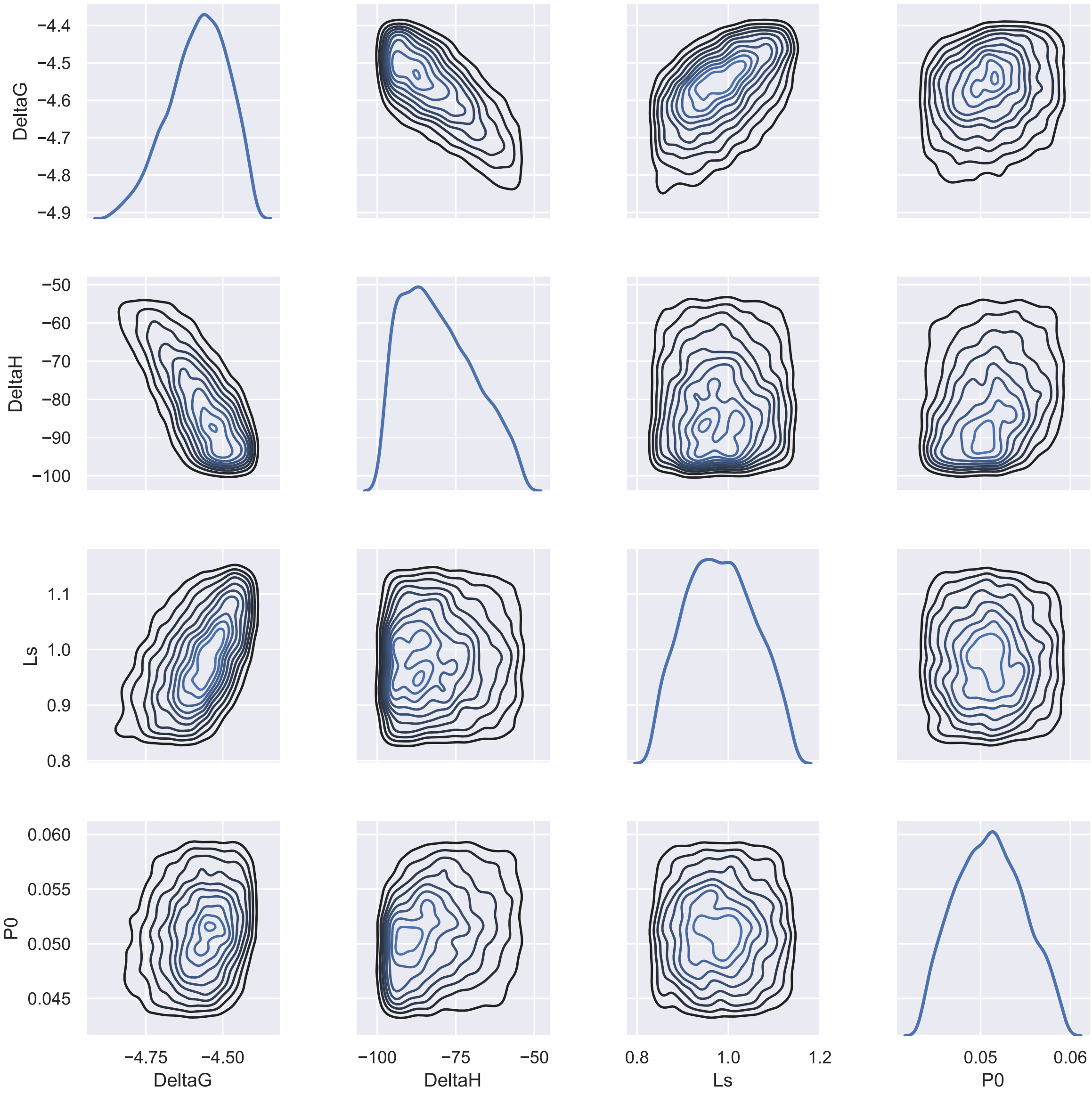

DeltaG
DeltaH
Ls
P0
−4.4
−4.5
−4.6
−4.7
−4.8
−4.9
−50
−60
−70
−80
−90
−100
1.1
1.0
0.9
0.8
0.060
0.055
0.050
0.045
−4.75
−4.50
−100
−75
−50
0.8
1.0
1.2
0.05
0.06

Fokkens_1_e

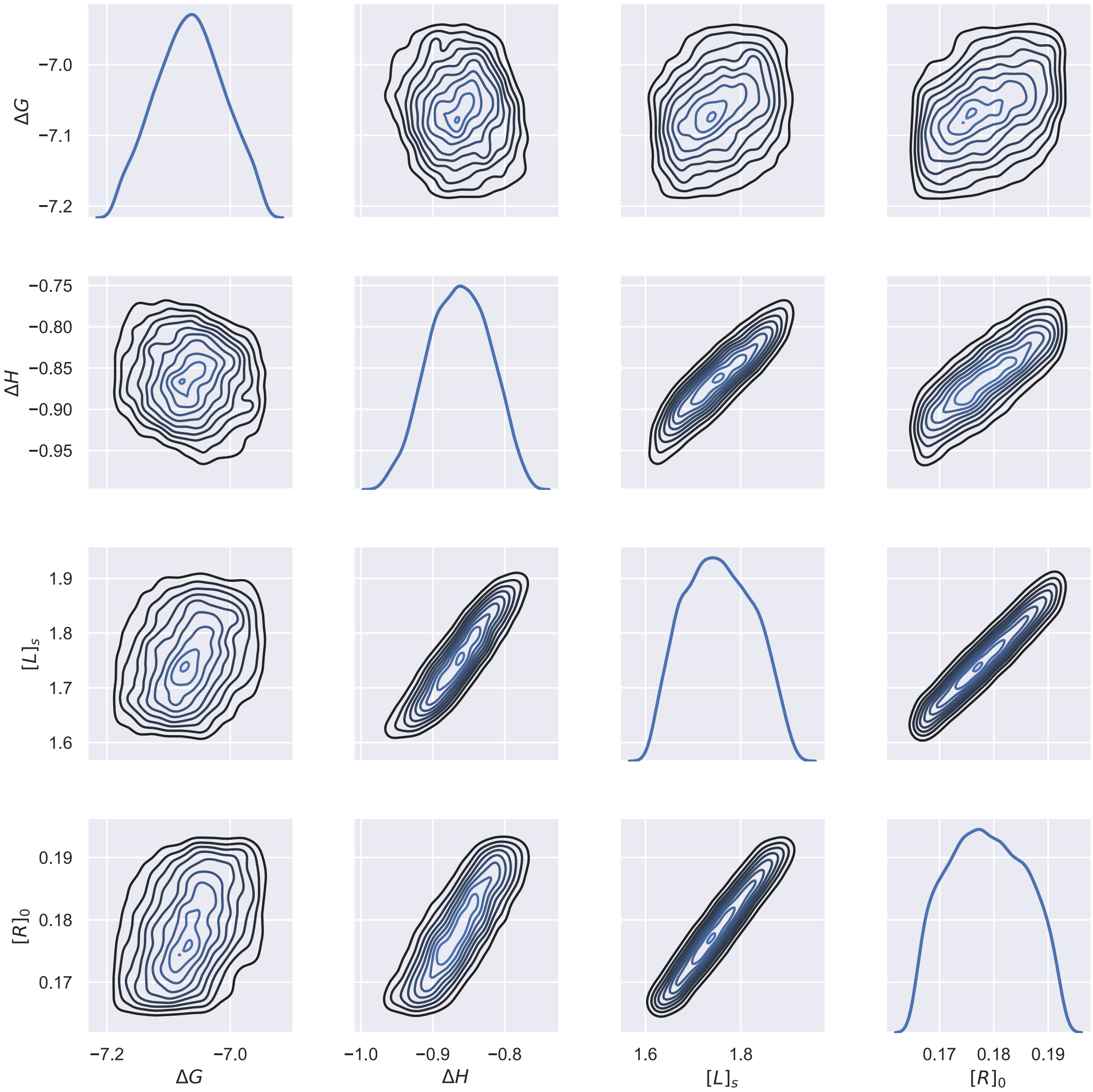

−7.0
−7.1
−7.2
ΔG
−0.75
−0.80
−0.85
−0.90
−0.95
ΔH
1.9
1.8
1.7
1.6
$[L]_s$
0.19
0.18
0.17
$[R]_0$
−7.2
−7.0
ΔG
−1.0
−0.9
−0.8
ΔH
1.6
1.8
$[L]_s$
0.17
0.18
0.19
$[R]_0$
