# Supplementary material for: Bayesian regression and model selection for isothermal titration calorimetry with enantiomeric mixtures
Source: PLoS One. 2022 Sep 29;17(9):e0273656. doi: 10.1371/journal.pone.0273656 (PMC9521810; doi:10.1371/journal.pone.0273656)

Racemic mixture model

Baum_57

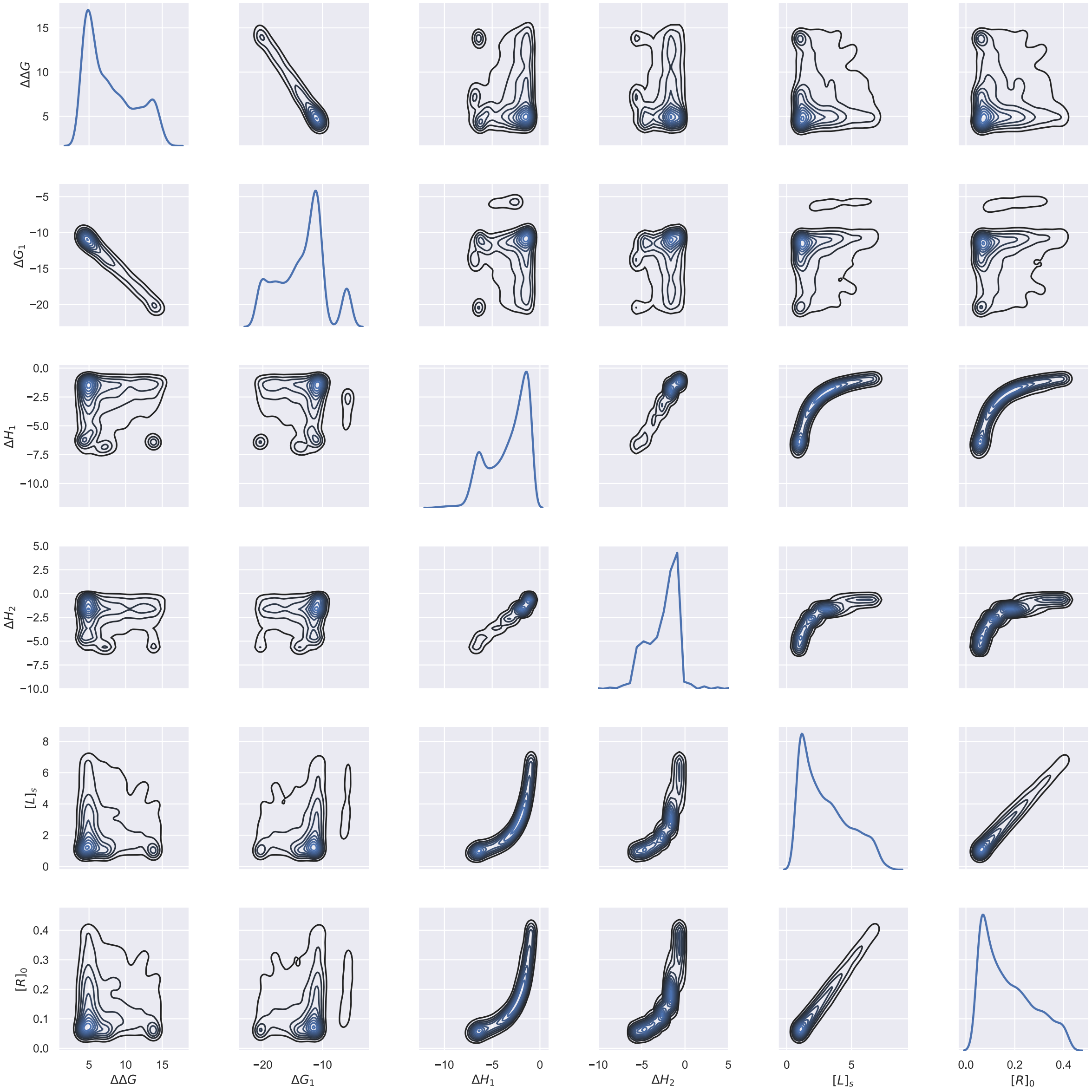

Baum_59

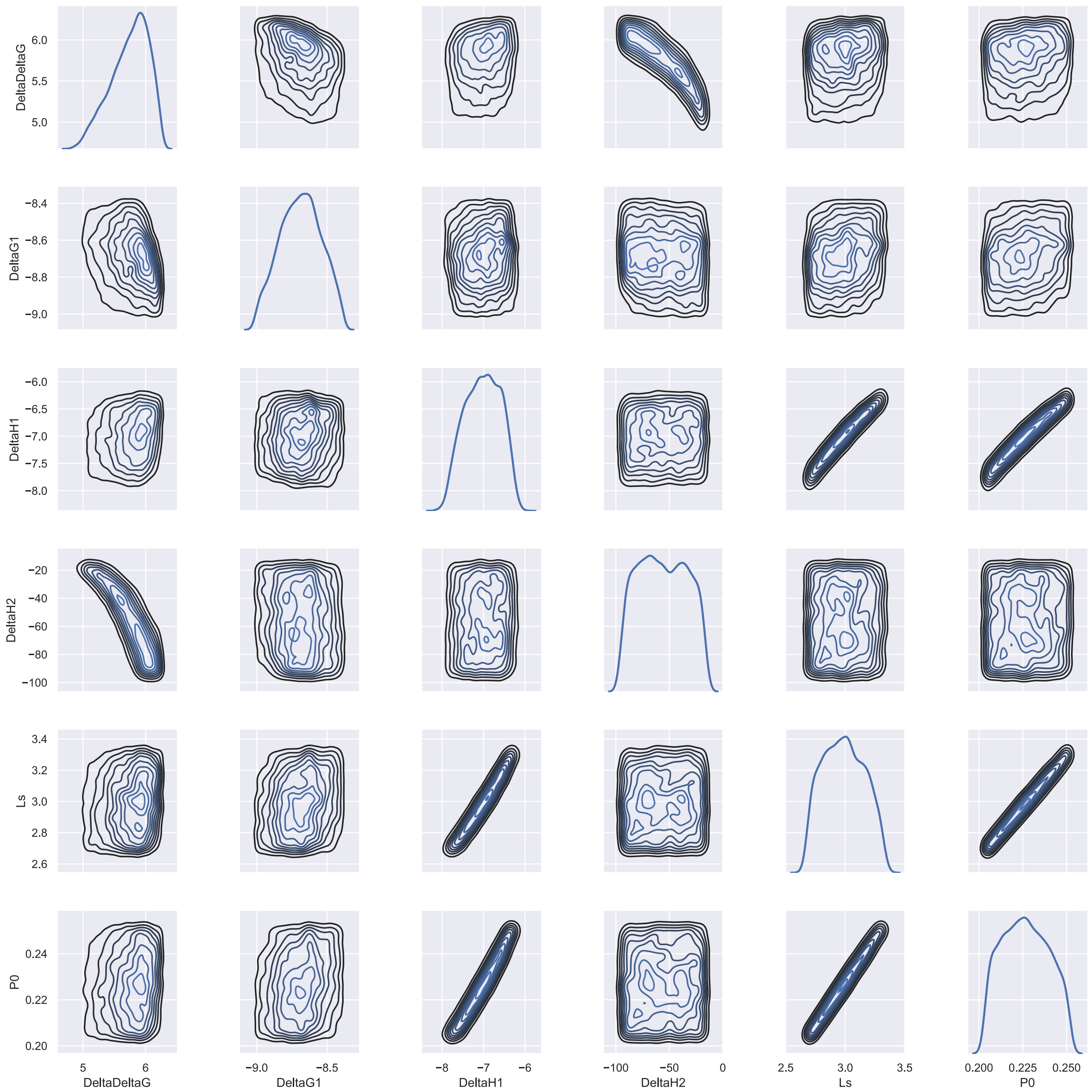
DeltaDeltaG
DeltaG1
DeltaH1
DeltaH2
Ls
P0
6.0
5.5
5.0
−8.4
−8.6
−8.8
−9.0
−6.0
−6.5
−7.0
−7.5
−8.0
−20
−40
−60
−80
−100
3.4
3.2
3.0
2.8
2.6
0.24
0.22
0.20
5
6
−9.0
−8.5
−8
−7
−6
−100
−50
0
2.5
3.0
3.5
0.200
0.225
0.250

Baum_60_1

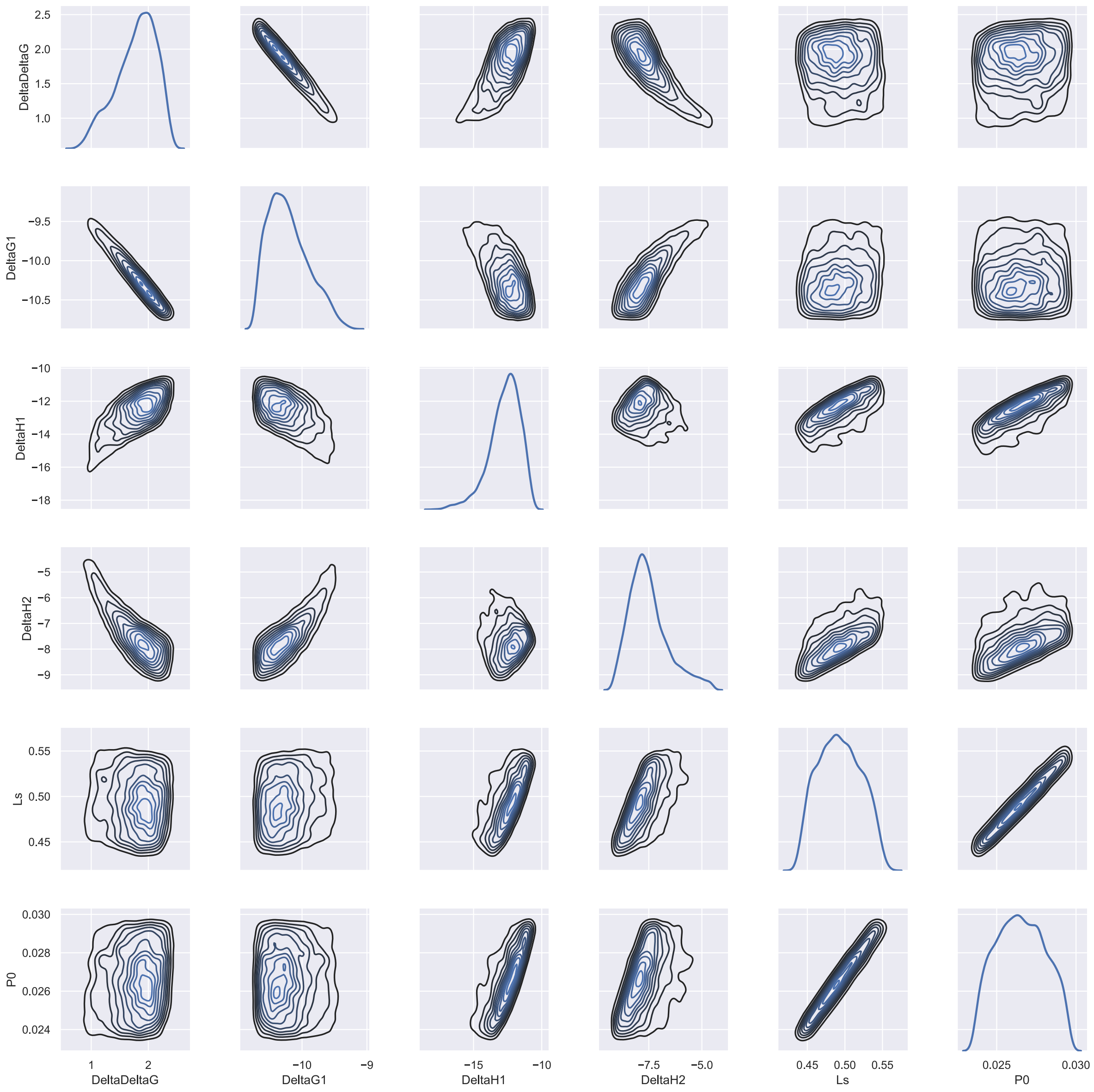

Baum_60_2

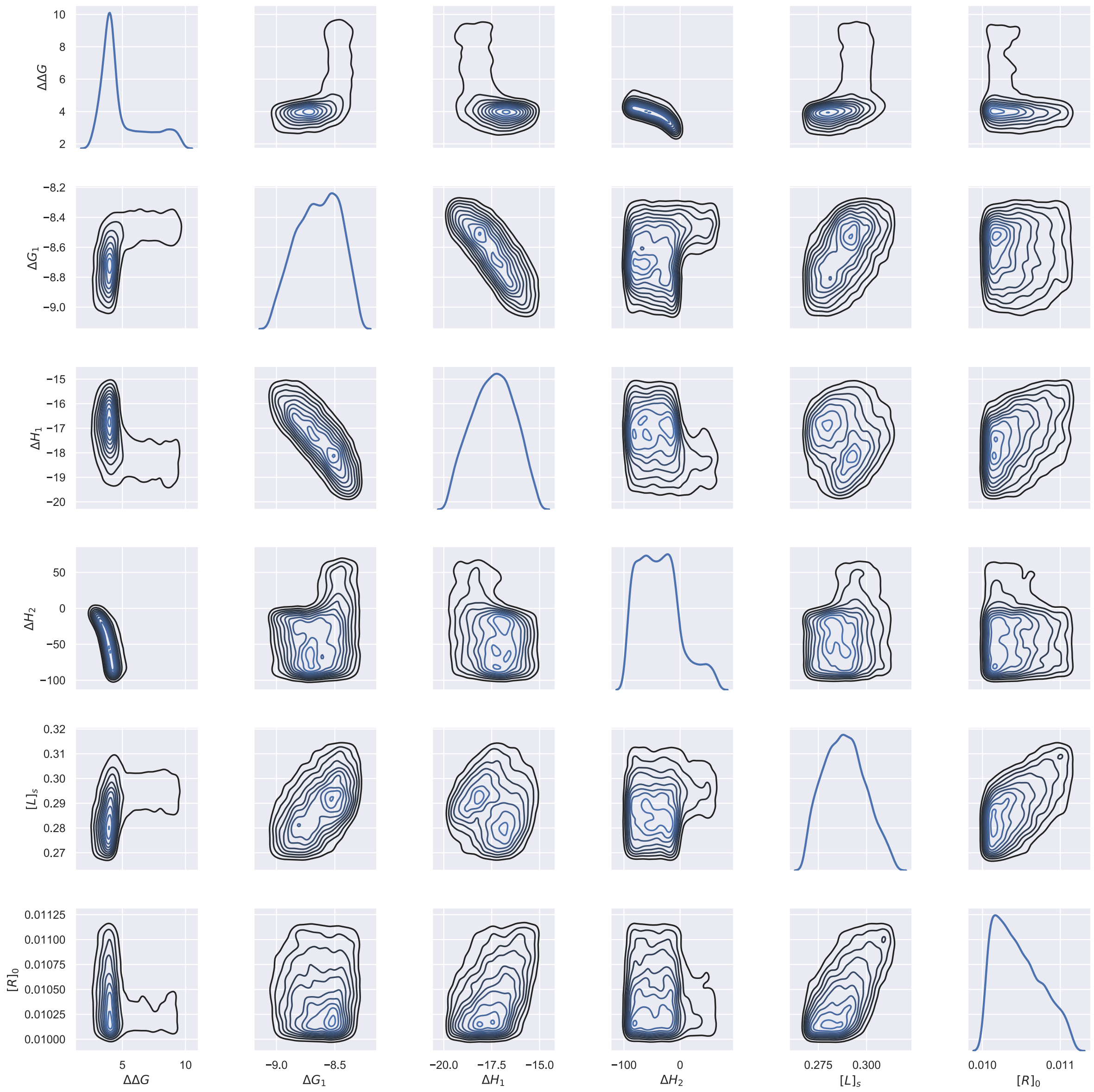

Baum_60_3

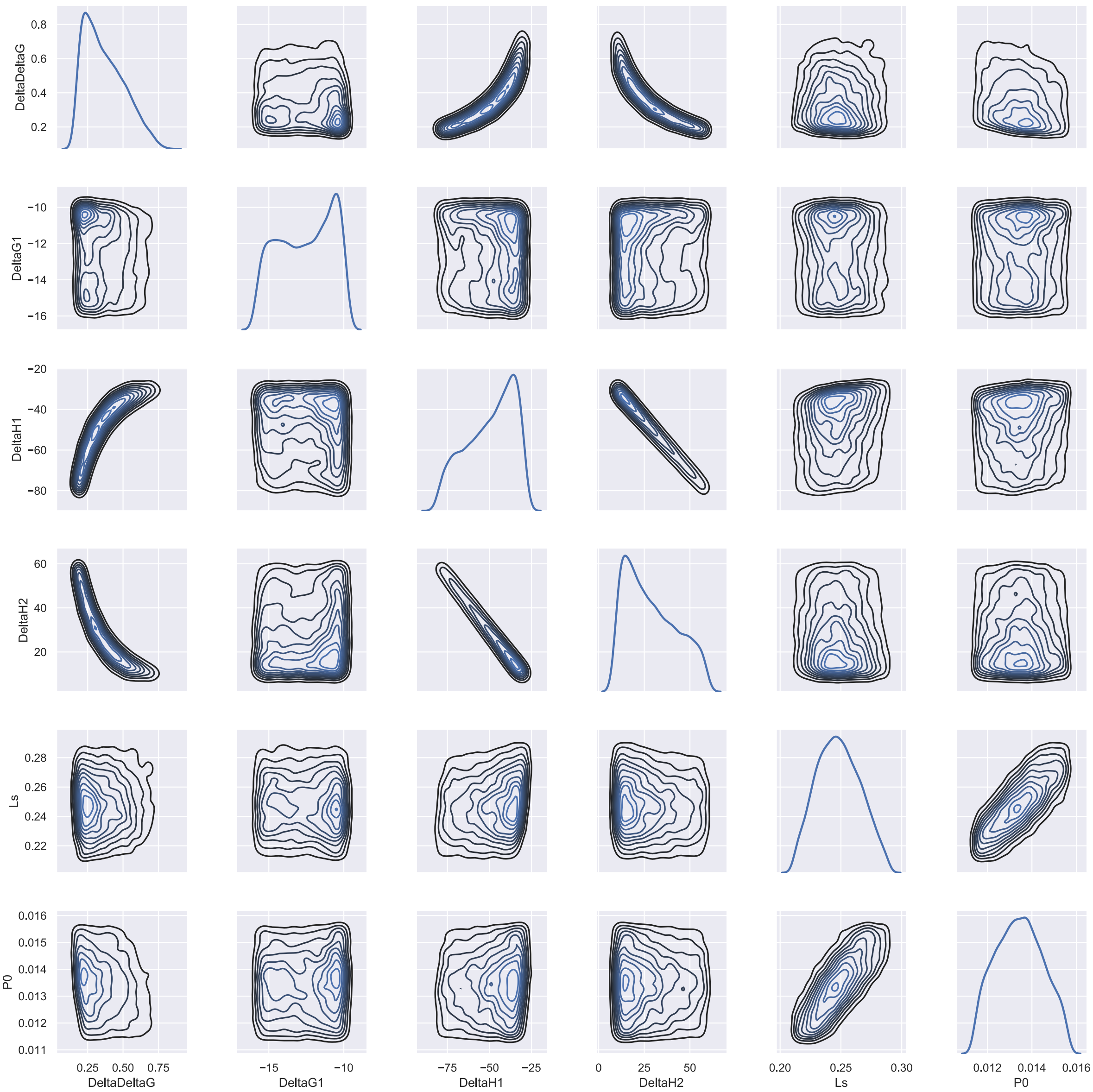
DeltaDeltaG
DeltaG1
DeltaH1
DeltaH2
Ls
P0
0.8
0.6
0.4
0.2
−10
−12
−14
−16
−20
−40
−60
−80
60
40
20
0.28
0.26
0.24
0.22
0.016
0.015
0.014
0.013
0.012
0.011
0.25 0.50 0.75
−15 −10
−75 −50 −25
0 25 50
0.20 0.25 0.30
0.012 0.014 0.016

Baum_60_4

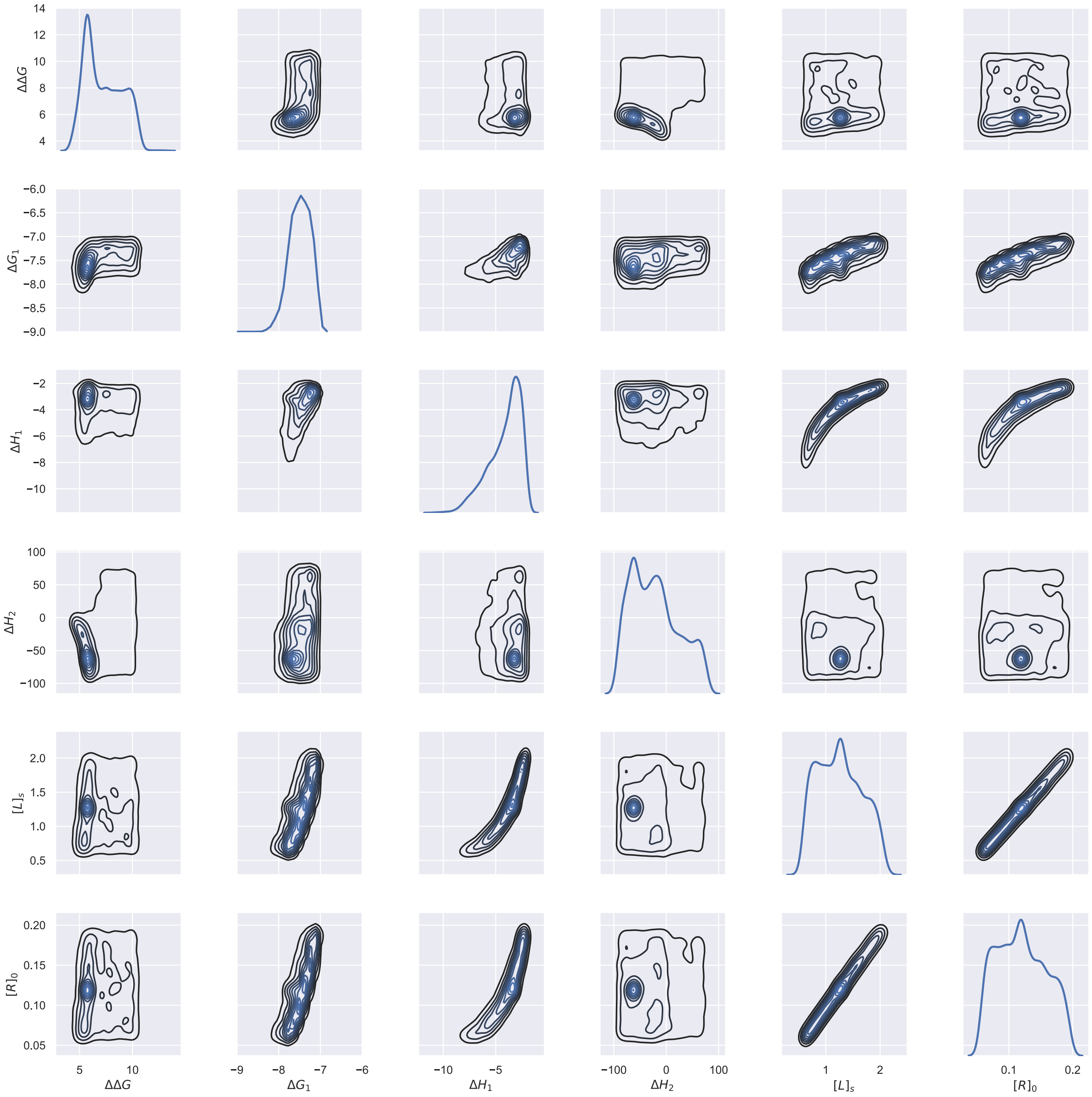
ΔΔG
ΔG_1
ΔH_1
ΔH_2
[L]_s
[R]_0
14 12 10 8 6 4
−6.0 −6.5 −7.0 −7.5 −8.0 −8.5 −9.0
−2 −4 −6 −8 −10
100 50 0 −50 −100
2.0 1.5 1.0 0.5
0.20 0.15 0.10 0.05
5 10
−9 −8 −7 −6
−10 −5
−100 0 100
1 2
0.1 0.2

Fokkens_1_a

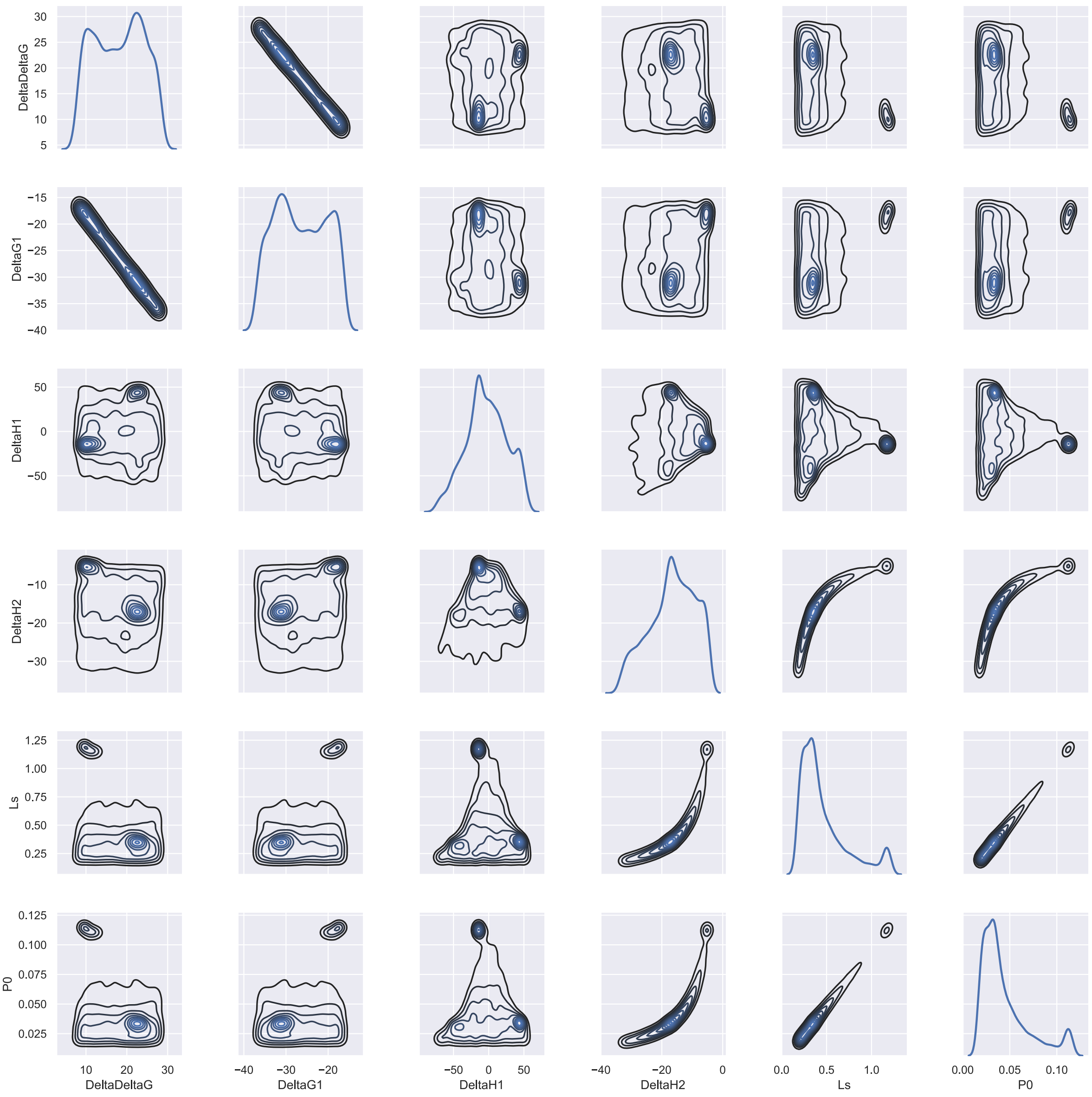

Fokkens_1_b

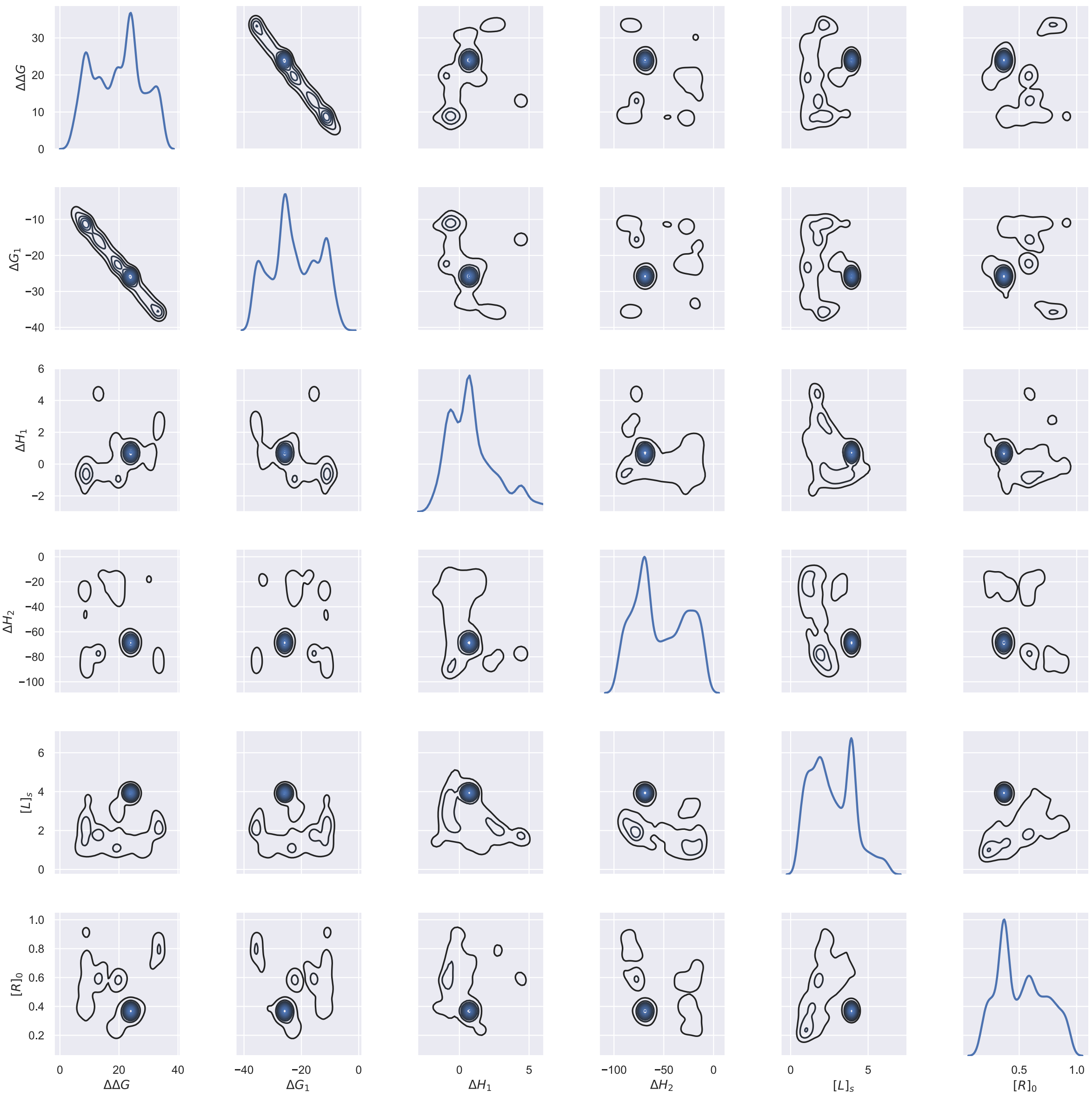

Fokkens_1_c

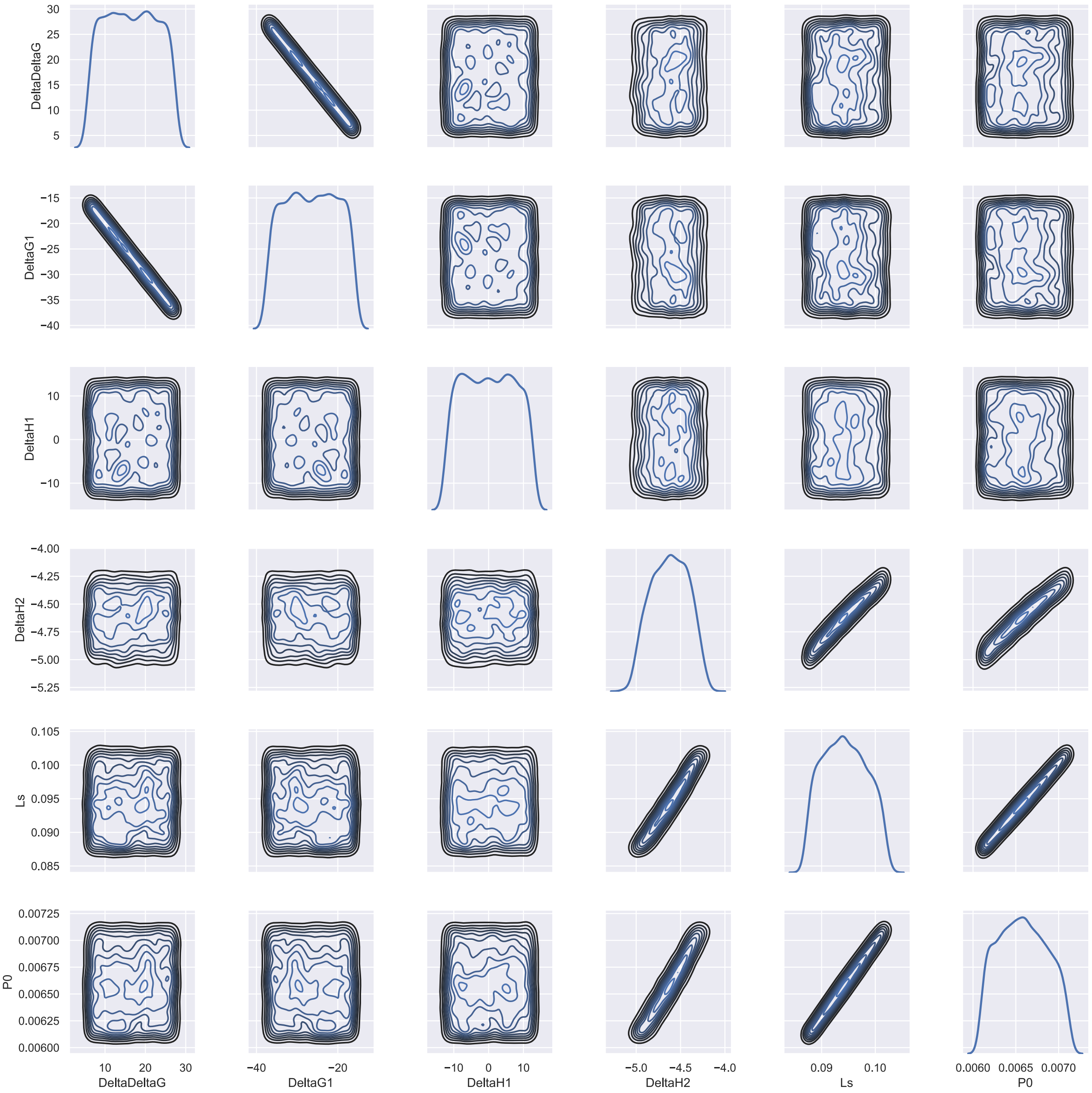
DeltaDeltaG
DeltaG1
DeltaH1
DeltaH2
Ls
P0
30 25 20 15 10 5
−15 −20 −25 −30 −35 −40
10 0 −10
−4.00 −4.25 −4.50 −4.75 −5.00 −5.25
0.105 0.100 0.095 0.090 0.085
0.00725 0.00700 0.00675 0.00650 0.00625 0.00600
10 20 30
−40 −20
−10 0 10
−5.0 −4.5 −4.0
0.09 0.10
0.0060 0.0065 0.0070
DeltaDeltaG
DeltaG1
DeltaH1
DeltaH2
Ls
P0

Fokkens_1_d

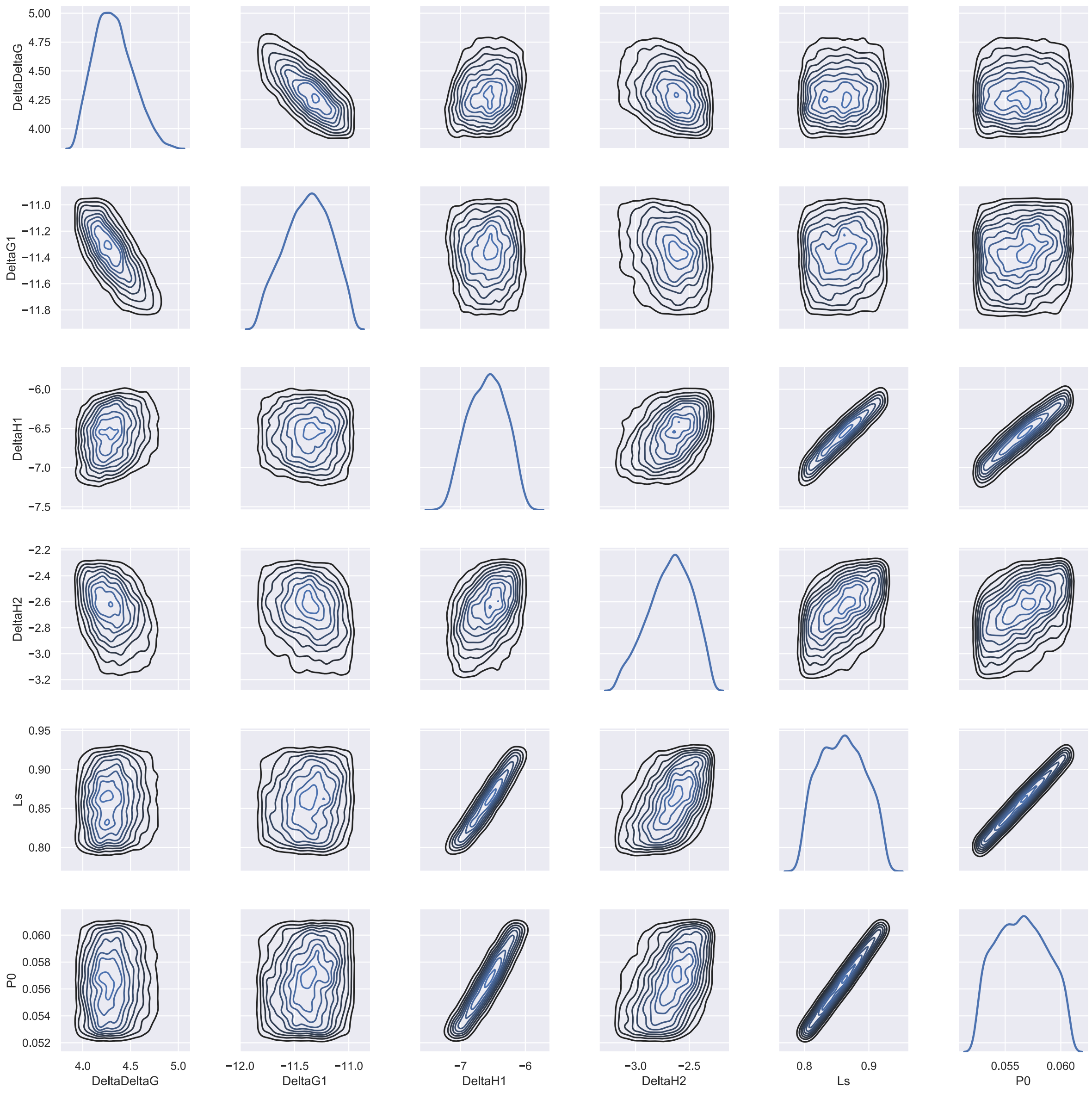
DeltaDeltaG
DeltaG1
DeltaH1
DeltaH2
Ls
P0
5.00
4.75
4.50
4.25
4.00
−11.0
−11.2
−11.4
−11.6
−11.8
−6.0
−6.5
−7.0
−7.5
−2.2
−2.4
−2.6
−2.8
−3.0
−3.2
0.95
0.90
0.85
0.80
0.060
0.058
0.056
0.054
0.052
4.0
4.5
5.0
−12.0
−11.5
−11.0
−7
−6
−3.0
−2.5
0.8
0.9
0.055
0.060
DeltaDeltaG
DeltaG1
DeltaH1
DeltaH2
Ls
P0

Fokkens_1_e

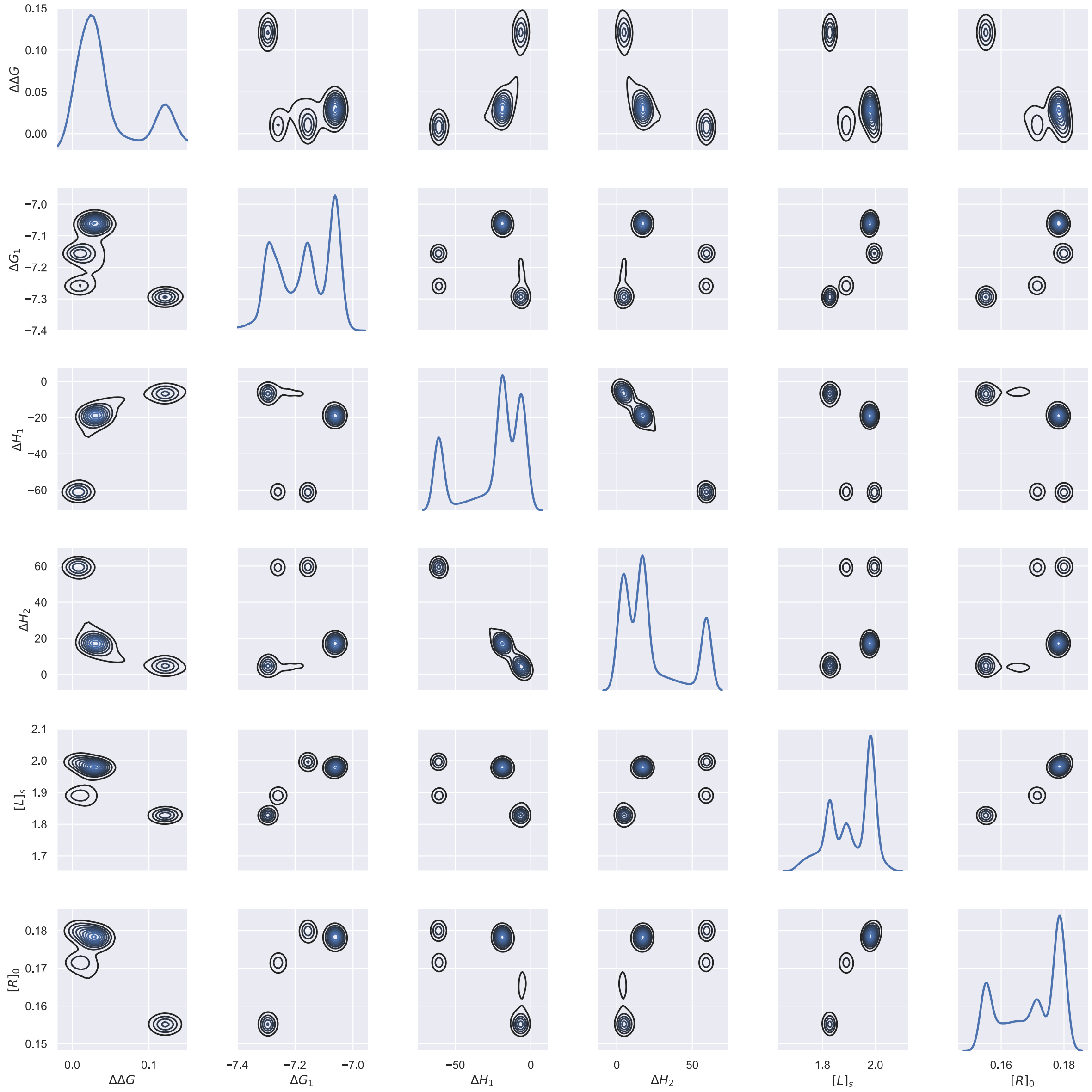

Supplement: S3 Appendix — (PDF) [file pone.0273656.s003.pdf]
