# Supplementary material for: Bayesian regression and model selection for isothermal titration calorimetry with enantiomeric mixtures
Source: PLoS One. 2022 Sep 29;17(9):e0273656. doi: 10.1371/journal.pone.0273656 (PMC9521810; doi:10.1371/journal.pone.0273656)

Enantiomer model

Baum_57

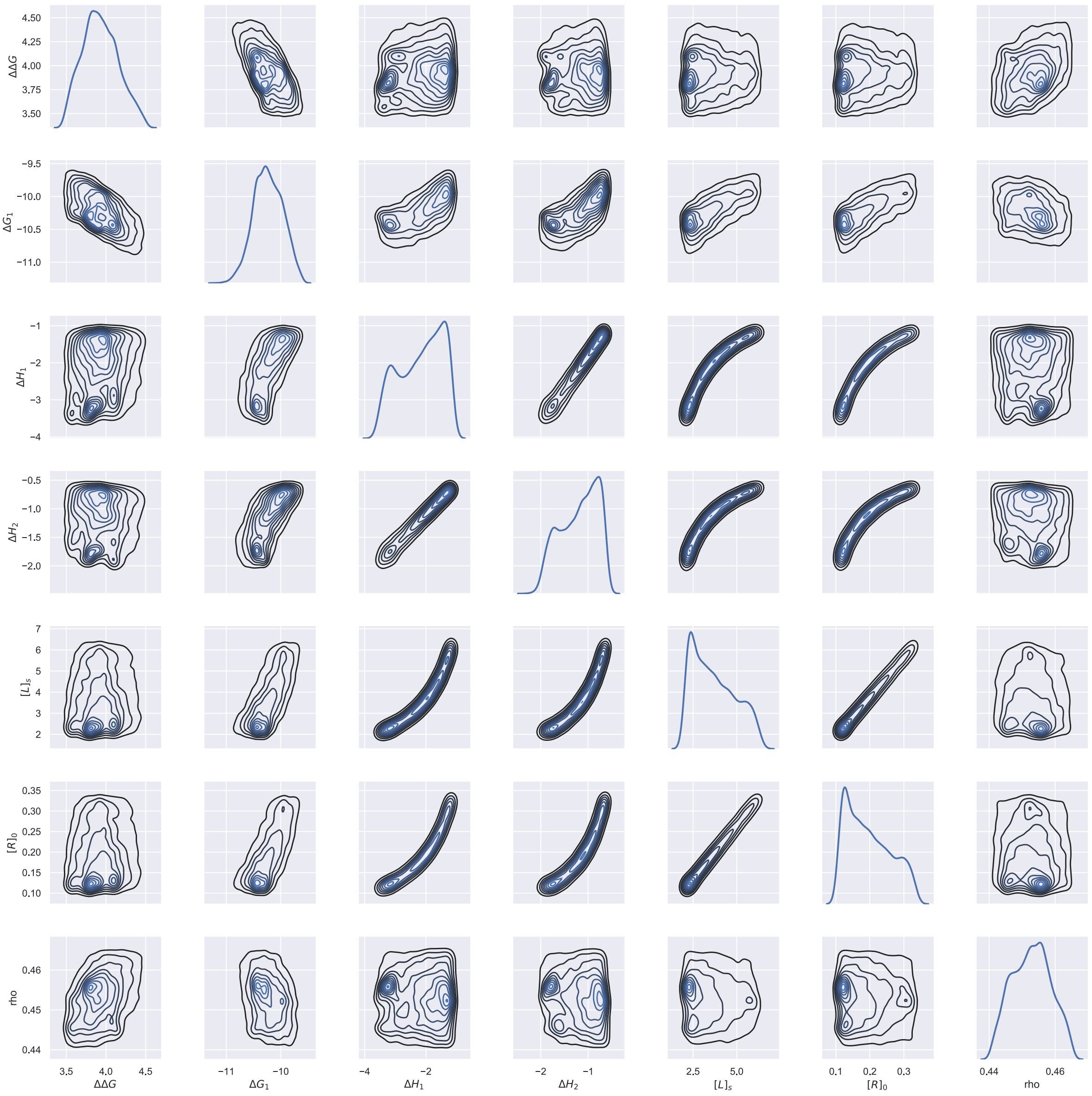

Baum_59

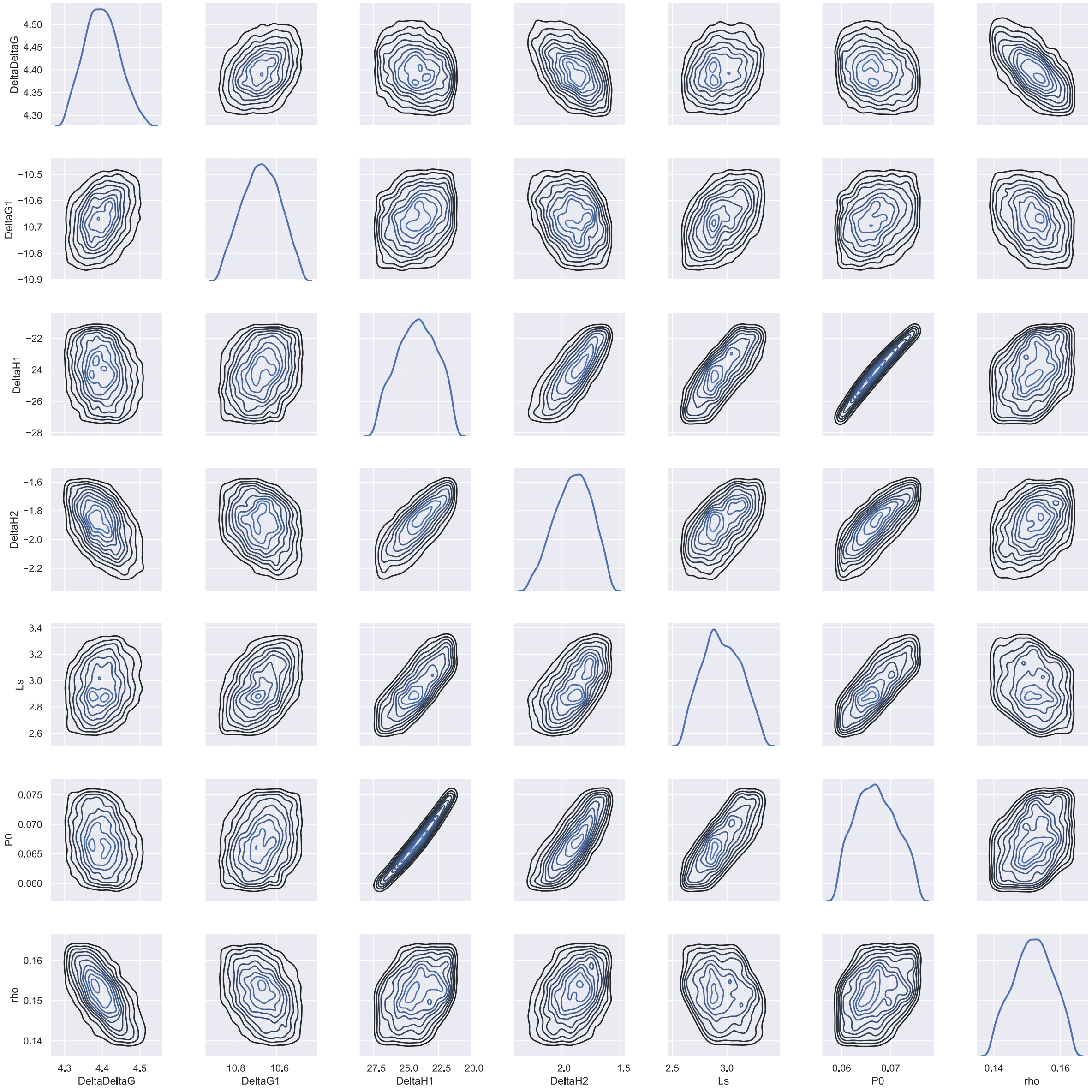

DeltaDeltaG
DeltaG1
DeltaH1
DeltaH2
Ls
P0
rho
4.3
4.4
4.5
−10.8
−10.6
−27.5
−25.0
−22.5
−20.0
−2.0
−1.5
2.5
3.0
0.06
0.07
0.14
0.16
4.50
4.45
4.40
4.35
4.30
−10.5
−10.6
−10.7
−10.8
−10.9
−22
−24
−26
−28
−1.6
−1.8
−2.0
−2.2
3.4
3.2
3.0
2.8
2.6
0.075
0.070
0.065
0.060
0.16
0.15
0.14

Baum_60_1

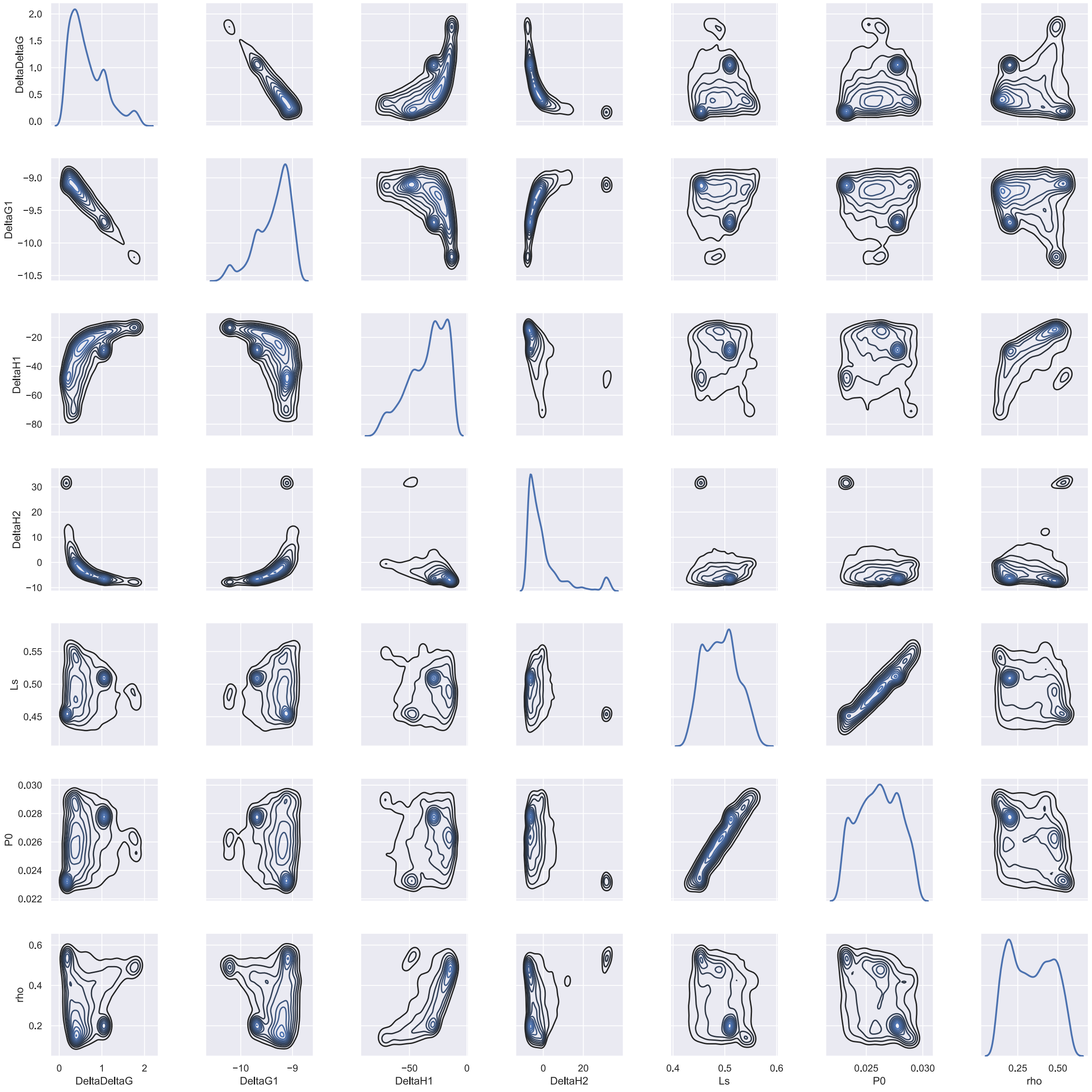

Baum_60_2

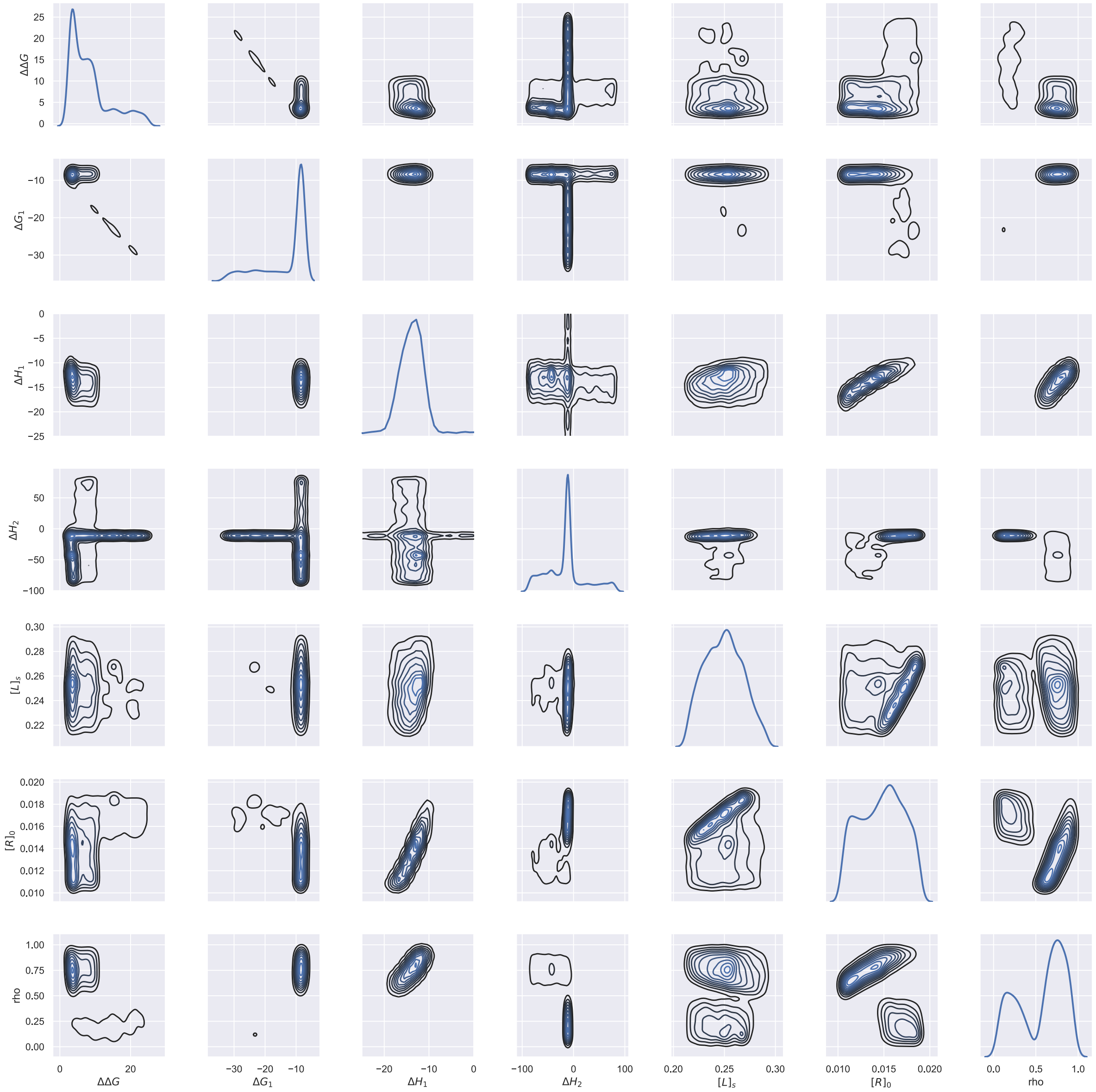

Baum_60_3

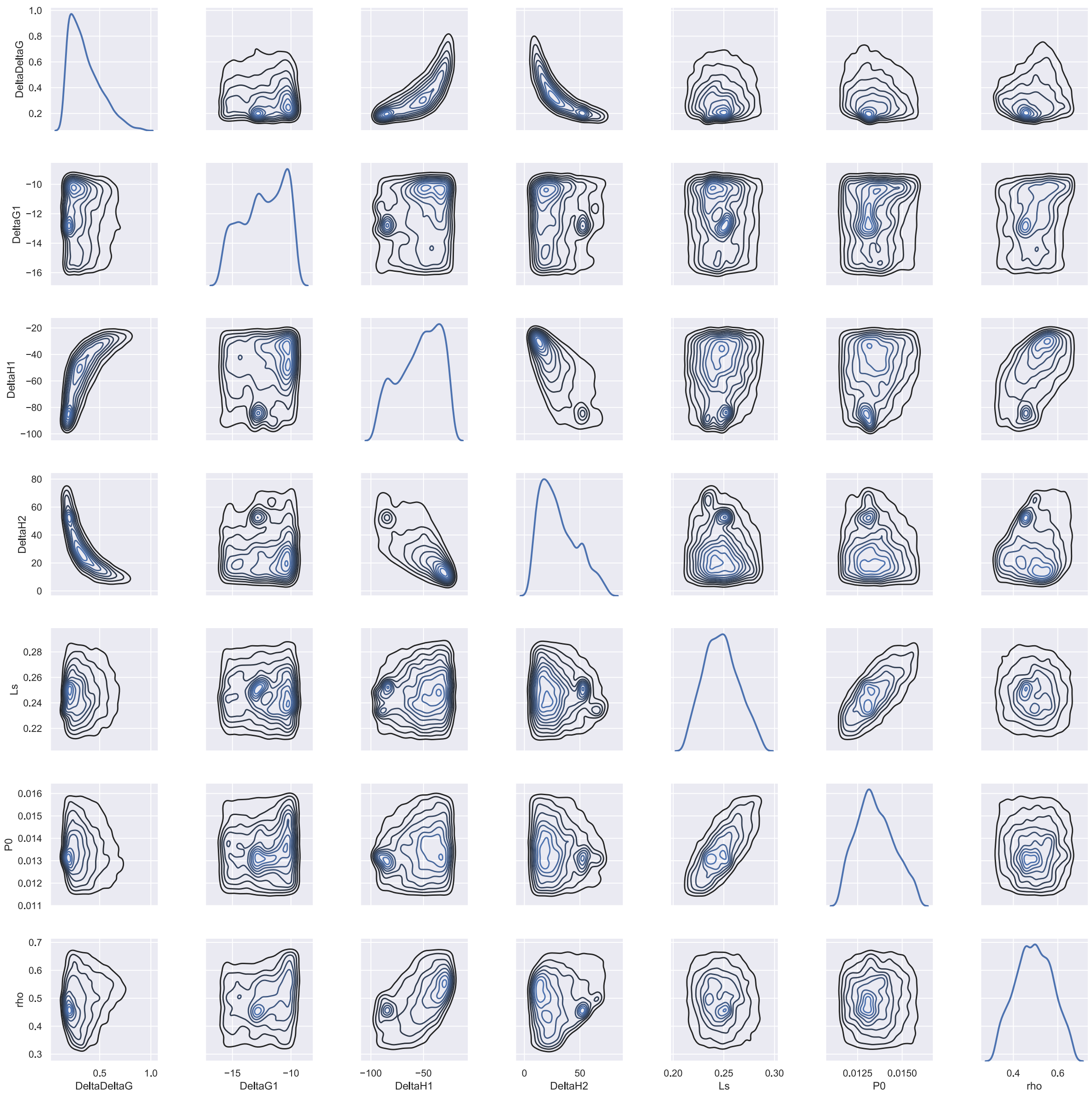

Baum_60_4

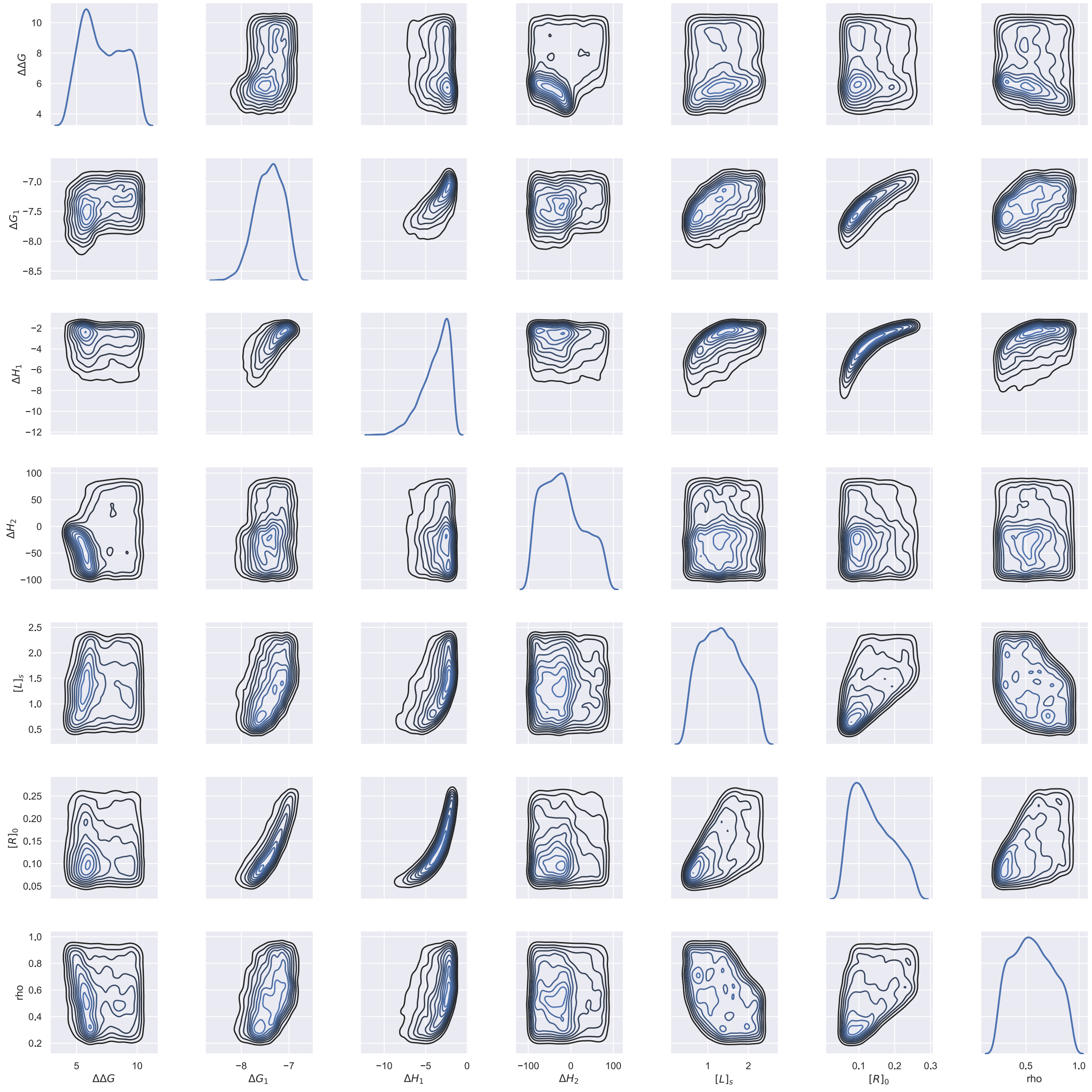

Fokkens_1_a

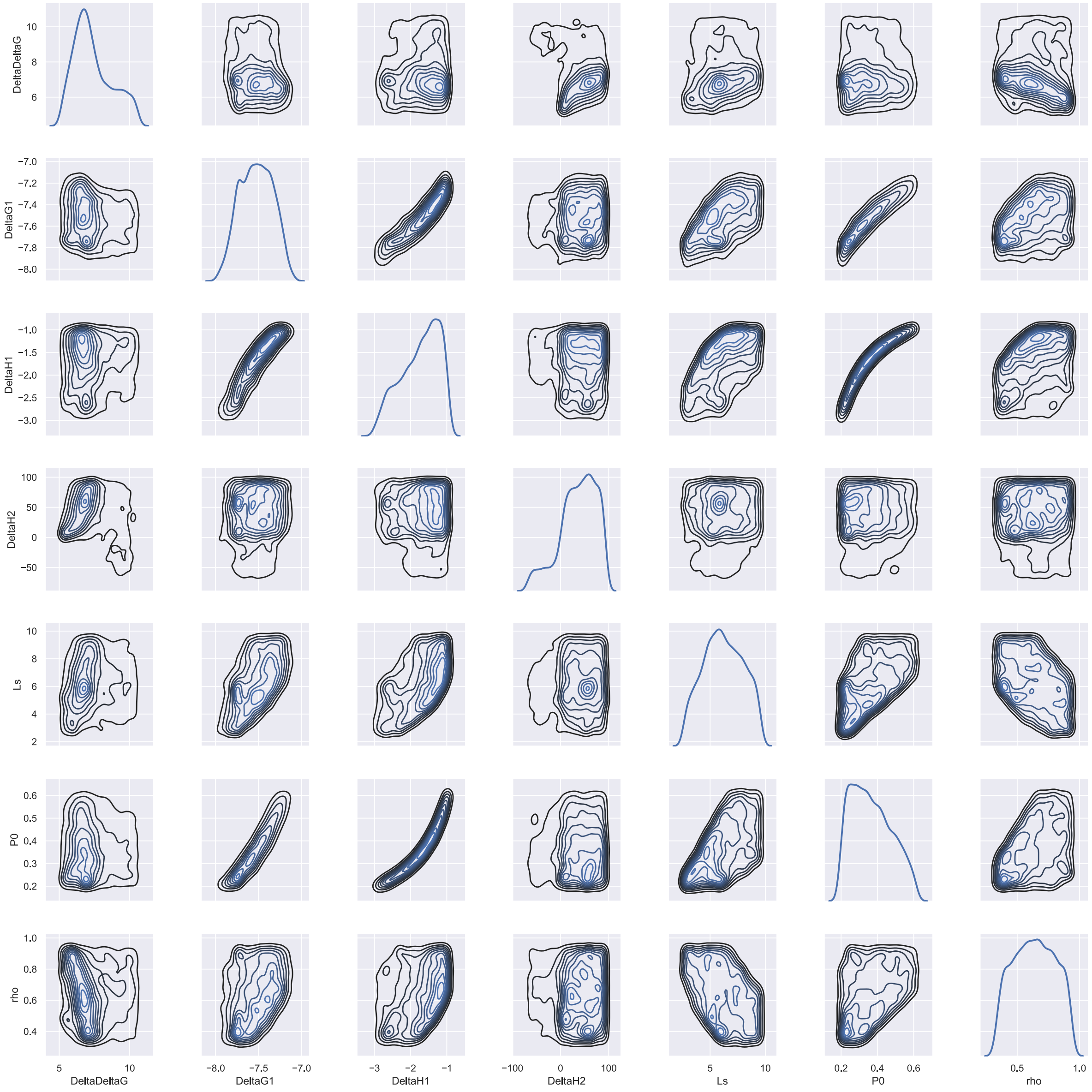

Fokkens_1_b

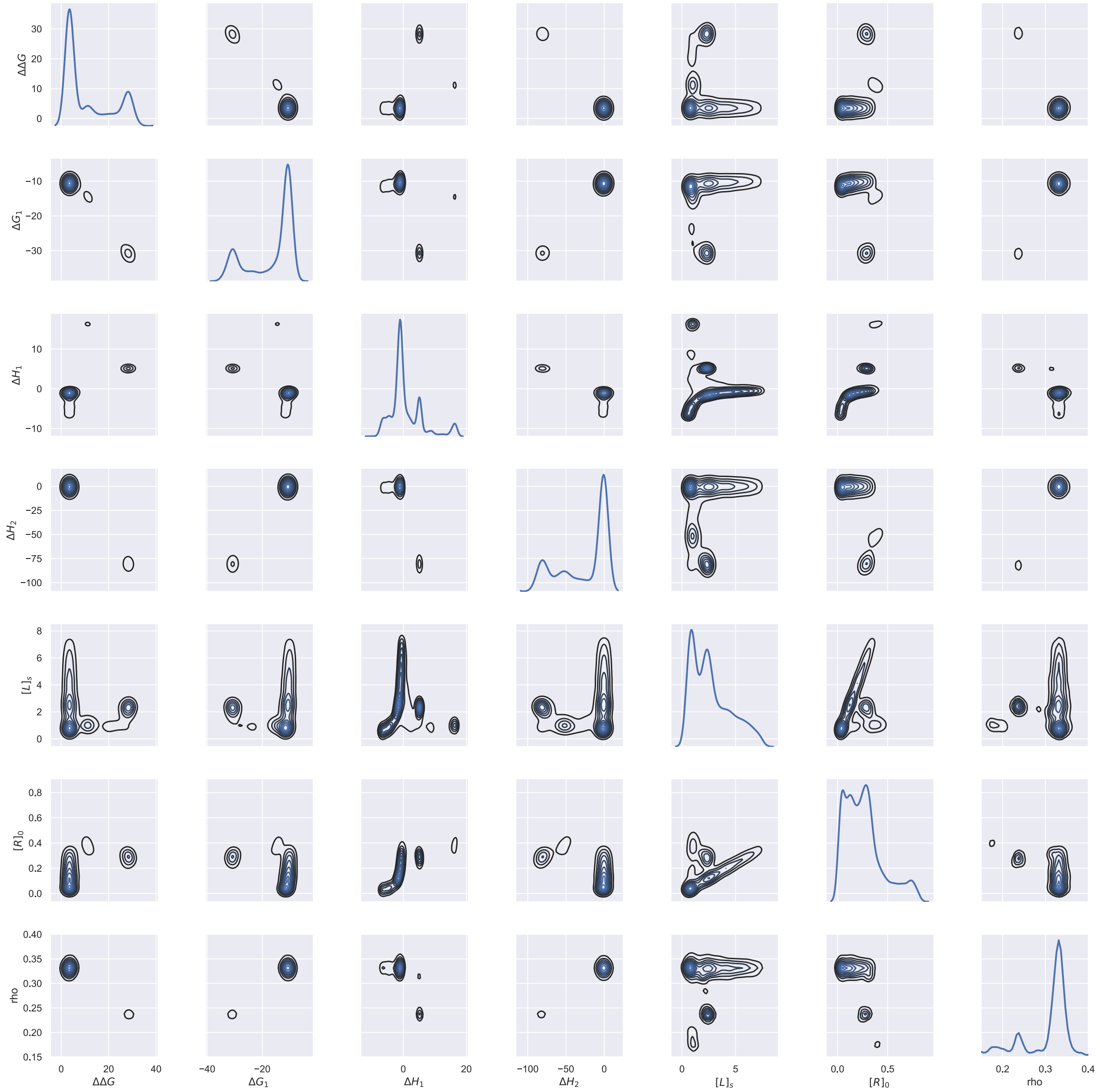

ΔΔG
ΔG1
ΔH1
ΔH2
[L]s
[R]0
rho
30
20
10
0
−10
−20
−30
10
0
−10
0
−25
−50
−75
−100
8
6
4
2
0
0.8
0.6
0.4
0.2
0.0
0.40
0.35
0.30
0.25
0.20
0.15
0
20
40
−40
−20
0
20
−100
−50
0
0
5
0.0
0.5
0.2
0.3
0.4

Fokkens_1_c

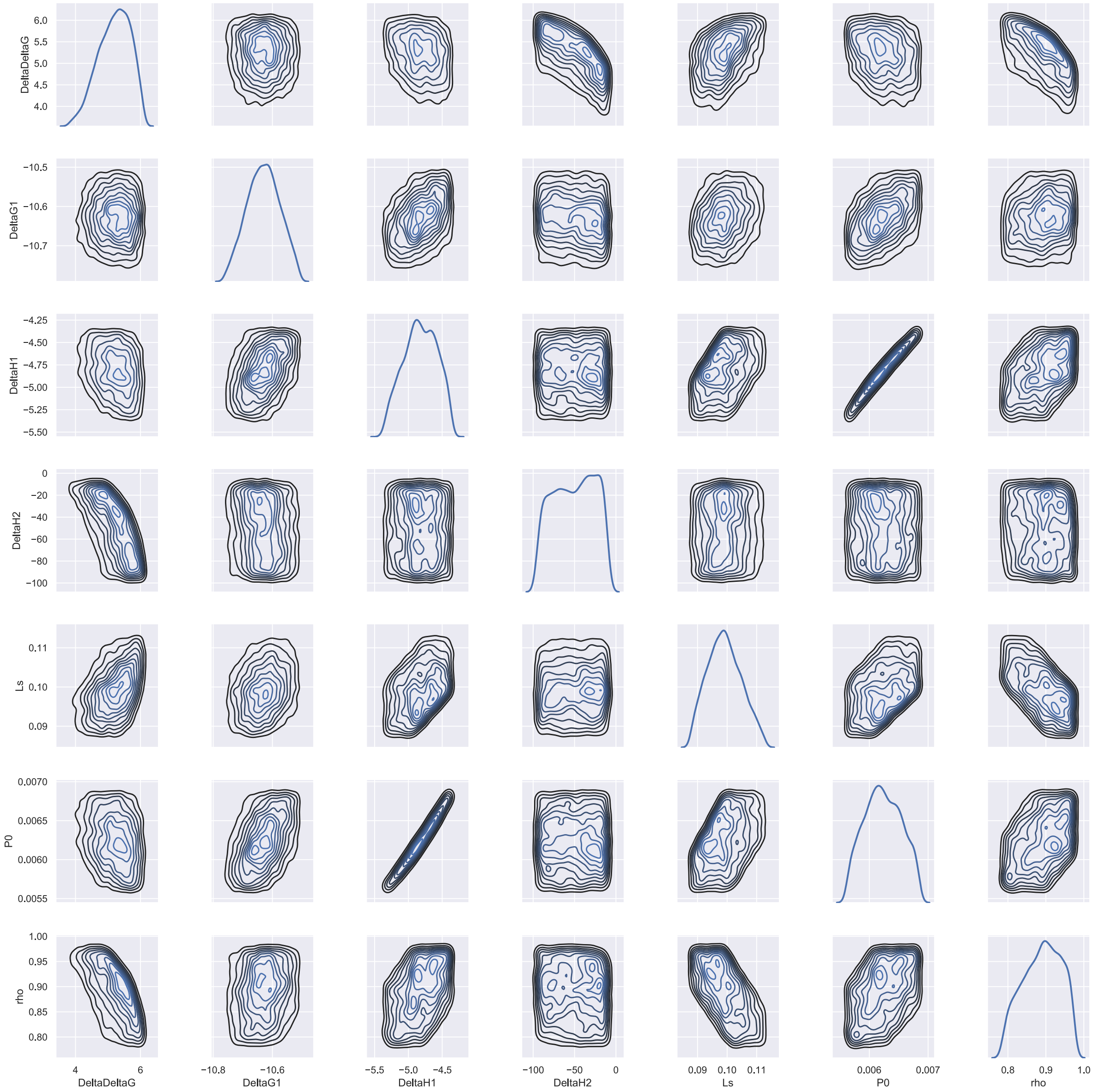
DeltaDeltaG
DeltaG1
DeltaH1
DeltaH2
Ls
P0
rho
6.0
5.5
5.0
4.5
4.0
−10.5
−10.6
−10.7
−4.25
−4.50
−4.75
−5.00
−5.25
−5.50
0
−20
−40
−60
−80
−100
0.11
0.10
0.09
0.0070
0.0065
0.0060
0.0055
1.00
0.95
0.90
0.85
0.80
4
6
−10.8
−10.6
−5.5
−5.0
−4.5
−100
−50
0
0.09
0.10
0.11
0.006
0.007
0.8
0.9
1.0

Fokkens_1_d

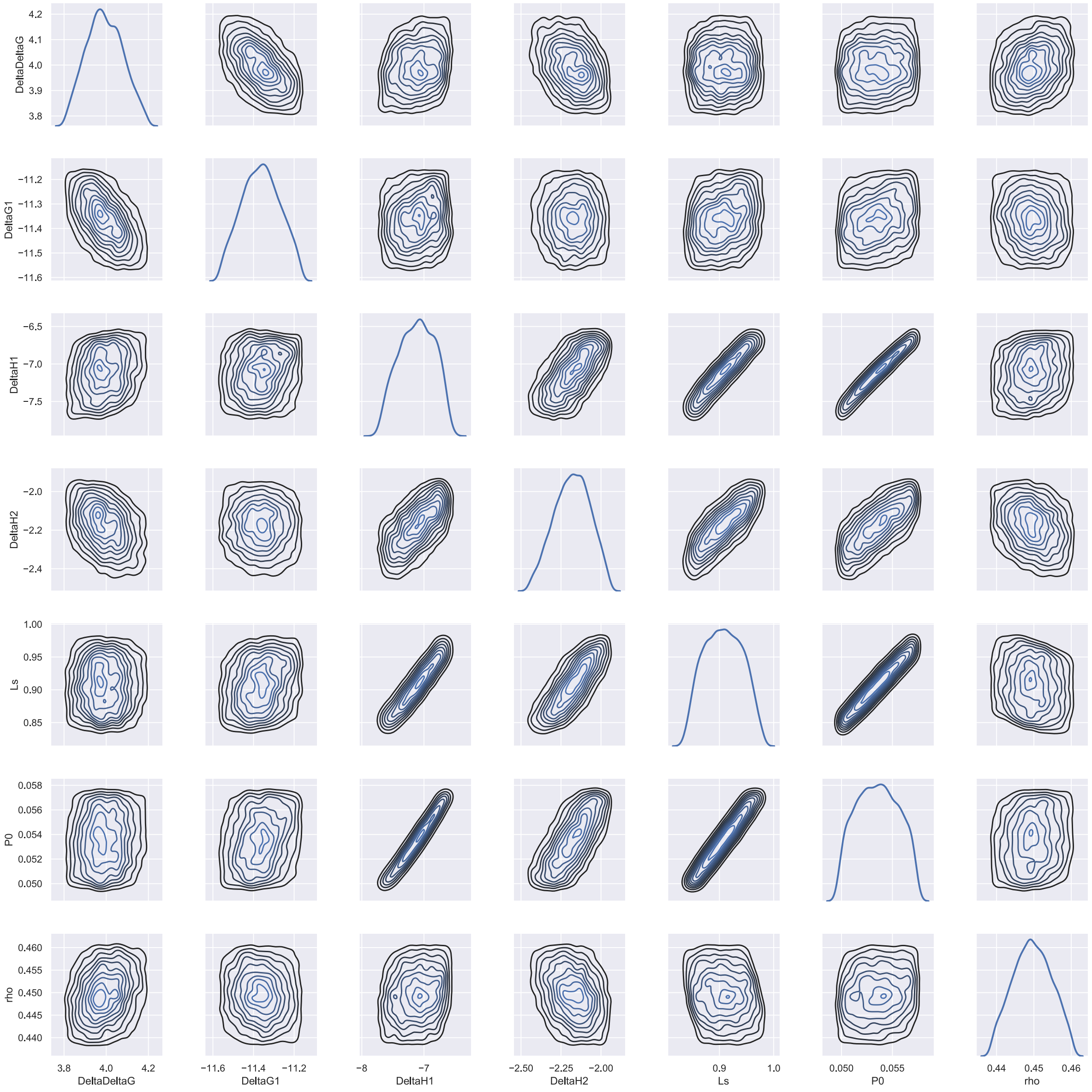
DeltaDeltaG
DeltaG1
DeltaH1
DeltaH2
Ls
P0
rho
4.2
4.1
4.0
3.9
3.8
−11.2
−11.3
−11.4
−11.5
−11.6
−6.5
−7.0
−7.5
−2.0
−2.2
−2.4
1.00
0.95
0.90
0.85
0.058
0.056
0.054
0.052
0.050
0.460
0.455
0.450
0.445
0.440
3.8
4.0
4.2
−11.6
−11.4
−11.2
−8
−7
−2.50
−2.25
−2.00
0.9
1.0
0.050
0.055
0.44
0.45
0.46

Fokkens_1_e

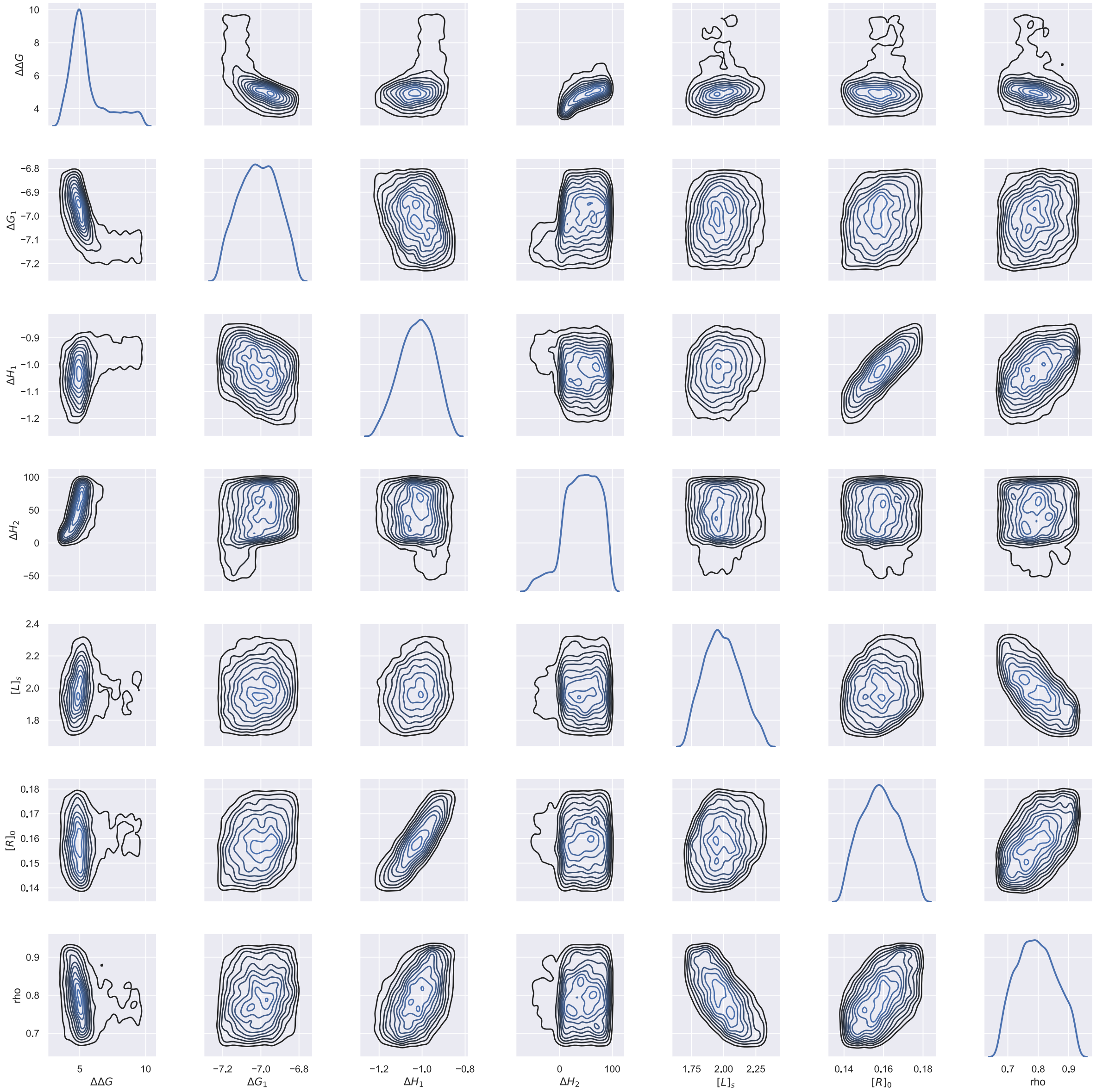

Simulation Data - Priors for the concentration were lognormal for both [L]s and [R]0

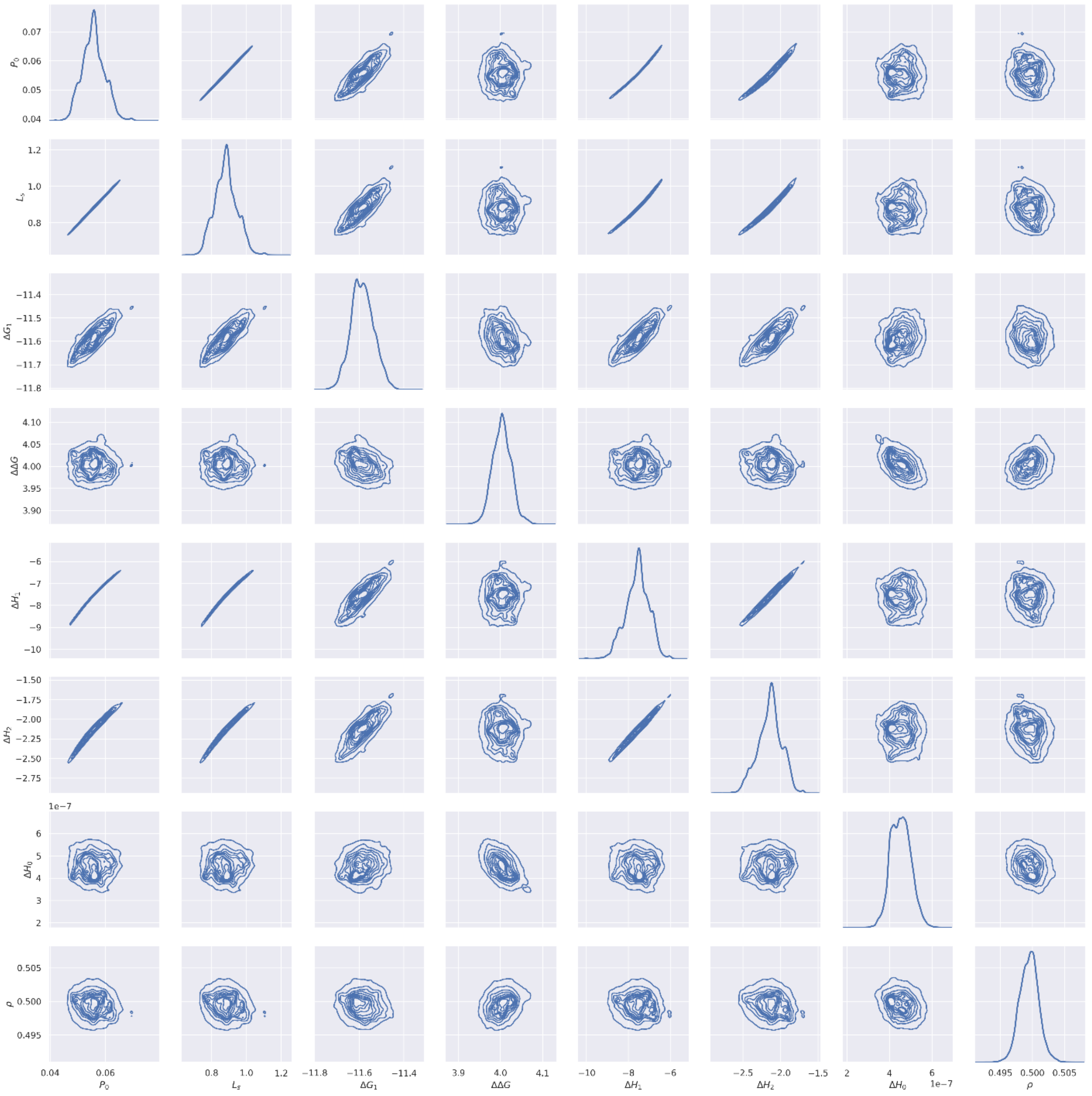

Simulation Data - Priors for the concentration were lognormal for $[L]_s$ and uniform for $[R]_0$

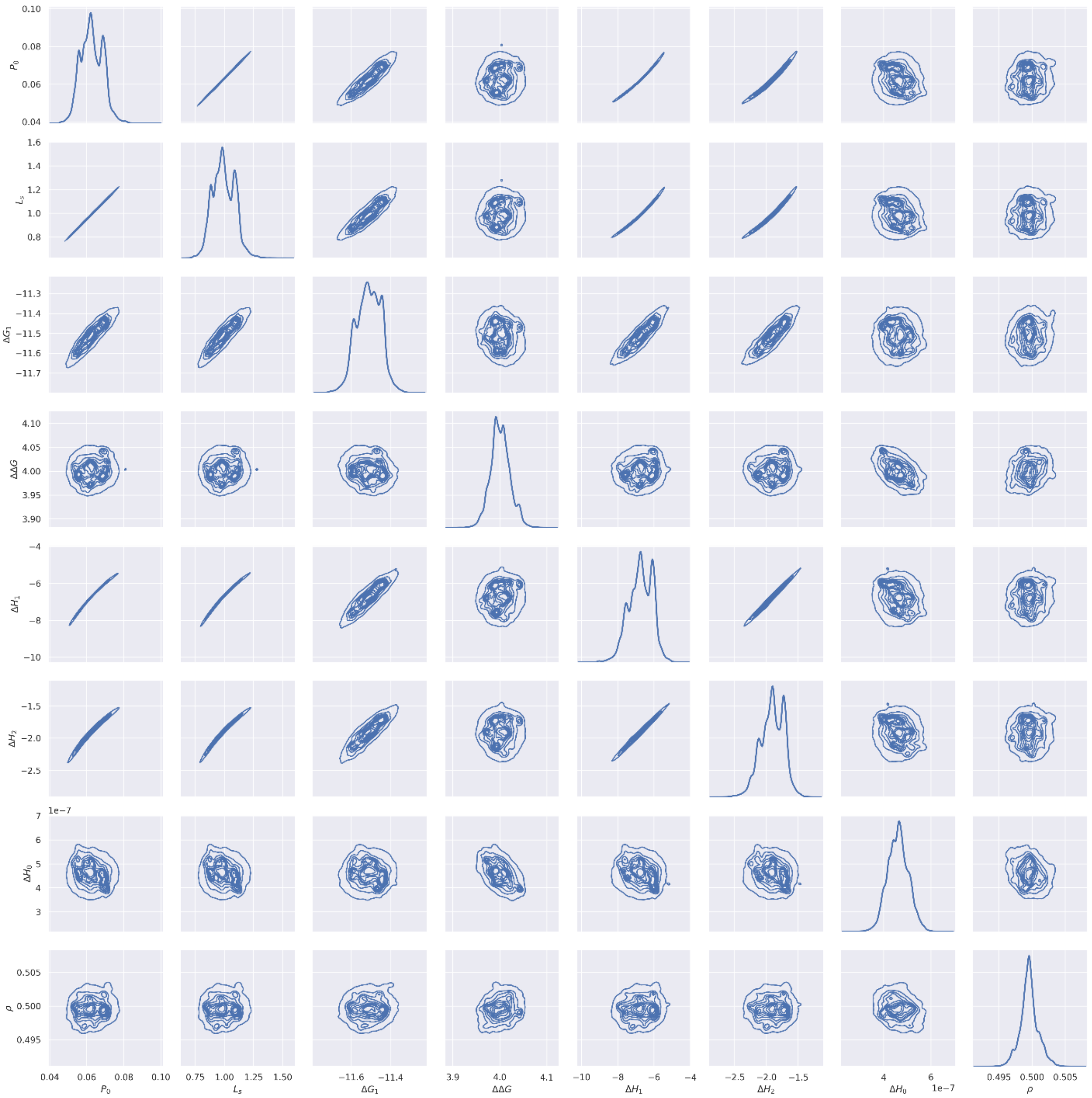

Simulation Data - Priors for the concentration were uniform for $[L]_s$ and lognormal for $[R]_0$

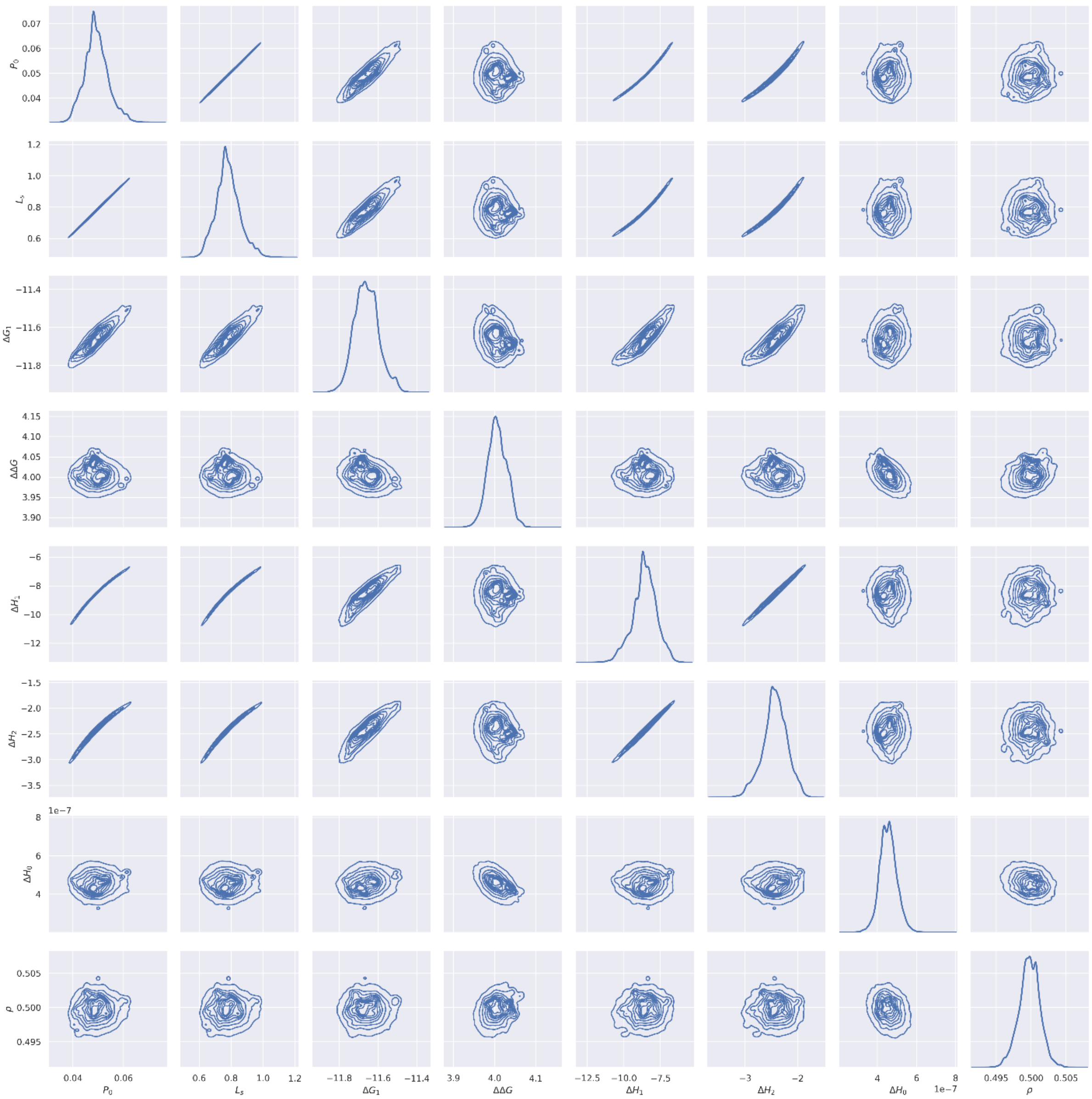

Simulation Data - Priors for the concentration were uniform for both $[L]_s$ and $[R]_0$

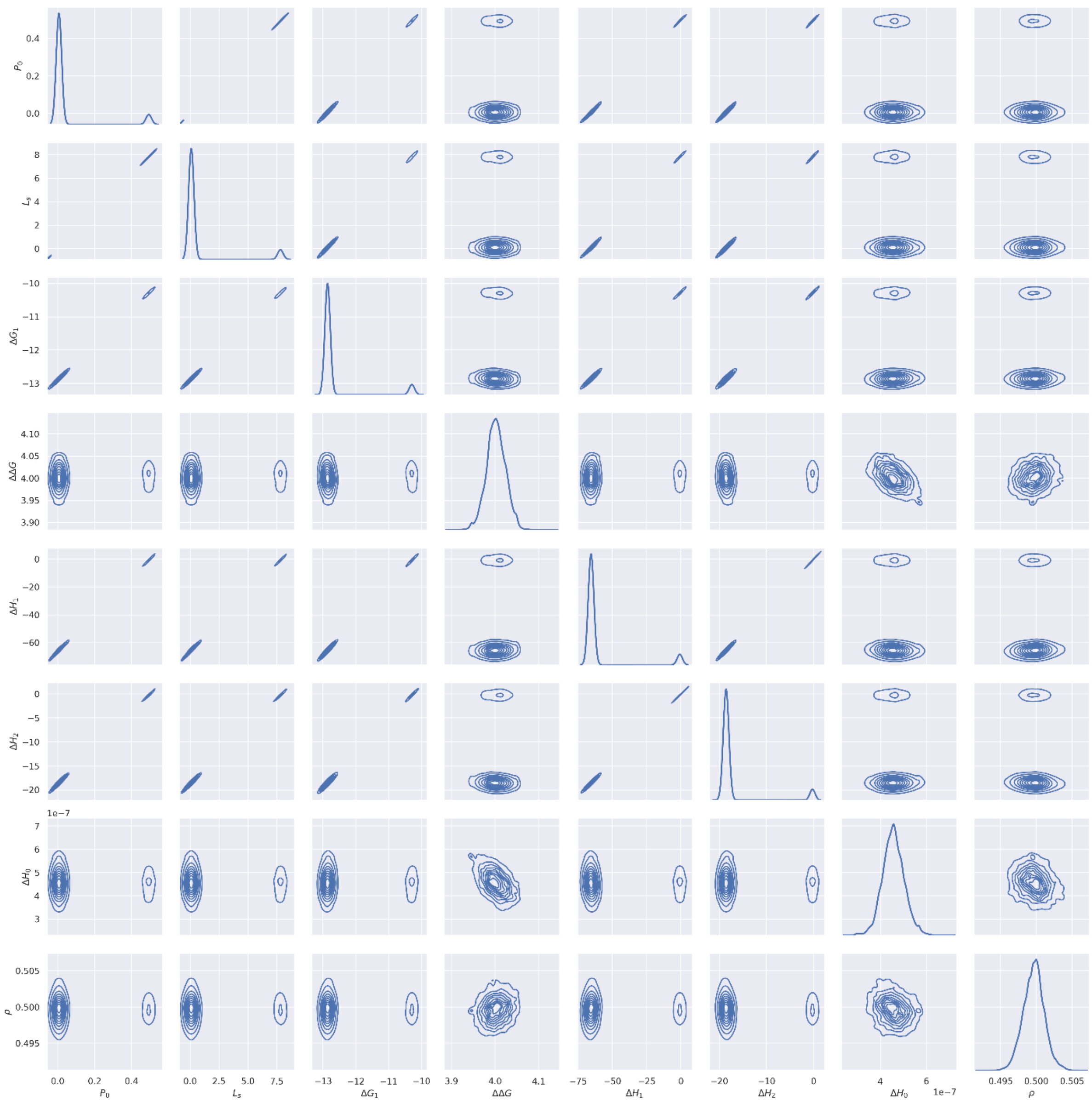

Supplement: S4 Appendix — (PDF) [file pone.0273656.s004.pdf]
