# Supplementary material for: Bayesian regression and model selection for isothermal titration calorimetry with enantiomeric mixtures
Source: PLoS One. 2022 Sep 29;17(9):e0273656. doi: 10.1371/journal.pone.0273656 (PMC9521810; doi:10.1371/journal.pone.0273656)
Supplement: S1 Table — (PDF) [file pone.0273656.s005.pdf]

Dataset	Titrand (Macro-molecule)	Titrant (small molecule)	Cell concentration (mM)	Syringe concentration (mM)	Initial injection volume (L)	Main injection volume (L)	Number of injections
Fokkens_1a	Trypsin	D-Napap (**8**)	-	-	1.5	10	23
Fokkens_1b	Trypsin	*rac*-Napap (**8**)	-	-	1.5	10	23
Fokkens_1c	Thrombin	*rac*-**1**	0.0065	0.0975	1.5	10	17
Fokkens_1d	Thrombin	*rac*-**2**	0.05	1.0	1.5	10	24
Fokkens_1e	Trypsin	*rac*-**1**	0.16	2.0	1.5	10	22
Baum_57	Thrombin	*rac*-**2**	-	-		10	21
Baum_59	Trypsin	UB_THR_32 + n-pentyl-Benzamindin	-	3.0	1.5	10	39
Baum_60_1	Trypsin	CJ802 + CJ903	0.10	0.5	1.5	10	19
Baum_60_2	Trypsin	CJ802 + CJ903	0.10	0.25	1.5	8	23
Baum_60_3	Trypsin	CJ802 + CJ903	0.10	0.25	1.5	10	20
Baum_60_4	Trypsin	CJ802 + CJ903	0.10	0.25	1.5	10	29
