# Supplementary material for: Bayesian regression and model selection for isothermal titration calorimetry with enantiomeric mixtures
Source: PLoS One. 2022 Sep 29;17(9):e0273656. doi: 10.1371/journal.pone.0273656 (PMC9521810; doi:10.1371/journal.pone.0273656)
Supplement: S2 Table — (PDF) [file pone.0273656.s006.pdf]

Model Selection

Dataset	Best model according to Bayes factors	Best model according to AIC	Best model according to BIC
Fokkens_1a	2C	EM	2C
Fokkens_1e	2C	RM	RM
Baum_60_2	2C	RM	2C
Fokkens_1c	EM	EM	EM
Baum_57	EM	EM	EM
Baum_59	EM	EM	EM
Fokkens_1b	RM and EM	EM	EM
Fokkens_1d	RM and EM	EM	EM
Baum_60_1	RM and EM	EM	RM
Baum_60_3	Inconclusive	RM	RM
Baum_60_4	Inconclusive	RM	2C

AIC

Dataset	2C	RM	EM
Fokkens_1a	64.713 (0.006)	68.333 (0.057)	64.167 (0.027)
Fokkens_1e	5.714 (0.024)	1.225 (0.011)	3.505 (0.023)
Baum_60_2	46.430 (0.023)	44.581 (0.063)	47.033 (0.027)
Fokkens_1c	-30.707 (0.013)	-29.183 (0.280)	-55.458 (0.100)
Baum_57	96.584 (0.008)	73.485 (0.146)	47.675 (0.040)
Baum_59	276.125 (0.017)	229.906 (0.007)	118.746 (0.071)
Fokkens_1b	80.282 (0.013)	76.457 (0.161)	32.496 (0.059)
Fokkens_1d	143.716 (0.012)	64.396 (0.038)	34.350 (0.070)
Baum_60_1	71.173 (0.005)	28.222 (0.023)	27.896 (0.112)
Baum_60_3	69.163 (0.014)	43.407 (0.079)	44.745 (0.430)
Baum_60_4	51.843 (0.040)	51.431 (0.052)	53.587 (0.056)

BIC

Dataset	2C	RM	EM
Fokkens_1a	71.326 (0.007)	77.417 (0.056)	74.387 (0.029)
Fokkens_1e	12.261 (0.025)	9.954 (0.009)	13.324 (0.025)
Baum_60_2	53.243 (0.022)	53.665 (0.063)	57.253 (0.025)
Fokkens_1c	-25.708 (0.013)	-22.517 (0.290)	-47.959 (0.100)
Baum_57	102.851 (0.007)	81.841 (0.147)	57.076 (0.038)
Baum_59	286.106 (0.017)	243.215 (0.008)	133.718 (0.069)
Fokkens_1b	87.095 (0.014)	85.541 (0.164)	42.715 (0.062)
Fokkens_1d	150.784 (0.013)	73.821 (0.040)	44.953 (0.070)
Baum_60_1	76.840 (0.005)	35.777 (0.022)	36.396 (0.111)
Baum_60_3	75.137 (0.014)	51.373 (0.083)	52.706 (0.428)
Baum_60_4	60.046 (0.040)	62.569 (0.052)	65.893 (0.056)
