# Supplementary material for: Bayesian regression and model selection for isothermal titration calorimetry with enantiomeric mixtures
Source: PLoS One. 2022 Sep 29;17(9):e0273656. doi: 10.1371/journal.pone.0273656 (PMC9521810; doi:10.1371/journal.pone.0273656)
Supplement: S3 Table — Binding enthalpy (ΔH, ΔH1, ΔH2) and free energy (ΔG, ΔG1, ΔΔG) in kcal/mol. (PDF) [file pone.0273656.s007.pdf]

**Two-Component model is the best**	Two-Component model	Racemic-Mixture model	Enantiomer model
Fokkens_1a	$\Delta H \in$ [-2.08, -0.61]<br>$\Delta G \in$ [-7.66, -6.95]	$\Delta H_1 \in$ [-79.61, 66.84]<br>$\Delta H_2 \in$ [-49.60, -3.19]<br>$\Delta G_1 \in$ [-39.05, -13.96]<br>$\Delta G_2 \in$ [-9.20, -7.53]	$\Delta H_1 \in$ [-4.15, -0.75]<br>$\Delta H_2 \in$ [-85.66, 95.69]<br>$\Delta G_1 \in$ [-8.09, -7.03]<br>$\Delta G_2 \in$ [-2.68, 3.10]<br>$\rho \in$ [0.20, 0.99]
Fokkens_1e	$\Delta H \in$ [-0.99, -0.75]<br>$\Delta G \in$ [-7.21, -6.93]	$\Delta H_1 \in$ [-70.94, -1.15]<br>$\Delta H_2 \in$ [-0.78, 73.99]<br>$\Delta G_1 \in$ [-8.52, -7.00]<br>$\Delta G_2 \in$ [-7.26, -2.11]	$\Delta H_1 \in$ [-1.26, -0.84]<br>$\Delta H_2 \in$ [-78.14, 96.36]<br>$\Delta G_1 \in$ [-7.25, -6.78]<br>$\Delta G_2 \in$ [-3.66, 3.00]<br>$\rho \in$ [0.61, 0.98]
Baum_60_2	$\Delta H \in$ [-13.41, -8.89]<br>$\Delta G \in$ [-8.40, -7.89]	$\Delta H_1 \in$ [-20.63, -10.52]<br>$\Delta H_2 \in$ [-95.91, 81.31]<br>$\Delta G_1 \in$ [-9.25, -8.24]<br>$\Delta G_2 \in$ [-7.84, 1.46]	$\Delta H_1 \in$ [-70.28, 78.88]<br>$\Delta H_2 \in$ [-90.79, 87.45]<br>$\Delta G_1 \in$ [-37.82, -8.05]<br>$\Delta G_2 \in$ [-8.27, 1.58]<br>$\rho \in$ [0.02, 0.98]
**Enantiomer model is the best**			
Fokkens_1c	$\Delta H \in$ [-5.30, -4.04]<br>$\Delta G \in$ [-10.57, -10.17]	$\Delta H_1 \in$ [-13.00, 13.37]<br>$\Delta H_2 \in$ [-5.36, -2.99]<br>$\Delta G_1 \in$ [-39.18, -10.54]<br>$\Delta G_2 \in$ [-10.26, -9.76]	$\Delta H_1 \in$ [-5.69, -4.16]<br>$\Delta H_2 \in$ [-97.35, -5.40]<br>$\Delta G_1 \in$ [-10.80, -10.49]<br>$\Delta G_2 \in$ [-7.19, -4.44]

			$\rho \in [0.71, 0.99]$
Baum_57	$\Delta H \in [-8.14, -0.79]$<br>$\Delta G \in [-6.17, -4.47]$	$\Delta H_1 \in [-11.69, -0.66]$<br>$\Delta H_2 \in [-67.25, 81.24]$<br>$\Delta G_1 \in [-22.57, -5.21]$<br>$\Delta G_2 \in [-7.03, 3.16]$	$\Delta H_1 \in [-6.60, -0.77]$<br>$\Delta H_2 \in [-4.01, -0.42]$<br>$\Delta G_1 \in [-11.33, -9.48]$<br>$\Delta G_2 \in [-6.96, -5.65]$<br>$\rho \in [0.44, 0.47]$
Baum_59	$\Delta H \in [-4.76, -3.20]$<br>$\Delta G \in [-7.89, -6.99]$	$\Delta H_1 \in [-8.47, -5.78]$<br>$\Delta H_2 \in [-97.38, -8.55]$<br>$\Delta G_1 \in [-9.10, -8.35]$<br>$\Delta G_2 \in [-4.12, -2.44]$	$\Delta H_1 \in [-30.59, -19.38]$<br>$\Delta H_2 \in [-2.57, -1.46]$<br>$\Delta G_1 \in [-10.89, -10.46]$<br>$\Delta G_2 \in [-6.55, -5.97]$<br>$\rho \in [0.13, 0.17]$
**Racemic-Mixture and Enantiomer models are equally best**			
Fokkens_1b	$\Delta H \in [-48.68, -0.87]$<br>$\Delta G \in [-5.70, -3.01]$	$\Delta H_1 \in [-4.81, 23.08]$<br>$\Delta H_2 \in [-96.89, 33.22]$<br>$\Delta G_1 \in [-38.41, -4.65]$<br>$\Delta G_2 \in [-5.53, 1.91]$	$\Delta H_1 \in [-12.04, 22.45]$<br>$\Delta H_2 \in [-95.14, -0.10]$<br>$\Delta G_1 \in [-37.94, -5.75]$<br>$\Delta G_2 \in [-8.65, -0.98]$<br>$\rho \in [0.06, 0.86]$
Fokkens_1d	$\Delta H \in [-98.81, -42.59]$<br>$\Delta G \in [-4.93, -4.37]$	$\Delta H_1 \in [-7.55, -5.73]$<br>$\Delta H_2 \in [-3.37, -2.19]$<br>$\Delta G_1 \in [-12.28, -10.84]$<br>$\Delta G_2 \in [-7.41, -6.66]$	$\Delta H_1 \in [-8.15, -6.21]$<br>$\Delta H_2 \in [-2.55, -1.87]$<br>$\Delta G_1 \in [-11.62, -11.12]$<br>$\Delta G_2 \in [-7.58, -7.15]$<br>$\rho \in [0.44, 0.46]$

Baum_60_1	$\Delta H \in$ [-12.01, -8.16]<br>$\Delta G \in$ [-8.59, -8.09]	$\Delta H_1 \in$ [-29.72, -10.17]<br>$\Delta H_2 \in$ [-9.59, 9.70]<br>$\Delta G_1 \in$ [-10.91, -8.99]<br>$\Delta G_2 \in$ [-8.78, -8.24]	$\Delta H_1 \in$ [-85.93, -11.03]<br>$\Delta H_2 \in$ [-8.89, 43.17]<br>$\Delta G_1 \in$ [-10.86, -8.82]<br>$\Delta G_2 \in$ [-8.96, -8.31]<br>$\rho \in$ [0.09, 0.73]
**Inconclusive**			
Baum_60_3	$\Delta H \in$ [-11.55, -7.55]<br>$\Delta G \in$ [-9.11, -8.28]	$\Delta H_1 \in$ [-94.84, -22.09]<br>$\Delta H_2 \in$ [2.80, 74.04]<br>$\Delta G_1 \in$ [-16.02, -9.66]<br>$\Delta G_2 \in$ [-15.61, -9.27]	$\Delta H_1 \in$ [-96.43, -17.96]<br>$\Delta H_2 \in$ [-1.81, 87.22]<br>$\Delta G_1 \in$ [-16.09, -9.43]<br>$\Delta G_2 \in$ [-15.65, -8.93]<br>$\rho \in$ [0.24, 0.76]
Baum_60_4	$\Delta H \in$ [-4.47, -0.98]<br>$\Delta G \in$ [-7.56, -6.48]	$\Delta H_1 \in$ [-16.91, 7.70]<br>$\Delta H_2 \in$ [-93.99, 85.36]<br>$\Delta G_1 \in$ [-34.21, -6.96]<br>$\Delta G_2 \in$ [-7.72, 2.99]	$\Delta H_1 \in$ [-12.64, -1.22]<br>$\Delta H_2 \in$ [-94.41, 90.31]<br>$\Delta G_1 \in$ [-8.73, -6.69]<br>$\Delta G_2 \in$ [-3.86, 3.28]<br>$\rho \in$ [0.09, 0.98]
