# Supplementary figures and images for: Bayesian regression and model selection for isothermal titration calorimetry with enantiomeric mixtures
Source: PLoS One. 2022 Sep 29;17(9):e0273656. doi: 10.1371/journal.pone.0273656 (PMC9521810; doi:10.1371/journal.pone.0273656)

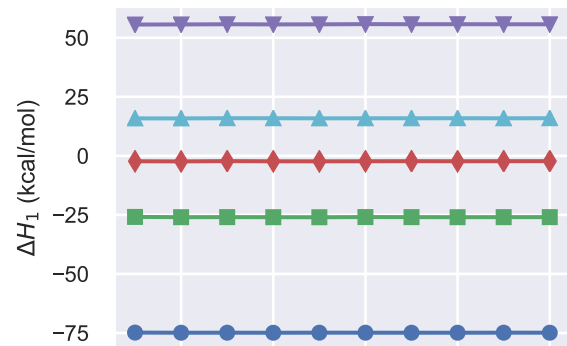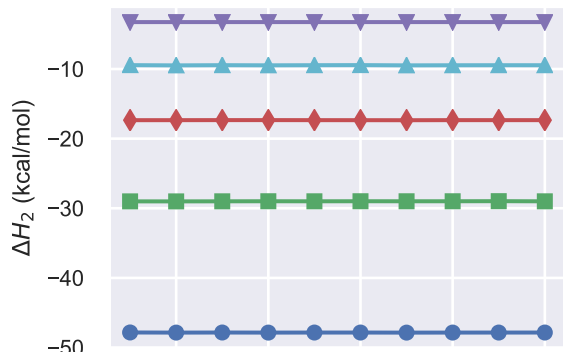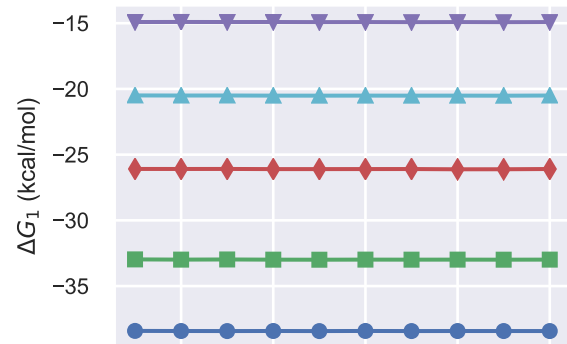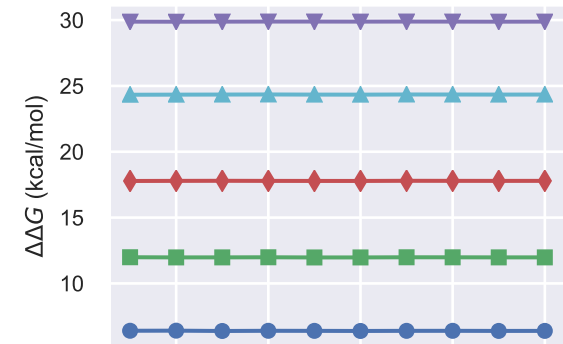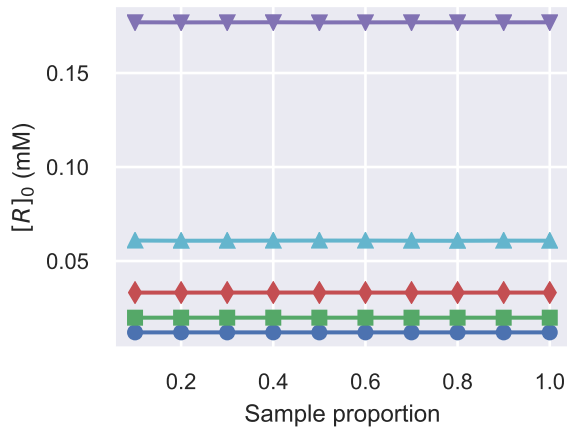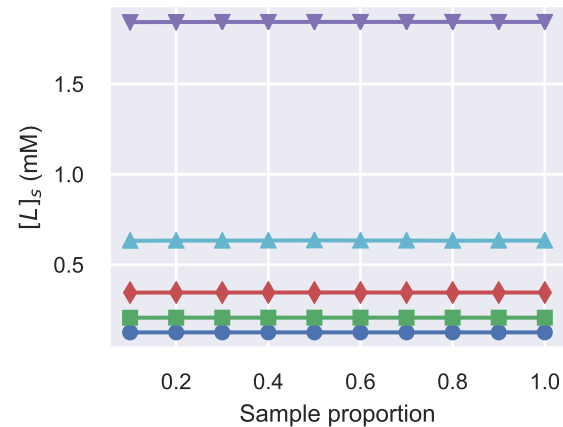

Supplement: S1 Fig — 60,000 samples were drawn from the Bayesian posterior using the NUTS sampler. Six key parameters are shown. Lines correspond to the 5-th (blue circle), 25-th (green square), 50-th (red diamond), 75-th (cyan upward triangle) and 95-th (magenta downward triangle) percentile. The error bars, which are too small to be visible, are standard deviations estimated by 100 bootstrapping samples. (PDF) [file pone.0273656.s008.pdf]

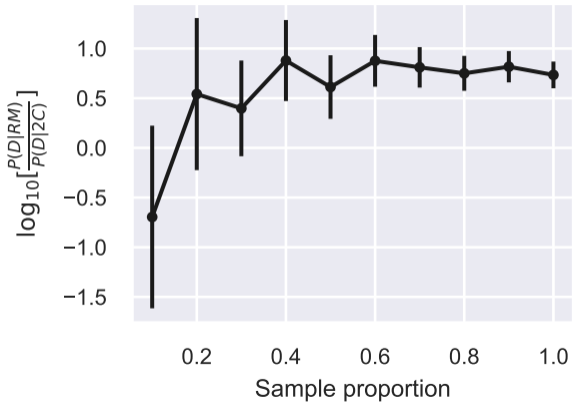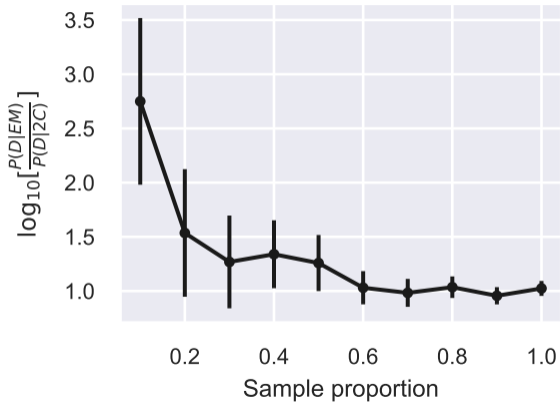

Supplement: S3 Fig — The Bayes factors were estimated based on 20,000 NUTS samples for the 2C model and 60,000 NUTS samples for the RM and EM models. The error bars are standard deviations estimated by 1000 bootstrapping samples. (PDF) [file pone.0273656.s010.pdf]

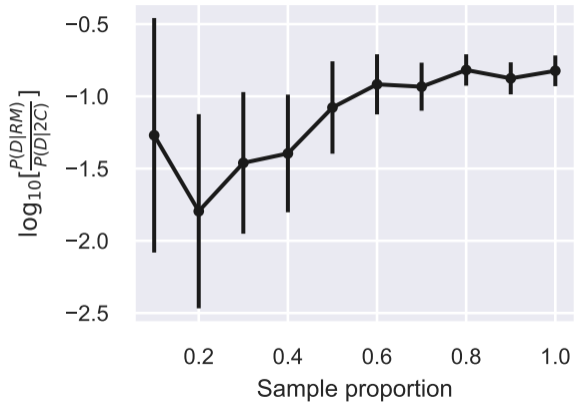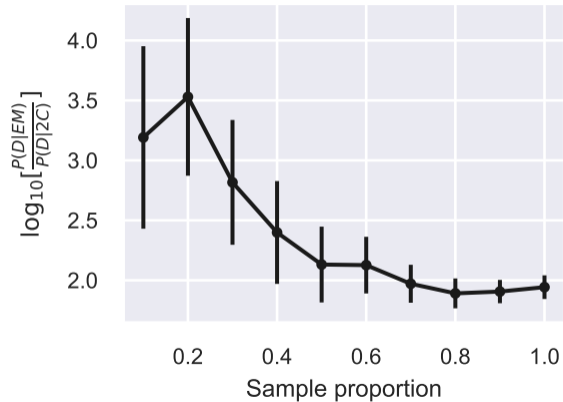

Supplement: S4 Fig — The Bayes factors were estimated based on 20,000 NUTS samples for the 2C model and 60,000 NUTS samples for the RM and EM models. The error bars are standard deviations estimated by 1000 bootstrapping samples. (PDF) [file pone.0273656.s011.pdf]

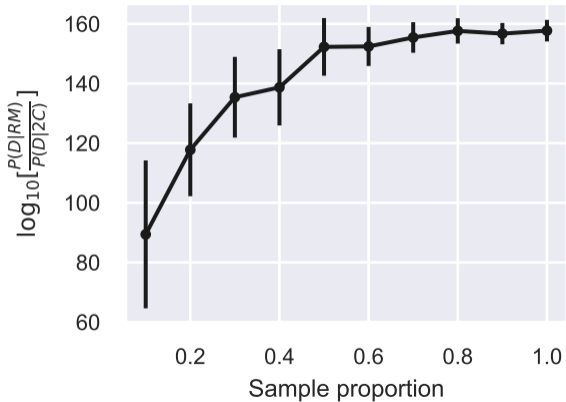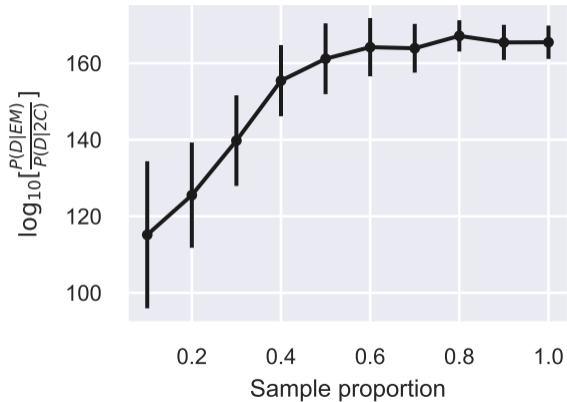

Supplement: S5 Fig — The Bayes factors were estimated based on 20,000 NUTS samples for the 2C model and 60,000 NUTS samples for the RM and EM models. The error bars are standard deviations estimated by 1000 bootstrapping samples. (PDF) [file pone.0273656.s012.pdf]

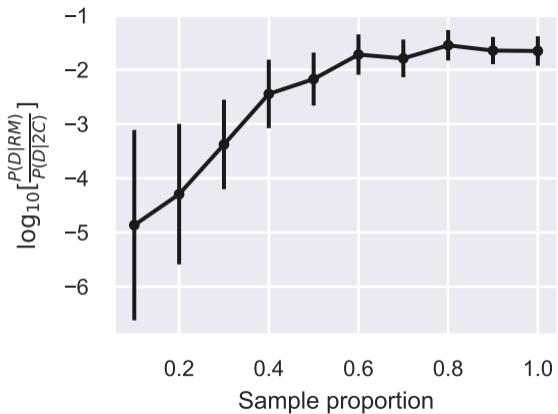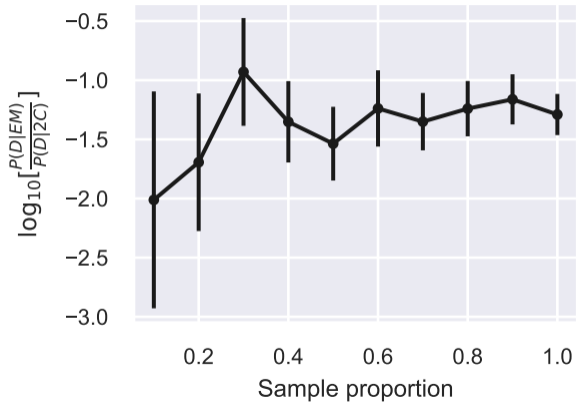

Supplement: S6 Fig — The Bayes factors were estimated based on 20,000 NUTS samples for the 2C model and 60,000 NUTS samples for the RM and EM models. The error bars are standard deviations estimated by 1000 bootstrapping samples. (PDF) [file pone.0273656.s013.pdf]

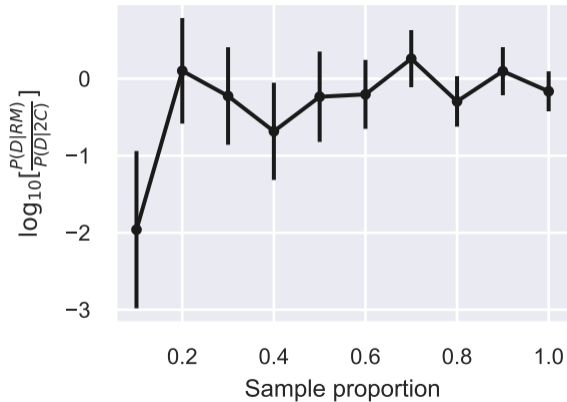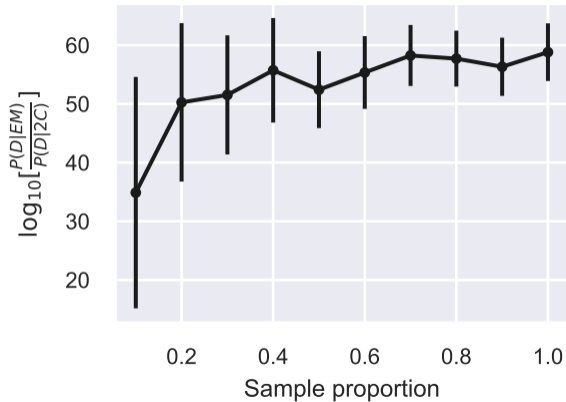

Supplement: S7 Fig — The Bayes factors were estimated based on 20,000 NUTS samples for the 2C model and 60,000 NUTS samples for the RM and EM models. The error bars are standard deviations estimated by 1000 bootstrapping samples. (PDF) [file pone.0273656.s014.pdf]

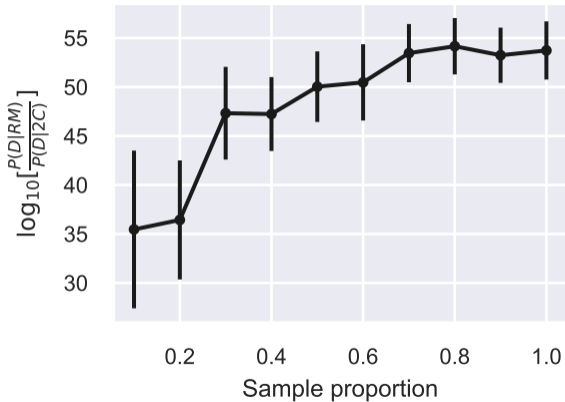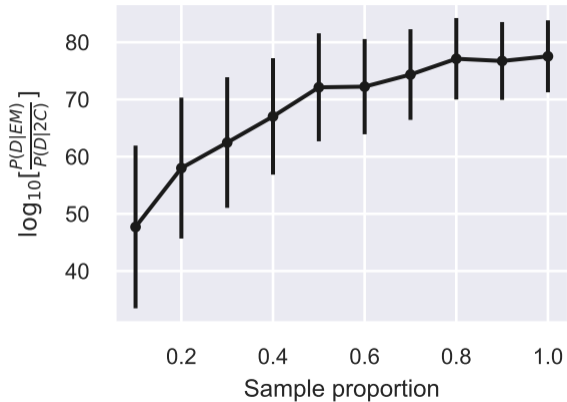

Supplement: S8 Fig — The Bayes factors were estimated based on 20,000 NUTS samples for the 2C model and 60,000 NUTS samples for the RM and EM models. The error bars are standard deviations estimated by 1000 bootstrapping samples. (PDF) [file pone.0273656.s015.pdf]

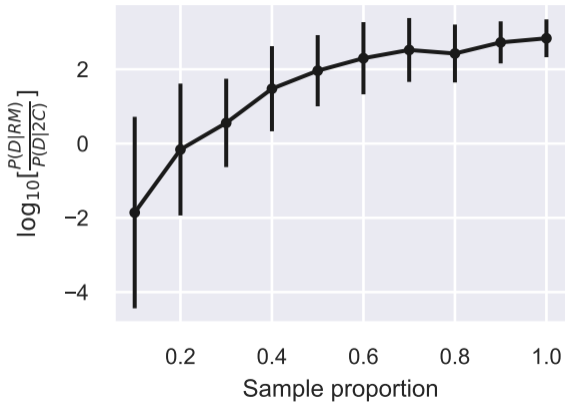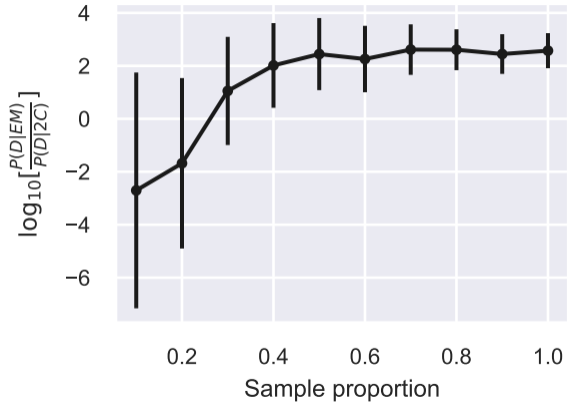

Supplement: S9 Fig — The Bayes factors were estimated based on 20,000 NUTS samples for the 2C model and 60,000 NUTS samples for the RM and EM models. The error bars are standard deviations estimated by 1000 bootstrapping samples. (PDF) [file pone.0273656.s016.pdf]

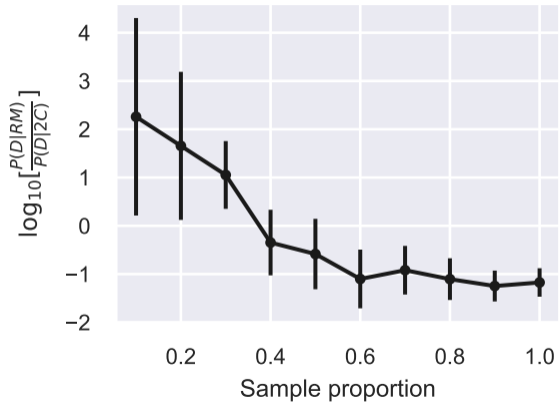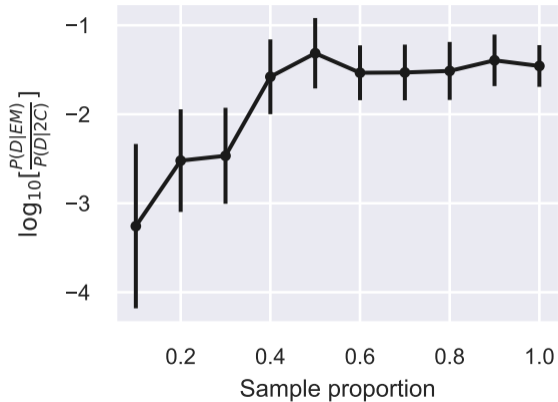

Supplement: S10 Fig — The Bayes factors were estimated based on 20,000 NUTS samples for the 2C model and 60,000 NUTS samples for the RM and EM models. The error bars are standard deviations estimated by 1000 bootstrapping samples. (PDF) [file pone.0273656.s017.pdf]

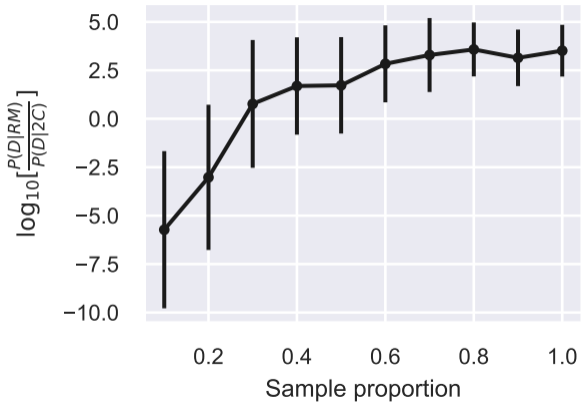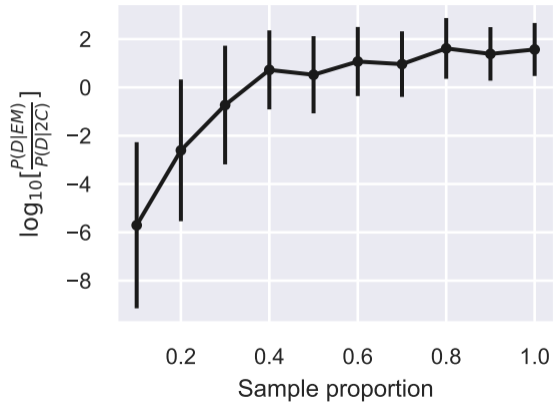

Supplement: S11 Fig — The Bayes factors were estimated based on 20,000 NUTS samples for the 2C model and 60,000 NUTS samples for the RM and EM models. The error bars are standard deviations estimated by 1000 bootstrapping samples. (PDF) [file pone.0273656.s018.pdf]

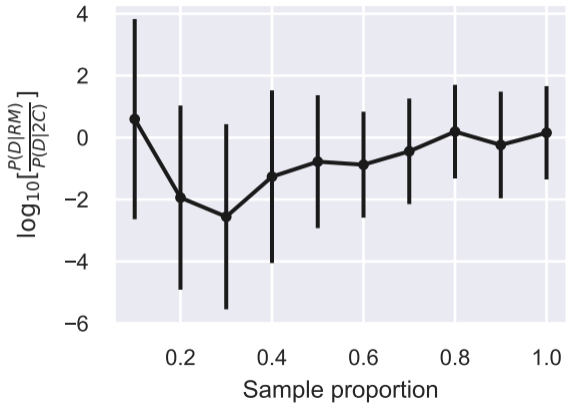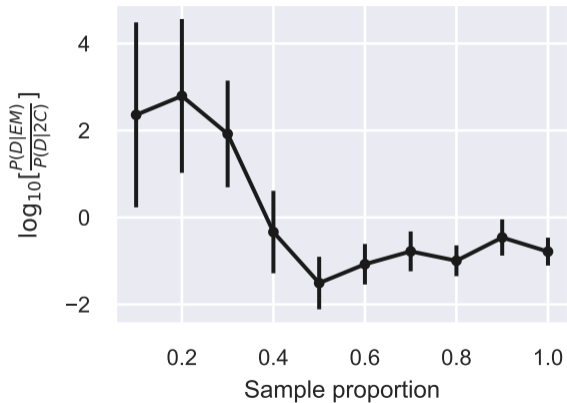

Supplement: S12 Fig — The Bayes factors were estimated based on 20,000 NUTS samples for the 2C model and 60,000 NUTS samples for the RM and EM models. The error bars are standard deviations estimated by 1000 bootstrapping samples. (PDF) [file pone.0273656.s019.pdf]
