# Supplementary material for: Bayesian regression and model selection for isothermal titration calorimetry with enantiomeric mixtures
Source: PLoS One. 2022 Sep 29;17(9):e0273656. doi: 10.1371/journal.pone.0273656 (PMC9521810; doi:10.1371/journal.pone.0273656)

Fokkens 1a

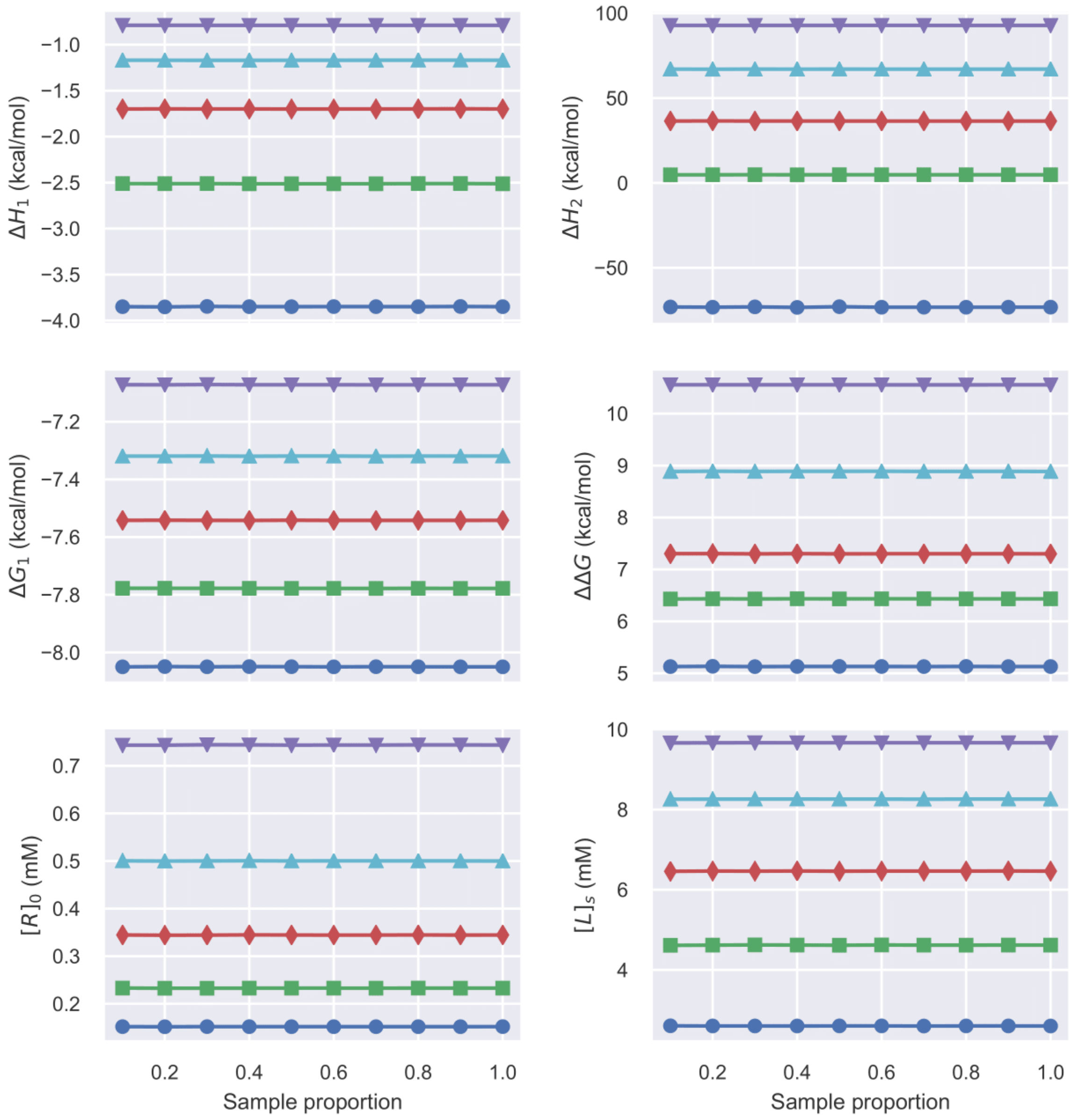

−1.0
−1.5
−2.0
−2.5
−3.0
−3.5
−4.0
ΔH1 (kcal/mol)
100
50
0
−50
ΔH2 (kcal/mol)
−7.2
−7.4
−7.6
−7.8
−8.0
ΔG1 (kcal/mol)
10
9
8
7
6
5
ΔΔG (kcal/mol)
0.7
0.6
0.5
0.4
0.3
0.2
[R]0 (mM)
10
8
6
4
[L]s (mM)
0.2
0.4
0.6
0.8
1.0
Sample proportion
0.2
0.4
0.6
0.8
1.0
Sample proportion

Simulation Data - Priors for the concentration were lognormal for both $[L]_s$ and $[R]_0$

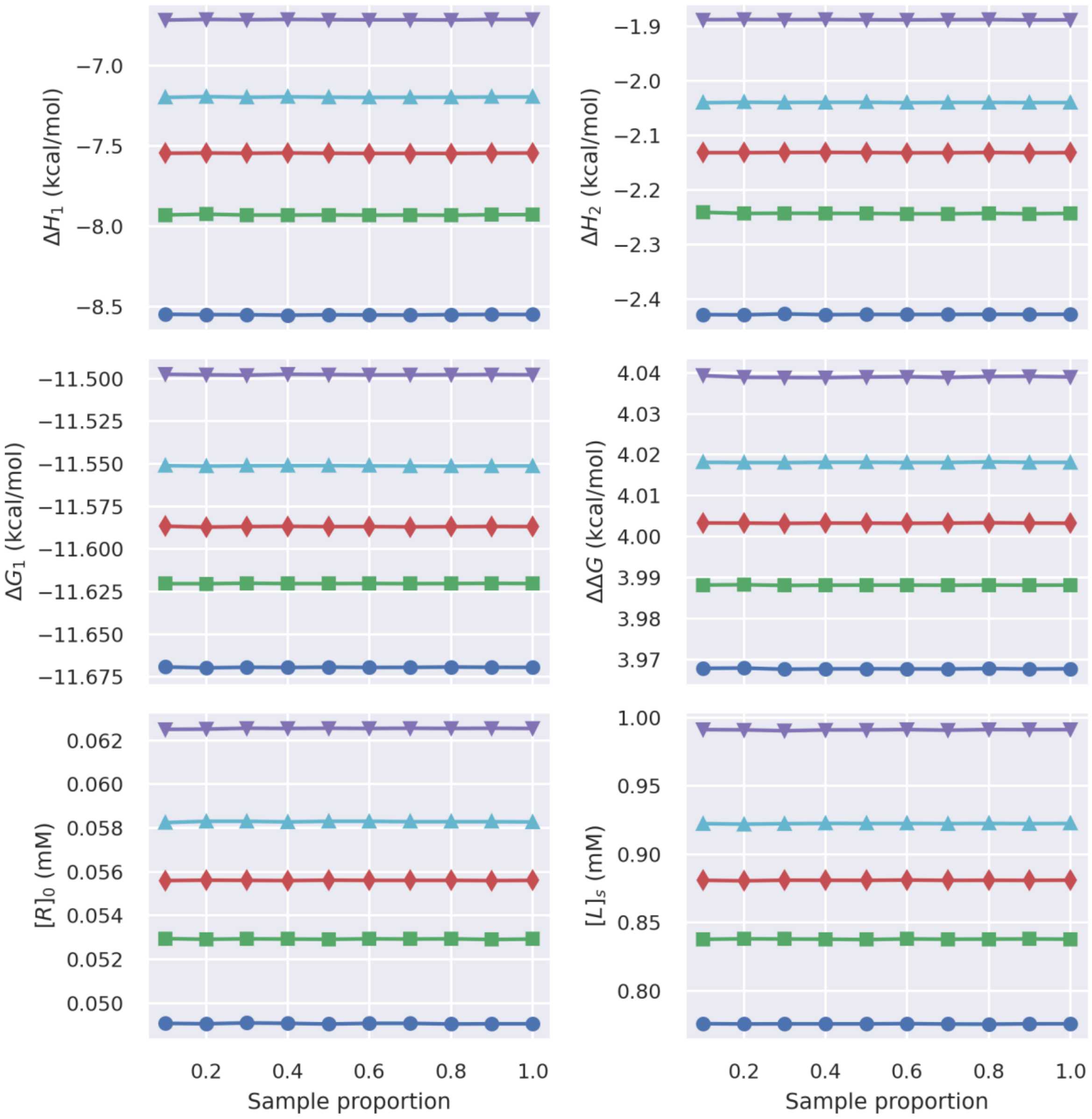

Simulation Data - Priors for the concentration were lognormal for $[L]_s$ and uniform for $[R]_0$

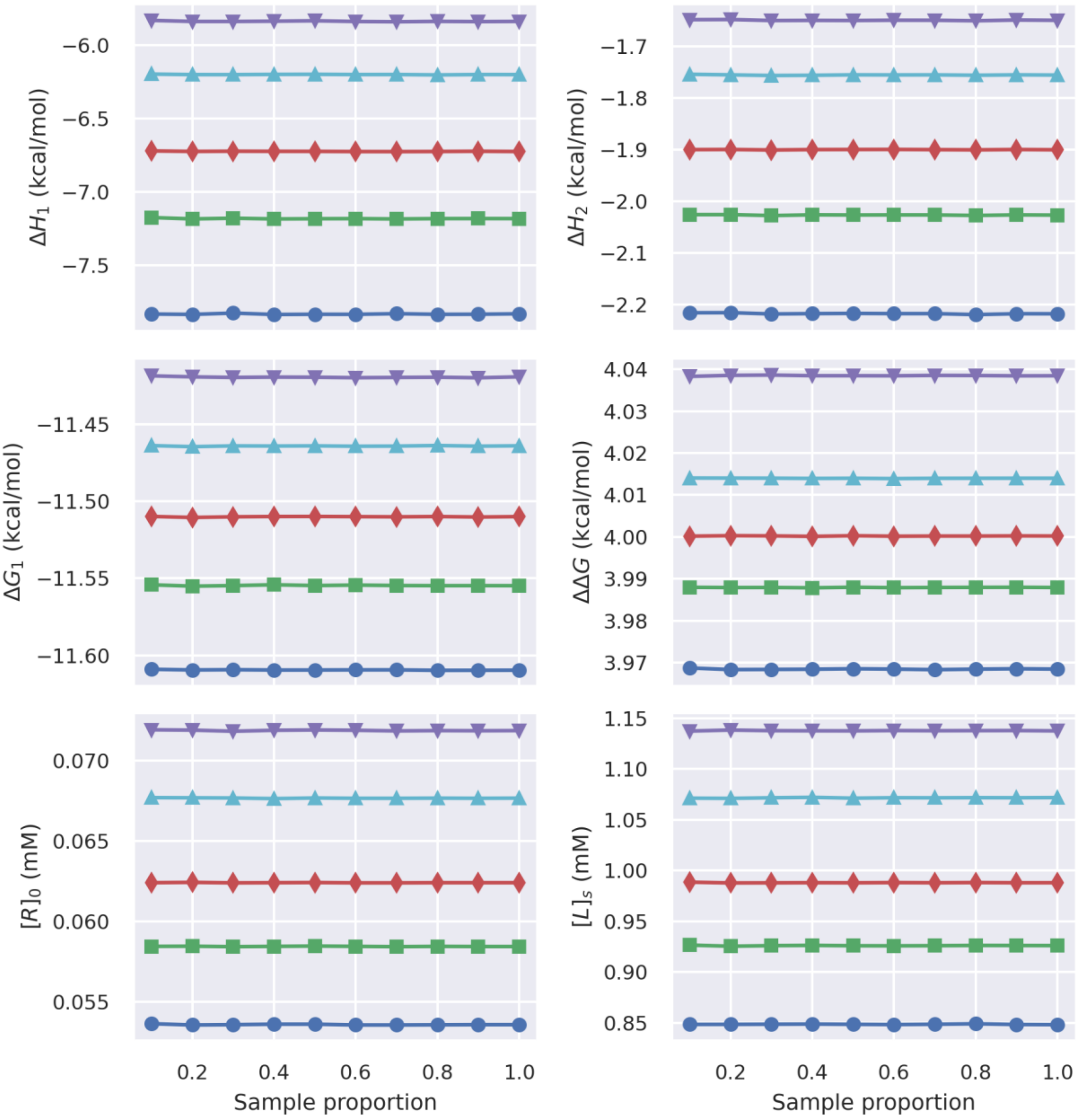

Simulation Data - Priors for the concentration were uniform for $[L]_s$ and lognormal for $[R]_0$

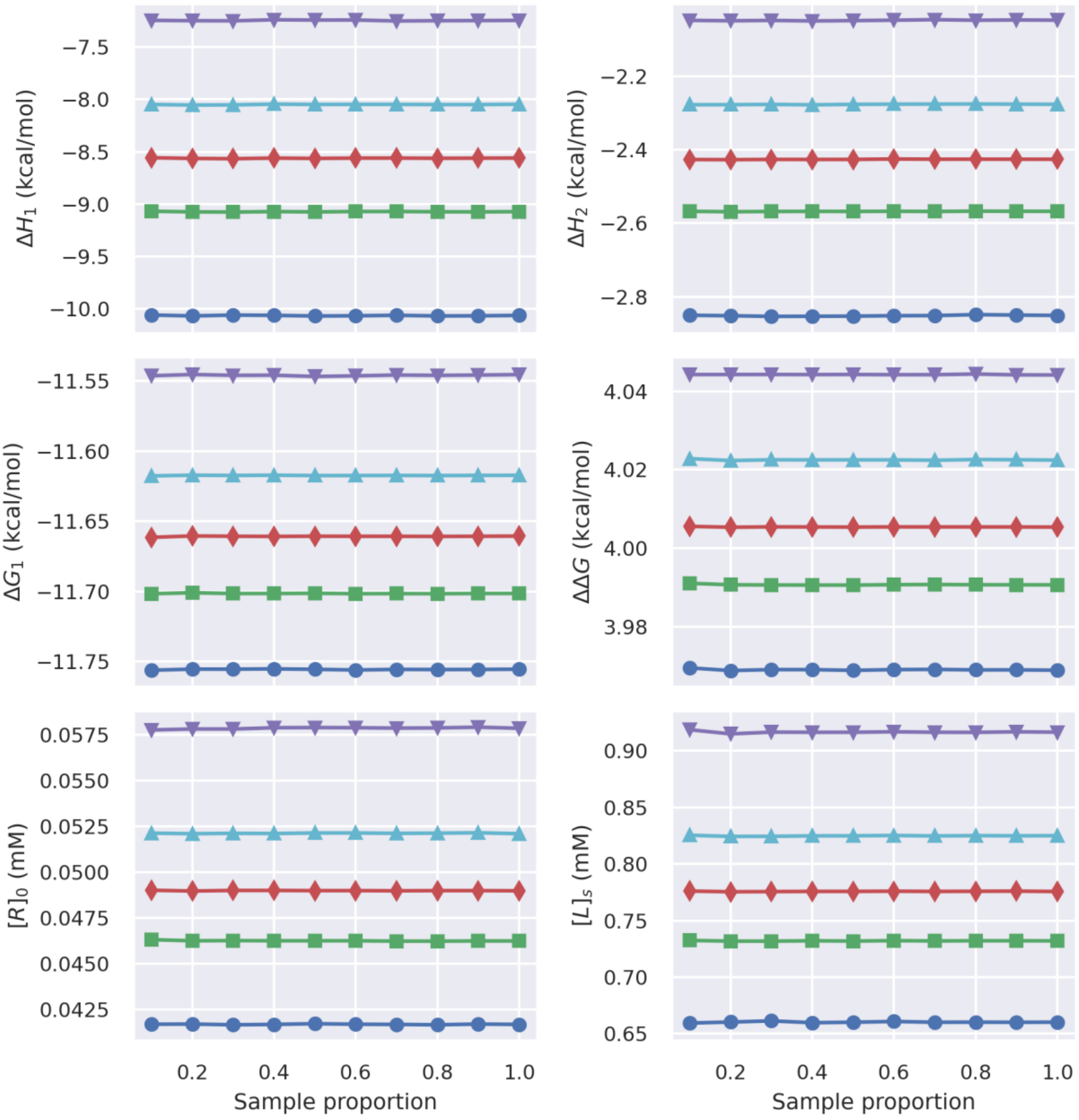

Simulation Data - Priors for the concentration were uniform for both $[L]_s$ and $[R]_0$

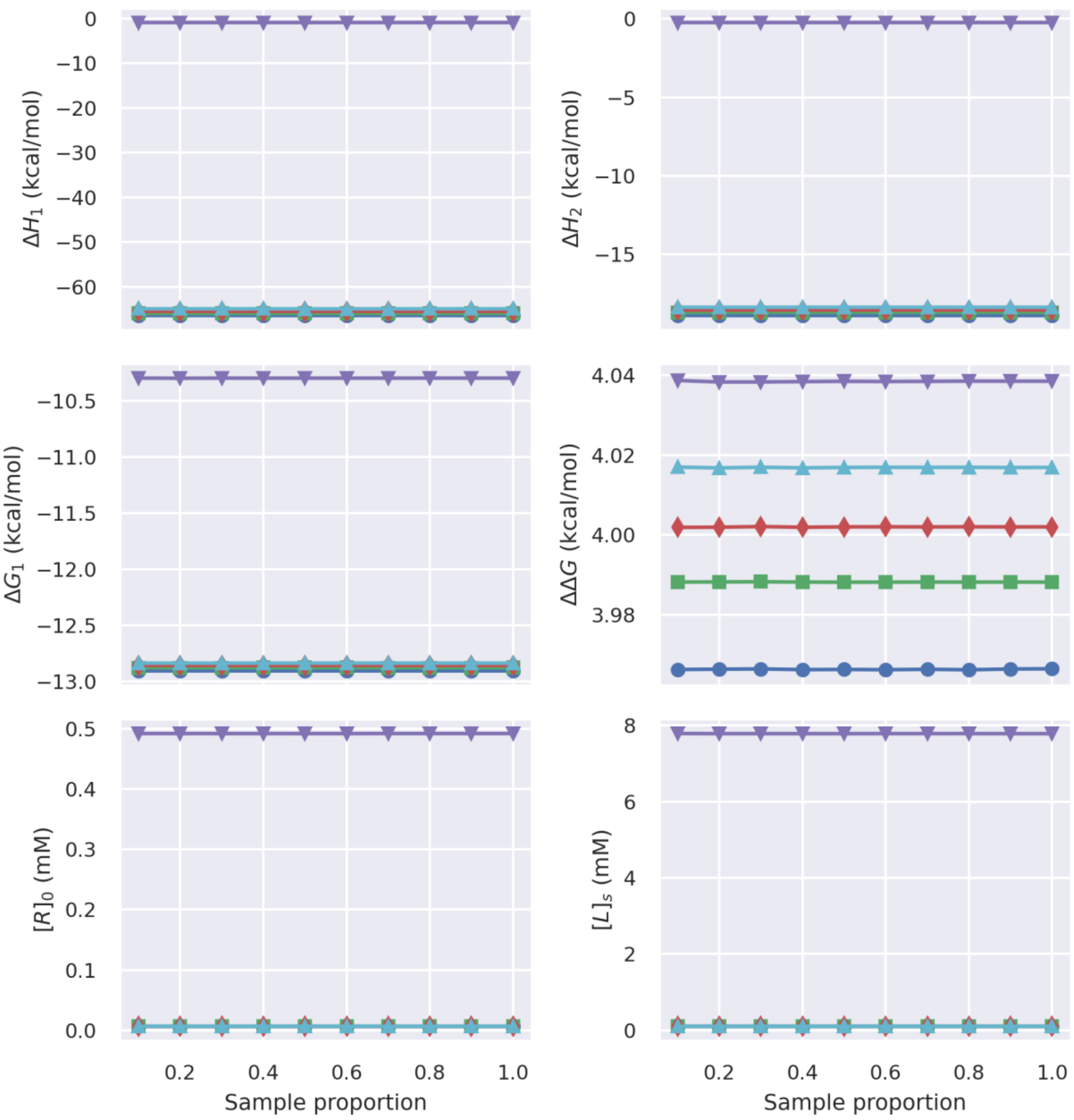

Supplement: S2 Fig — 60,000 samples were drawn from the Bayesian posterior using the NUTS sampler. Six key parameters are shown. Lines correspond to the 5-th (blue circle), 25-th (green square), 50-th (red diamond), 75-th (cyan upward triangle) and 95-th (magenta downward triangle) percentile. The error bars, which are too small to be visible, are standard deviations estimated by 100 bootstrapping samples. (PDF) [file pone.0273656.s009.pdf]
